# Medicinal Plants Recommended by the World Health Organization: DNA Barcode Identification Associated with Chemical Analyses Guarantees Their Quality

**DOI:** 10.1371/journal.pone.0127866

**Published:** 2015-05-15

**Authors:** Rafael Melo Palhares, Marcela Gonçalves Drummond, Bruno dos Santos Alves Figueiredo Brasil, Gustavo Pereira Cosenza, Maria das Graças Lins Brandão, Guilherme Oliveira

**Affiliations:** 1 Programa de Pós-graduação em Genética, Universidade Federal de Minas Gerais, Belo Horizonte, Brazil; 2 Myleus Biotechnology Research Team, Belo Horizonte, Brazil; 3 Grupo de Genômica e Biologia Computacional, Centro de Pesquisa Rene Rachou, FIOCRUZ, Belo Horizonte, Brasil; 4 EMBRAPA Agroenergia, Brasilia, Brazil; 5 Laboratório de Farmacognosia, Faculdade de Farmácia, Universidade Federal de Minas Gerais, Belo Horizonte, Brasil; 6 CEPLAMT, Museu de História Natural e Jardim Botânico & Faculdade de Farmácia, Universidade Federal de Minas Gerais, Belo Horizonte, Brasil; Biodiversity Insitute of Ontario - University of Guelph, CANADA

## Abstract

Medicinal plants are used throughout the world, and the regulations defining their proper use, such as identification of the correct species and verification of the presence, purity and concentration of the required chemical compounds, are widely recognized. Herbal medicines are made from vegetal drugs, the processed products of medicinal species. These processed materials present a number of challenges in terms of botanical identification, and according to the World Health Organization (WHO), the use of incorrect species is a threat to consumer safety. The samples used in this study consisted of the dried leaves, flowers and roots of 257 samples from 8 distinct species approved by the WHO for the production of medicinal herbs and sold in Brazilian markets. Identification of the samples in this study using DNA barcoding (*matK*, *rbcL* and *ITS2* regions) revealed that the level of substitutions may be as high as 71%. Using qualitative and quantitative chemical analyses, this study identified situations in which the correct species was being sold, but the chemical compounds were not present. Even more troubling, some samples identified as substitutions using DNA barcoding contained the chemical compounds from the correct species at the minimum required concentration. This last situation may lead to the use of unknown species or species whose safety for human consumption remains unknown. This study concludes that DNA barcoding should be used in a complementary manner for species identification with chemical analyses to detect and quantify the required chemical compounds, thus improving the quality of this class of medicines.

## Introduction

The global market of products derived from plants is estimated at $83 billion US and continues to grow [1]. Furthermore, it is estimated that approximately 25% of modern drugs and as many as 60% of antitumor drugs [2] are derived from natural products [3]. According to the WHO, between 65% and 80% of the populations of developing countries currently use medicinal plants as remedies [1]. The development of new products from natural sources is also encouraged because it is estimated that of the 300,000 plant species that exist in the world, only 15% have been evaluated to determine their pharmacological potential [4]. Studies demonstrating the efficacy and importance of medicinal plants are being carried out worldwide in countries that span a wide range of developmental stages [5–9]. Due to the widespread use of medicinal plants, the WHO published the Monographs on Selected Medicinal Plants volumes 1 through 5 from 1999 to 2010; these volumes contain a list of species with recognized medicinal benefits and the accepted means to correctly use them [10–14]. In addition to following WHO recommendations, Brazil has its own agency that regulates the use of medicinal plants, the National Health Surveillance Agency (from the Portuguese ANVISA—Agência Nacional de Vigilância Sanitária). ANVISA also has its own list of approved species for manufacturing herbal medicines [15]. Although Brazil is rich in biodiversity and medicinal plants, most of these plants on this list are exotic species that were introduced to the country during the early phases of European colonization in the 1500s [16–18].

To guarantee the quality of herbal medicines, certain steps established in the Pharmacopoeias must be followed, including correct identification of the plant species, analysis of the purity and confirmation of the presence and minimum concentration of the active ingredients (chemical marker(s)) [19]. In this regard, one of the main challenges encountered in the herbal medicine industry is ensuring unequivocal species identification of the raw material that will be used to manufacture the herbal medicine. There are several plant identification techniques, but in many cases, the identification is based mainly on botanical analysis, that can be problematic due to the high phenotypic variation among taxa, the commercialization of processed raw plant material and/or unidentifiable plant parts and the lack of highly trained professionals in plant taxonomy [20–22]. Furthermore, quality control for herbal drugs is currently performed according to a set of pharmaceutical analyses, beginning by direct observation of the morphological, sensory and microscopic characteristics of each type of plant material. If the identity of the plant part is verified, the sample is submitted for chemical characterization using chromatographic methods to verify the presence of specific substances in comparison with a chemical profile in the literature or found in standard samples [23–27]. Misidentification and substitutions are a reality, with confirmed reports from several countries [7, 28–30], including a recent study from our group in which we demonstrated substitutions of species of “quina” (*Cinchona* spp.) in Brazilian markets [31]. These issues are of high concern because they may cause fatalities among users [32, 33]. Under these conditions, DNA barcoding may be a powerful tool. The DNA barcode consists of one or more short, standardized DNA region(s) that can be used to identify a species [34]. and is a powerful tool that can be applied to address the problems in botanically identifying highly processed plant materials [35–38] in addition to other uses, such as the identification of endangered species and the use in forensic DNA researches [39, 40]. Since 2009 the Plant Working Group from the Barcode of Life project established that the official regions for DNA Barcodes of plants are *rbcL* and *matK* [41]. Despite this, those regions are not 100% efficient in discriminating plant species and other regions are used by different researchers to improve the efficiency of the official DNA Barcode [42–44].

Here we propose the use of DNA barcoding technology to identify the raw material used to manufacture herbal medicines. Along with the CBOL recommendations and based on previous studies, we evaluated the addition of the nuclear *ITS2* region to the barcode core of *matK* and *rbcL* [20]. After the initial identification step, our group carried out chemical analyses to demonstrate the presence and concentration of the essential chemical compound of the herbal medicine. Our results indicate that DNA barcoding should be used as a screening step during the herbal medicine manufacturing process, and only samples that are correctly identified should proceed to chemical validation. This proposed workflow would improve the safety, speed and reliability of this process.

## Materials and Methods

### Sample collection

A total of 257 samples from 8 species that are recognized by the WHO and ANVISA as medicinal species and approved for use in the preparation of remedies were purchased in the Central Market (19° 55' 22.465" S 43° 56' 35.058" W) in the city of Belo Horizonte in the state of Minas Gerais, Brazil. Belo Horizonte’s metropolitan region holds approximately 6 million inhabitants and possesses a large traditional popular market as well as several drugstores and pharmacies specialized in phytoterapy and medicinal plants. The collectors purchased the samples, as regular customers, from 20 stores, during the years of 2012 to 2014. Products sold as dried plant parts or as powdered tissues, either simply packed or encapsulated, were sampled. Samples were stored in an acclimatized and humidity free room before DNA extraction. The studied species, popular names and used parts were *Hamamelis virginiana* L. (Hamamelis—leaves), *Matricaria recutita* L. (Chamomile, flowers), *Maytenus ilicifolia* Mart. Ex Reiss (Espinheira Santa, leaves), *Mikania glomerata* Spreng. (Guaco, leaves), *Panax ginseng* C. A. Mey (Asian Ginseng, roots), *Passiflora incarnata* L. (passion flower, leaves), *Peumus boldus* Molina (Boldo-do-Chile, leaves) and *Valeriana officinalis* L. (Valerian, roots) (Table 1).

**Table 1:** Species analyzed in this study and their therapeutical recommendations.

Species	Recommended uses			Number of samples
*H*. *virginiana* L.	Topically for minor skin lesions, bruises and sprains, local inflammation of the skin and mucous membranes, hemorrhoids and varicose veins [11]			32
	**Internal uses**	**External uses**	**Inhalation**	
*M*. *recutita* L.	Symptomatic treatment of digestive ailments, treatment of restlessness and insomnia due to nervous disorders [10]	Inflammation and irritations of the skin and mucosa, including irritations and infections of the mouth and gums, and hemorrhoids [10]	Symptomatic relief on irritations of the respiratory tract due to common cold [10]	31
*M*. *ilicifolia* Mart. Ex Reiss	Treatment of dyspepsia, gastritis and gastroduodenal ulcer [15]			33
*M*. *glomerata* Sprengl.	Bronchodilatador and expectorant [15]			31
*P*. *ginseng* C. A. Mey	Prophylactic and restorative agent for enhancement of mental and physical capacities, in cases of weakness, exhaustion, tiredness, and loss of concentration, and during convalescence [10]			31
*P*. *incarnata* L.	Mild sedative for nervous restlessness, insomnia and anxiety. Treatment of gastrointestinal disorders of nervous origins [12]			30
*P*. *boldus* Molina	Treatment of functional dyspepsia and gastrointestinal disorders, cholagogue and choleretic [15]			34
*V*. *officinalis* L.	Mild sedative and sleep promoting agent. Often used as a milder alternative or a possible substitute for stronger synthetic sedatives in treatment of nervous excitation and anxiety-induced sleep disturbances [10]			35

### Characteristics of the samples

The acquired samples included, flowers, leaves and roots. The samples were collected in two forms, as the dried parts described above and as powdered tissues. No mixtures were analyzed due to limitations inherent to the Sanger sequencing method. In the laboratory, each sample was recorded and kept under uniform conditions in a climate-controlled room at DATAPLAMT (Aromatic, medicinal and poisonous center for data and sample storage at the Universidade Federal de Minas Gerais).

### DNA extraction

DNA was extracted from the leaves, flowers and roots of the plants using the DNeasy plant mini kit (Qiagen, Venlo—Netherlands) with modifications. Approximately 20 mg of each sample was pulverized using a mortar at room temperature. The powder was mixed with 600 μL of buffer AP1 supplied with the kit and incubated at 65°C and 400 rpm for 1 hour in a heat block (Thermomixer compact; Eppendorf, Germany). After incubation, 230 μL of buffer AP2 from the DNeasy kit was added, and the samples were incubated on ice for 30 minutes. The later steps of the extraction were carried out following instructions from the manufacturer (DNeasy plant handbook, Qiagen, Venlo—Netherlands). After extraction, the DNA samples were visualized on a 1% agarose gel stained with GelRed (Biotium, California, USA). The 100-bp DNA standard from Invitrogen (California, USA) was used for the analysis of the genomic DNA. Eighteen samples did not present the total DNA band on the agarose gel and, consequently, did not yield any amplicon in the subsequent PCR reaction. These samples could not be analyzed as the correct species or a substitutions, leaving the final dataset with a total of 239 samples.

### PCR and sequencing

DNA amplification was carried out using primers selected from the Royal Botanic Gardens Kew Phase 2 Protocols and Update on Plant DNA Barcoding as follows: for *matK*, forward 5’—ACCCAGTCCATCTGGAAATCTTGGTTC—3’ (primer 1R_KIM-f) and reverse 5’—CGTACAGTACTTTTGTGTTTACGAG—3’ (primer 3F_KIM-r); for *rbcL*, forward 5’—ATGTCACCACAAACAGAGACTAAAGC—3’ (primer rbcLa_f) and reverse 5’—GAAACGGTCTCTCCAACGCAT—3’ (primer rbcLa_jf634R); and for ITS2, forward 5'—ATGCGATACTTGGTGTGAAT—3' (primer ITS-S2F) and reverse 5'—GACGCTTCTCCAGACTACAAT—3' (primer ITS3R) (http://www.kew.org/barcoding/protocols.html).

The PCR reactions were performed using a final volume of 25 μL containing 2 U of AmpliTaq Gold polymerase in GeneAmp 106 PCR Buffer II (100 mM Tris-HCl, pH 8.3; 500 mM KCl), 1.5 mM MgCl_2_ (Applied Biosystems, Foster City, CA), 0.2 mM dNTPs, 0.6 μM of each primer, and 100 to 200 ng of DNA.

The amplification was carried out in a GeneAmp PCR System 9700 Thermocycler (Applied Biosystems, Foster City, CA) using the following conditions: *matk—*an initial denaturation step at 98°C for 2 minutes; followed by 40 cycles at 98°C for 10 seconds, 52°C for 30 seconds and 72°C for 40 seconds; with a final extension period at 72°C for 10 minutes; *rbcL—*an initial denaturation step at 95°C for 3 minutes; followed by 45 cycles at 94°C for 30 seconds, 50°C for 40 seconds and 72°C for 40 seconds; with a final extension period at 72°C for 5 minutes; ITS2—an initial denaturation step at 95°C for 5 minutes; followed by 40 cycles at 94°C for 30 seconds, 56°C for 30 seconds and 72°C for 45 seconds; with a final extension period at 72°C for 10 minutes. After amplification, the DNA samples were visualized on a 1% agarose gel stained with GelRed (Biotium, California, USA). Six samples did not yield any amplicon in the subsequent PCR reaction. These samples could not be analyzed as the correct species or a substitutions, leaving the final dataset with a total of 233 samples.

The sequencing reactions used 2 μL containing 10 pmol of the same amplification reaction primers. Bi-directional sequencing was performed by Myleus Biotechnology (Belo Horizonte, Brazil) using an ABI3130 automated sequencer (Applied Biosystems, Foster City, CA) with BigDye v3.1.

### Data analysis

The obtained DNA sequences were edited using the SeqScape v2.7 software program (Applied Biosystems, Foster City, CA). Bases with a QV lower than 15 (i.e., a probability of error of 3.2%) were manually edited, and samples for which the entire sequence (or the majority of it) had a lower QV were discarded due to the high probability of error and/or impossibility of analyses [45]. Samples that amplified high-quality sequences from any one of the three genes (*rbcL*, *matK* or *ITS2*) were included in the analyses. The sequences produced in this work were submitted to GenBank (accession numbers KJ750965 through KJ751173 for *matK* sequences, KJ751175 through KJ751402 for *rbcL* sequences and KM519459 through KM519583 for *ITS2* sequences). Some of the sequences for *ITS2* (61 sequences/32,97% of the total ITS-2 dataset) had fewer than 200 base pairs and could therefore not be deposited in GenBank. Those sequences are available as supporting information (S1 File).

The reference sequences used to identify the generated sequences were mined from the Barcode of Life Data Systems (BOLD) (http://v3.boldsystems.org/index.php/databases) for the *matK* and *rbcL* regions and from GenBank (http://www.ncbi.nlm.nih.gov/genbank/) for the ITS2 region. BOLD archives are today the more reliable databases regarding DNA barcodes for reference species, since the criteria for a researcher to deposit a sequence is carefully reviewed and the specimen must be taxonomically identified by an expert. Some of the criteria include the deposit of at least five specimens vouchers of the reference species, the personal information of the botanist that made the identification of the specimens and several metadata that brings more security as to the correct identification. Since the official DNA barcode regions chosen for plant are *matk* and *rbcL*, GenBank had to be used for the ITS2 region, but when possible the ITS2 region was mined from BOLD. The reference sequences included every species from the eight genera analyzed in this study. Every query sequence that did not group with one of these genera was submitted to a Plant Identification via BOLD and to a MEGABLAST search on GenBank; the genera returned from these identifications were added to the phylogenetic analyses. The phylogenetic analyses and tree assembly were performed using the neighbor-joining (K2P) statistical method [41] in MEGA 5.2.2 [46]. The query sequences were identified according to the reference sequences with which they formed a cluster with a 98% similarity cutoff. Samples that grouped with a genus other than the eight target genera were promptly classified as substitutions.

To better identify the samples grouped within the 8-genus set of this study, the barcode gap approach was used [34]. For the barcode gap, the pairwise distance for each of the 8 genera was calculated individually using MEGA 5.2.2. The result was then exported to PAST [47], and a frequency histogram was assembled. The barcode gap was calculated for each of the three genes individually, for *matK* and *rbcL* together, and for *matK*, *rbcL* and *ITS2* together. For each calculation, a barcode gap was considered to exist if the frequency histogram showed a clear distinction between the intra- and interspecific genetic variation. When this distinction was unclear, no barcode gap was said to exist. Samples that presented a genetic variation higher than the maximum intraspecific variation were considered to be substitutions, and samples that presented genetic variation lower than the maximum intraspecific variation could not be identified as either a substitution or the correct species.

### Chemical analysis

The objective of these analyses was to verify the presence of chemical markers using thin layer chromatography (TLC) with silica gel plates (Merck Darmstadt, Ref 1.05.721). After TLC analysis, the concentration of the substances was determined using high-performance liquid chromatography (HPLC) or ultra-violet (UV) spectroscopy. The latter was performed only for a subset of the samples to demonstrate that the correctly identified samples may not have the minimum required concentration of the target chemical compounds and that samples identified as substitutions may have the chemical compounds at the minimum required concentration. Because each species has its own approved method for certification, the chemical analyses and the results interpretation followed the methods described on the American (*P*. *ginseng*), Brazilian (*M*. *ilicifolia* and *M*. *glomerata*) and British Pharmacopoeias (*H*. *virginiana*, *M*. *recutita*, *P*. *incarnata*, *P*. *boldus and V*. *officinalis*). Those methods are briefly detailed in Table 2.

**Table 2:** Conditions used for the chemical analyses.

Species	TLC	TLC	HPLC	UV
	Mobile phase	Developer solution	Mobile phase	Diluent and absorbancy
*H*. *virginiana*	Formic acid anidrous: water: ethyl acetate (10:10:80)	Ferric chloride	-	Phosphomolybdotungistc 760 nm
*M*. *recutita*	Ethyl acetate: toluene (5:95)	Anisaldehyde	Phase A (Phosphoric acid: water) 0,5:99,5 Phase B (Phosphoric acid: acetonitrile) 0,5:99,5	-
*M*. *ilicifolia*	Ethyl acetate: Formic acid: water (90:5:5)	Vanillin sulfuric	Phase A (water: trifluoroacetic acid 0,05%) Phase B (Acetonitrile: trifluoroacetic acid 0,05%)	-
*M*. *glomerata*	Toluene: dicholomethane: acetone (45:25:30)	Ethanolic KOH (50%)	Methanol: water (47:53)	-
*P*. *ginseng*	Butyl alcohol: ethyl acetate: water (10:2,5:5)	Anisaldehyde in glacial acetic acid plus methanol	Phase A (water) Phase B (Acetonitrile: water) 4:1	-
*P*. *incarnata*	Anhydrous formic acid: water: methyl ethyl ketone: ethyl acetate (10:10:30:50)	Diphenyl boric acid amino ethyl ester in methanol plus macrogol in methanol	-	Methanol (10 volumes): Glacial acetic acid (100 volumes): Boric acid 25 g/L (10 mL): oxalic acid (20 g/L) in formic acid anidrous 401nm
*P*. *boldus*	Diethylamine: metanol: toluene (10:10:80)	Potassium iodobismutate	Phase A (0,2 mL Diethylamine: 99,8 mL acetonitrile) Phase B (0,2 mL Diethylamine: 99,8 mL water)	-
*V*. *officinalis*	Glacial acetic acid: ethyl acetate: cyclohexane (2:38:60)	Anisaldehyde	Phase A (Acetonitrile R1 + Phosphoric acid solution 5 g/L) 20:80 Phase B (Phosphoric acid solution 5 g/L + Acetonitrile R1) 20:80	-

## Results

### DNA barcoding efficiency

**Fig 1:**
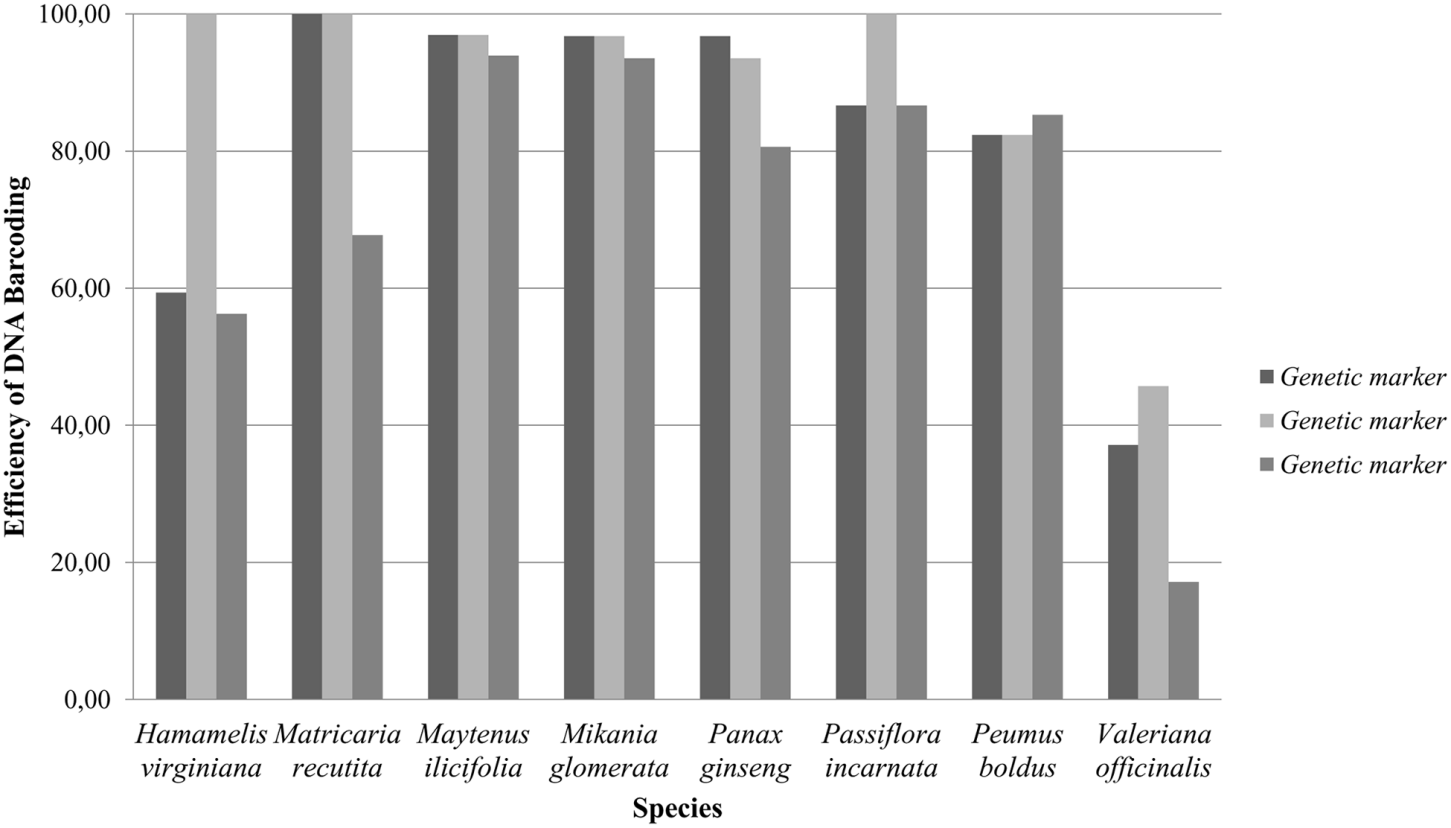
Percentage of samples analyzed according to species and genetic marker.

Among the 257 samples used in this study, the protocols for DNA extraction, PCR and sequencing worked for 209 (81.32%), 228 (88.72%) and 185 (71.98%) of the samples for the markers *matK*, *rbcL* and *ITS2*, respectively. These proportions varied greatly among the various species, with *M*. *ilicifolia* and *M*. *glomerata* yielding the best results and *V*. *officinalis* generating the worst ones (Fig 1). With the exception of *P*. *boldus*, the *ITS2* marker had the fewest samples that passed through the steps of DNA extraction, PCR and sequencing, whereas the *rbcL* region had the most samples passing through these steps (Fig 1).

In particular, the DNA barcoding protocol did not work properly for herbal medicines acquired from drugstores (*M*. *glomerata* sample 16, *P*. *ginseng* sample 07, *P*. *incarnata* sample 08, and *P*. *boldus* samples 14 and 17 through 20), with the exception of sample 08 from *P*. *incarnata*.

Samples that failed during the DNA barcoding protocol during the DNA extraction step, amplification step, or sequencing step were labeled as “No sequence” and were not considered in further analyses.

### Molecular markers efficacy

**Fig 2:**
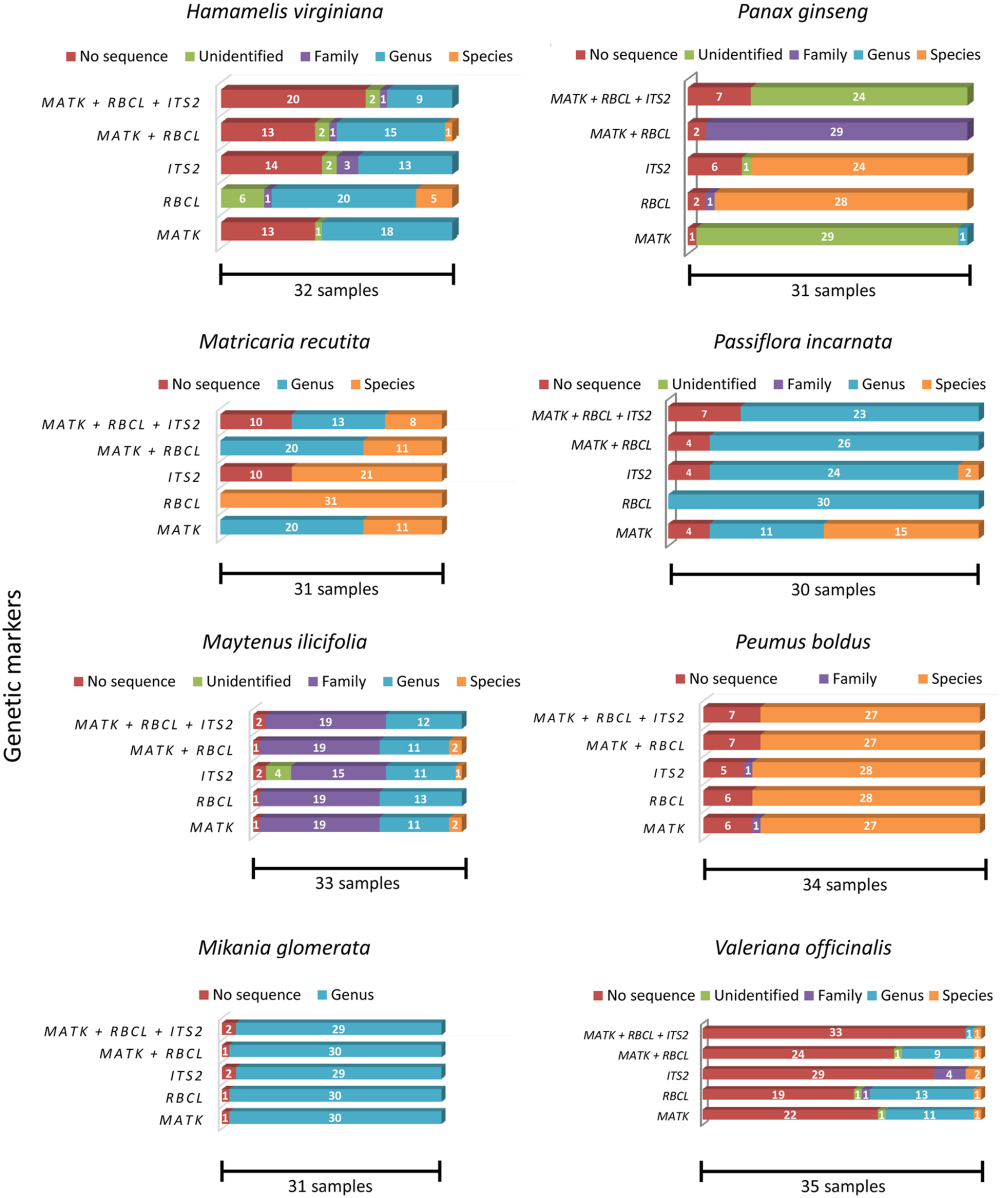
Identification levels for the analyzed samples when using each or a combination of the chosen markers. No sequence: samples for which the DNA barcoding protocol did not work. Unidentified: samples that could not be identified. The sequences from these samples did not show similarity levels above 98% to any of the sequences within the databases. Family: samples that could be identified at the family level. The sequences from these samples showed equal similarity levels to database sequences from multiple species belonging to the same family. Genus: samples that could be identified to the genus level. The sequences from these samples showed equal similarity levels to database sequences from multiple species belonging to the same genus. Species: samples that could be identified to the species level. The sequences from these samples showed similarity levels above 98% to database sequences from a unique species.

The *matK*, *rbcL*, and *ITS2* markers and their combinations achieved various levels of identification success for each of the eight medicinal species studied here (Fig 2). In many cases, identification at the species level was not possible for the species assayed in this work and with the markers used, considering the current amount of species reference sequences (DNA barcodes vouchers) deposited at BOLD and GenBank (S1 Table) because the genetic diversity within the genus was not sufficient to correctly identify a given sample at the species level. Because most of the substitutions found here involved species from different genera or even families, this result did not negatively impact the substitution analyses of this study. When samples were grouped within one of the eight medicinal genera, a barcode gap analysis was applied (Table 3). In some of these cases, it was possible to reach a final conclusion regarding the species identification, e.g., samples from *Matricaria recutita*. However, in other cases, the identification remained inconclusive, again because the genetic variation within the genus was not high enough (lower than 1%), even after applying the barcode gap.

**Table 3:** Barcode gap analyses.

SPECIES	MAXIMUM INTRASEPECIFIC DIVERGENCE				
	*matK*	*rbcL*	*ITS2*	*matK + rbcL*	*matK + rbcL + ITS2*
*Hamamelis virginiana*	0,026	X	0,019	0,026	0,033
*Matricaria recutita*	0,00042	0,00068	0,0058	0,00024	0,0014
*Maytenus ilicifolia*	X	0,016	X	0,028	X
*Mikania glomerata*	0,0017	0,0005	0,02	0,0005	0,0058
*Panax ginseng*	0,024	0,021	X	0,028	0,046
*Passiflora incarnata*	X	X	X	0,035	0,038
*Peumus boldus*	Not applicable*				
*Valeriana officinalis*	X	X	0,08	X	0,046

The numbers represent the maximum intra-specific divergence. Values above this number were considered as a different species.

X—The Barcode Gap was not calculated because there was no clear division between intra- and interspecific genetic divergence.

*The genus *Peumus* possess only one specie, which makes the Barcode Gap not applicable.

### Molecular identification and species substitution

The phylogenetic analyses applied to the sequences retrieved from the DNA barcoding methodology revealed that all 8 analyzed medicinal species, with the exception of *M*. *glomerata*, had samples that were substituted with other species, genera or even other families (S1–S40 Figs).

**Fig 3:**
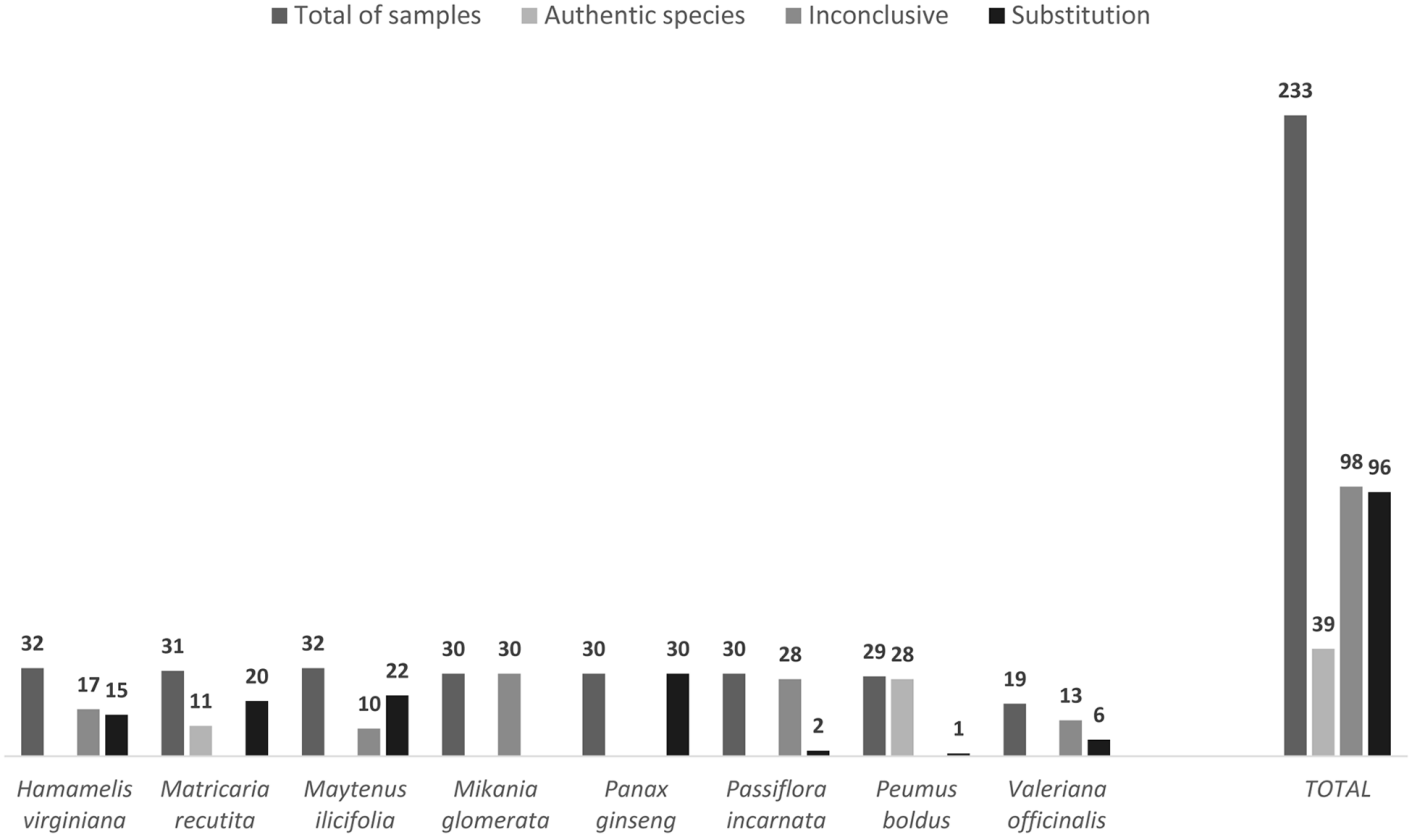
DNA final barcode identification of the analyzed samples.

From the samples that passed through the DNA barcoding protocol, 42.06% belonged to the expected genus but could not be identified to the species level; these samples were therefore classified as “inconclusive” in terms of substitutions. The remaining samples were classified as either substitute (71.11%) or authentic (28.89%), depending on the concordance between the expected and observed species (Fig 3). The proportion of samples classified as substitutions varied greatly among the eight species. For example, 100% of the samples presented as *P*. *ginseng* were actually from the genus *Pfaffia*, a Brazilian ginseng, whereas only 3.45% of the samples presented as *P*. *boldus* were substitutions (Fig 3).

For *H*. *virginiana*, half of the samples (16) belonged to the genus *Hamamelis* (Hamamelidaceae), and one sample belonged to the same family but was from a genus that could not be defined. Five samples could not be identified, and the remaining ten samples were distributed among another seven different families. It is interesting to note the presence of samples identified as Brazilian native species, such as *Solanum* and *Lantana*, as well as the presence of other species that are also imported to Brazil, such as *Tilia*.

All of the samples from *M*. *recutita* (Asteraceae) corresponded to the correct genus, but twenty samples presented a certain level of genetic diversity for the marker *matK* (S26 Fig). When the barcode gap analysis was applied, these samples were assigned to a species other than *M*. *recutita*. Despite these observations, those samples were not linked to any other species and their genetic diversity was found to be extremely low (lower than 0,01%).

Although some of the samples labeled as *M*. *ilicifolia* (Celastraceae) were found to belong to the genus *Maytenus*, the majority were identified at the family level (Fabaceae) as one of two species, *Zollernia ilicifolia* or *Lecointea peruviana*, and one sample was identified as the genus *Roupala* (Proteaceae), which includes species that are native to Brazil but morphologically distinct from *M*. *ilicifolia* and with no previous reports of use in folk medicine (S1 Table).

Neither of the sequences for *M*. *glomerata* (Asteraceae) was successful as a tool able to identify substitution because it was impossible to distinguish between *M*. *glomerata* and *M*. *laevigata*.

In the case of *P*. *ginseng*, a species that originated in Asia and was imported to Brazil, most of the samples were identified as *Pfaffia spp*. (Amaranthaceae). This genus contains the species *Pfaffia glomerata*, a plant that is native to Brazil and popularly known as Brazilian ginseng. The only exception for this group was one sample that was identified only at the family level (Amaranthaceae) but could not be distinguished among the genera *Pfaffia*, *Hebanthe* and *Pseudoplantago*.

In the analyses of *P*. *incarnata* (Passifloraceae), two clear substitutions were found of the species *Senna alexandrina* (Fabaceae). All other samples belonged to the genus *Passiflora*.

Most of the samples of *Peumus* (Monimiaceae), a genus with only one species (*P*. *boldus*, http://www.theplantlist.org/browse/A/Monimiaceae/Peumus/), were identified as the correct species. One exception was identified as *Vernonia colorata* (Asteraceae) (S1 Table).

For *V*. *officinalis*, the whole process of DNA extraction, amplification and sequencing did not work well and the sequences obtained were mostly low quality. From thirty-five samples, only nineteen (54,28%) could be analyzed using DNA barcoding. Of these, thirteen belonged to the genus *Valeriana* but could not be identified at the species level. Two samples that were identified only at the family level belonged to Asteraceae. One sample was identified as belonging to a different genus (*Cissampelos*). Two other samples were identified as different species: *Ageratum conyzoides* and *Stellaria vestita*. One sample could not be identified.

### Chemical analysis

**Fig 4:**
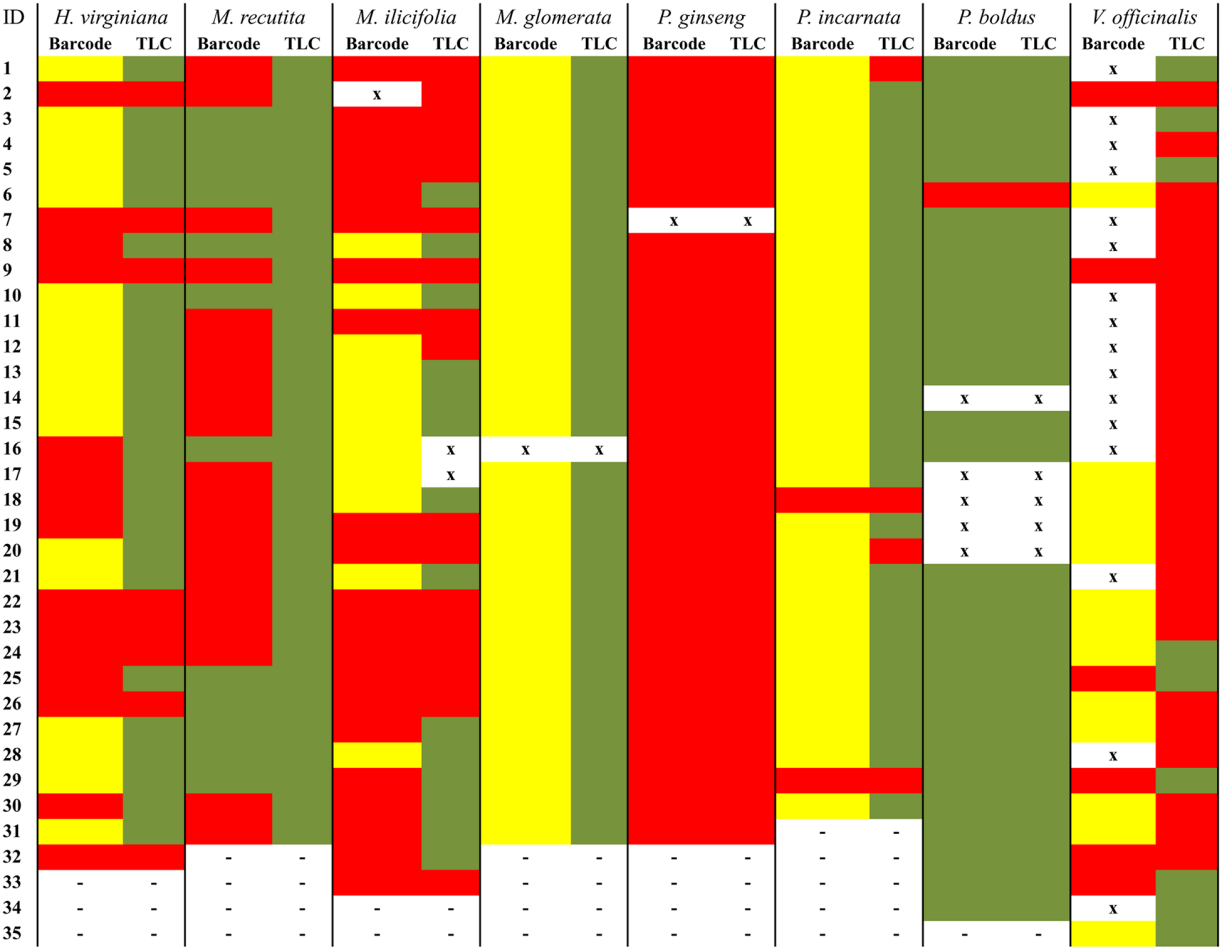
Comparison between the DNA barcode and TLC findings. ID: sample number. Green: samples that were identified as the expected medicinal species using DNA barcoding and that contained the expected chemical marker from the medicinal species according to TLC. Yellow: samples that were not identified within the genus of the medicinal species using DNA barcoding. Red: samples that were identified using DNA barcoding as a genus or family that varied from the expected one and that did not contain the chemical marker according to TLC. X: samples that did not generate any sequence using DNA barcoding or that could not be tested using TLC. -: absent samples.

For most of the studied species, TLC, HPLC and UV analyses confirmed the molecular findings for samples identified as not being the true plant; many samples did not contain the expected chemical marker for the labeled medicinal species. In some cases (*H*. *virginiana*, *M*. *Recutita*, *M*. *ilicifolia* and *V*. *officinalis*), some substitutions showed a chromatography pattern resembling that of the correct species. In these cases, only molecular analysis made the correct identification possible. For *P*. *ginseng*, all samples were negative for the expected chemical marker. However, all samples labeled as *M*. *recutita* and *M*. *glomerata* contained the expected chemical marker (Fig 4).

The simple presence of the chemical markers is not sufficient to validate an herbal medicine preparation, but it is mandatory that a minimal concentration of the chemical marker is present. As expected, the samples that showed negative results via TLC also showed negative results via HPLC or UV. However, for some samples that were positive via TLC, the chemical marker was not present at the minimum concentration required for validation. This finding was true for samples from *M*. *recutita*, *M*. *glomerata P*. *incarnata* and *V*. *officinalis* (Table 4).

**Table 4:** Molecular identification versus TLC, HPLC and UV analyses.

Species, minimal [] of chemical markers and method of dosage	Samples	Molecular identification	Chemical TLC	Markers Content
*H*. *virginiana* (3,0% of tannins/ UV)	01	*Hamamelis spp*.	Present	3,59%
	02	*Betula spp*.	Not Present	0,57%
	07	*Tilia spp*.	Not Present	0,23%
	08	*Solanum spp*.	Present	4,22%
	09	Unidentified	Not Present	0,61%
	12	*Hamamelis spp*.	Present	4,04%
	17	*Lantana spp*.	Present	4,43%
	22	*Persicaria spp*.	Not Present	0,84%
	23	*Sterculia urens*	Not Present	0,20%
	28	*Hamamelis spp*.	Present	4,13%
*M*. *recutita* (0,25% Apigenin 7-glucoside/ HPLC)	05	*Matricaria spp*.	Present	0,005%
	20	*Matricaria spp*.	Present	0,001%
	24	*Matricaria spp*.	Present	0,001%
	27	*Matricaria recutita*	Present	0,002%
*M*. *ilicifolia* (2,8 mg/g of Epicatechin, HPLC)	06	*Roupala spp*.	Present	26,32 mg/g
	10	*Maytenus spp*.	Present	79,80 mg/g
	22	*Sorocea affinis*	Not Present	-
	24	Fabaceae	Not Present	-
	28	*Maytenus spp*.	Present	107,44 mg/g
*M*. *glomerata* (0,1% of Cumarin, HPLC)	04	*Mikania spp*.	Present	0,038%
	15	*Mikania spp*.	Present	0,020%
	26	*Mikania spp*.	Present	0,011%
*P*. *ginseng* (0,2% ginsenoside Rg_1_ and 0,1% ginsenoside Rb_1_, HPLC)	05	*Pfaffia dunaliana*	Not Present	-
	08	*Pfaffia dunaliana*	Not Present	-
	10	Amaranthaceae	Not Present	-
	13	*Pfaffia dunaliana*	Not Present	-
	25	*Pfaffia dunaliana*	Not Present	-
*P*. *incarnata* (1,5% of total flavonoids, UV)	01	*Passiflora spp*.	Not Present	0,126%
	03	*Passiflora spp*.	Present	1,614%
	08	*Passiflora spp*.	Present	1,58%
	13	*Passiflora spp*.	Present	0,833%
	18	*Senna alexandrina*	Not Present	0,154%
	21	*Passiflora spp*.	Present	0,97%
	29	*Senna alexandrina*	Not Present	0,229%
*P*. *boldus* (0,1% of total alkaloids, HPLC)	06	*Vernonia colorata*	Not Present	-
	08	*Peumus boldus*	Present	0,33%
	26	*Peumus boldus*	Present	0,72%
	31	*Peumus boldus*	Present	0,29%
*V*. *officinalis* (0,05% of valerenic acid, HPLC)	02	*Ageratum conyzoides*	Not present	-
	04	No sequence	Not present	-
	06	*Valeriana spp*.	Not present	-
	09	Unidentified	Not present	-
	22	*Valeriana spp*.	Not present	-
	24	*Valeriana spp*.	Present	0,037%
	25	*Asteraceae*	Present	0,023%
	29	*Cissampelos spp*.	Present	0,050%
	32	*Valeriana spp*.	Not present	-

### Molecular and chemical correlation

In some cases, samples that were identified as substitutions using molecular analysis actually did contain the expected chemical marker from the labeled species. That was the case for samples from *H*. *virginiana*, *M*. *recutita* and *M*. *ilicifolia* (Fig 4). On the other hand, every sample that matched the labeled species according to molecular identification was also positive on the TLC analyses (Fig 4).

During the final step of concentration analyses, HPLC or UV, two interesting points arose. First, the presence of the correct chemical marker(s) in a sample does not mean that the sample contained the minimum concentration required. This result was observed for samples of *M*. *recutita*, *M*. *glomerata* and *P*. *incarnata* (Table 4). Second, some samples that were identified as substitutions using DNA barcoding but contained the expected chemical marker from the medicinal species also presented the minimum concentration required for validation on HPLC or UV (Table 4). This result was observed for samples from *H*. *virginiana*, *M*. *ilicifolia* and *V*. *officinalis*.

Overall, *V*. *officinalis* was the most difficult species to work with during these analyses. The medicinal part of *V*. *officinalis* plants is the roots. *V*. *officinalis* root cells contain a light brown resin [10] that was most likely responsible for the unsatisfactory results of the genetic analyses because it completely inhibited the PCR or generated problems during the amplification process. Modifications made to the protocols to attempt to resolve this problem were not effective. Only nine samples were positive according to TLC, and of the samples submitted to HPLC, only one (sample 29) met the minimum required concentration. Curiously, this sample was identified from DNA barcoding as belonging to the *Cissampelos* genus. (Table 4).

## Discussion

Plants used to prepare herbal medicines are marketed as crude drugs, and the quality of these materials is currently verified by a set of botanical, physicochemical and chemical analyses that have been established by Pharmacopoeias and other official compendia [48]. Those methods, however, are not completely reliable for species identification, and several studies have revealed species substitutions [19, 22, 31, 49].

DNA-based methods, such as the use of specific DNA sequences as markers for species identification, are used in a range of field, including agriculture [50–52] and zootechny [53–55], and comprise various methods, such as RAPF, AFLP, PCR-DGGE, real-time PCR and sequencing-based systems, such as SSR [50, 56–59]. Choosing the most appropriate method depends on several factors, including the focus of the study [60]. However, the availability of a variety of methods and approaches can also hamper research; the lack of standardization and universality decreases the reproducibility of studies. However, the proposed goal of the DNA barcode project [34] to catalogue universal markers for all life on Earth has the potential to unify DNA-based methods used for species identification.

Using common sets of primers, databases and standards to catalogue species by research groups all around the world increases the level of reliability and the number of species available for study (which has reached the greatest level ever achieved by the scientific community) while also making it possible to identify an ever-growing number of species. The definition of an official DNA barcode for plants was a crucial step, and the sequences chosen have already proven themselves to be of great value [9, 60, 61]. The discovery of universal primers for the DNA barcode would be the perfect scenario, but this goal may not be achieved. Small nuances in different families, orders and species are responsible for different levels of amplification and in some cases the use of different primers might be the best strategy to follow to make the amplification and sequencing more efficient.

Processed samples, such as the ones analyzed on this study, are often hard-working, since the isolation of good-quality DNA may be difficult to achieve [62]. Even though we were able to analyze the majority of the samples (233 from 257) using at least one of the three markers, better ways to work with processed samples are becoming available and will be applied in future studies [43]. An example is the DNA mini-Barcode, based on the analyses of smaller regions. A DNA mini-barcode for *rbcL* is already available [63].

This study demonstrated that it is not always necessary to work with both sequences *matK* and *rbcL* when the purpose of the study is not to catalogue new species but rather to identify species from a collection of samples; this study also demonstrated that the DNA barcode approach has limitations. For all of the samples, the use of the *rbcL* and *matk* sequences together only improved species identification in two cases, one for *Hamamelis* (sample 16) and one for *Peumus* (sample 6); in both of these cases, the samples could be identified to the species level only when the two markers were used together (S4 and S34 Figs).

The use of the DNA barcoding technology enabled us to detect several substitutions among the analyzed samples. Most substitutions involved species from different genera (or even a different family) than those of the expected medicinal species. When analyzing multiple species within the same genus, *matK* and *rbcL* were only rarely able to correctly identify the samples. That was the case for samples belonging to some of the analyzed species. For example, the markers could not distinguish between *M*. *glomerata* and *M*. *laevigata*. Both species are used in folk medicine in Brazil and have the same geographical distribution and several morphological and chemical similarities. For these reasons, it is believed that *M*. *laevigata*, which is not included in the ANVISA list of approved species for herbal medicines, is frequently used as a substitute for *M*. *glomerata* [64]. For *M*. *ilicifolia*, most of the samples belonged to *Zollernia ilicifolia* or *Lecointea peruviana*. These species share similar morphology and like *M*. *ilicifolia*, belong to the clade Lecointea, together with the closely related genera *Exostyles*, *Harleyodendron* and *Holocalyx* [65, 66]. Most of the *P*. *incarnata* samples belonged to the genus *Passiflora* but could not be identified at the species level. The *matK* region showed promising results for differentiating species within the genus *Passiflora* (S26 Fig), but our analysis was ultimately unsuccessful because none of the databases contained this sequence for *P*. *incarnata*. Brazil is one of the greatest producers of *Passiflora* species for food [67], and it is likely that some of that production ends up being marketed as herbal medicine.

The difficulty in differentiating closely related species is supported by the fact that the methodologies used to perform distance-based species discriminations based on DNA barcodes are still being worked out [68, 69]. Furthermore, the difficulty in identifying closely related species is especially pronounced in plants [70]. For this reason, the barcode sequences were only recently defined, and the search for better loci continues [43, 69]. In this study, we attempted to use the ITS2 region to improve the accuracy of species identification. However, our attempt was not successful, primarily due to difficulties encountered in working with the sequence and the fact that it did not add additional variability compared with analysis based on *matK* and *rbcL*.

Some of the substitutions that we identified, such as the genera *Solanum* and *Lantana* for *H*. *virginiana* or the genera *Ageratum* and *Cissampelos* for *V*. *officinalis*, are most likely a consequence of the ease of obtaining samples of the substitutes, which are native to Brazil. In fact, Hamamelis is native to North America, and *V*. *officinalis* is native to Europe; it is necessary to import both plants for use in Brazil. The same is also true for *P*. *ginseng*, but in this specific case, a mistake may have occurred because the Brazilian ginseng (*Pfaffia glomerata*) and the Asian ginseng (*P*. *ginseng*) are both known as ginseng. Another case of substitution due to popular name confusion may have occurred when the genus *Sorocea* (Moraceae), to which the species *Sorocea bonplandii* belongs, was used as a substituted for *M*. *ilicifolia*; *Sorocea bonplandii* has the same popular name as *M*. *ilicifolia* in Brazil (Espinheira santa) and a similar morphology [65]. Finally, a similar explanation might be responsible for the only substitution found for *P*. *boldus*. The genus *Vernonia* contains the species *V*. *condensata*, which is known in Brazil as “Boldo baiano” and regularly used as a substitute for *P*. *boldus*, despite their complete lack of similarity [71].

Curiously, we also detected *Tilia* among the samples of *Hammamelis*. This plant does not occur in Brazil, and its presence in the market here indicates that substitutions are sometimes occurring outside Brazil, which may also be the case for the sample of *S*. *alexandrina* that was found among the samples sold as *P*. *incarnata*. This species is popularly used in certain countries (including Brazil) for constipation, but recent studies have revealed toxic effects in mouse models [72, 73].

The parallels between the genetic and chemical analyses proved that it is possible for a sample to pass quality control tests even if it does not belong to the correct species. This result was observed for samples that were identified as substitutions using DNA barcoding but exhibited similarity with the correct species according to TLC and contained concentrations of chemical markers that were above the required minimums (*H*. *virginiana* samples 08 and 17, *M*. *ilicifolia* sample 06, and *V*. *officinalis* sample 29). These results may be attributed to the specificity of the chemical markers; even though some of these chemicals substances used as markers, such as valerenic acid, are very specific, others (such as tannins) are common to a large variety of plants. However, these analyses also demonstrated that correct species identification is not sufficient because the active compound may not be present in the samples or may be below the minimum required concentration. Thus, when taking into account the results of DNA barcoding, TLC and HPLC or UV, the complementarity of the tests becomes clear.

In addition to the health implications of the correct use of the approved medicinal species, another factor that should be considered is the possible environmental impacts of these plants. It is estimated that one in every five vegetal species in the world is threatened. It has been suggested that the herbal market poses a threat to biodiversity through the over-harvesting of raw materials [31, 74–76]. In a previous study, our group demonstrated positive results regarding the inhibition of native species collection in the wild by pharmaceutical companies following the establishment of rules from the Brazilian Health Ministry [77]. The impact of the use of native materials sold in popular markets, however, is difficult to estimate because these materials are obtained from various suppliers and from unmanaged forests. If the findings of this study are cross-referenced with the Official List of Endangered Species of the Brazilian Flora [78], the genera *Solanum* (one species), *Maytenus* (four species), *Mikania* (six species), *Pfaffia* (three species), *Passiflora* (five species), and *Vernonia* (fifteen species) are all represented, demonstrating that correct species identification is required to prevent the use of threatened species.

## Conclusions

The present study showed a great number of species substitutions and mislabeling, demonstrating that the current surveillance methods are not being efficient to control he herbal medicine market. Also, we showed that the traditional methodologies of species identification using chemical analysis are, in the majority of cases, not adequate to correctly identify a plant species. Thus, we propose the use of DNA barcode as a powerful first screening step. Applying the DNA barcode technique to the quality control of herbal medicine production will make the process safer, more reliable, and cheaper because substitutions will be promptly discarded without requiring more expensive chemical analyses that are otherwise necessary.

## Supporting Information

S1 Fig Phylogenetic tree *Hammamelis virginiana matK*. The evolutionary history was inferred using the Neighbor-Joining method. The optimal tree with the sum of branch length = 1.47656508 is shown. The percentage of replicate trees in which the associated taxa clustered together in the bootstrap test (500 replicates) are shown next to the branches. The tree is drawn to scale, with branch lengths in the same units as those of the evolutionary distances used to infer the phylogenetic tree. The evolutionary distances were computed using the Maximum Composite Likelihood method and are in the units of the number of base substitutions per site. The analysis involved 442 nucleotide sequences. Codon positions included were 1st+2nd+3rd+Noncoding. All positions containing gaps and missing data were eliminated. There were a total of 205 positions in the final dataset. Evolutionary analyses were conducted in MEGA5.
(PDF)

S2 Fig Phylogenetic tree *Hammamelis virginiana rbcL*. The evolutionary history was inferred using the Neighbor-Joining method. The optimal tree with the sum of branch length = 0.67568534 is shown. The percentage of replicate trees in which the associated taxa clustered together in the bootstrap test (500 replicates) are shown next to the branches. The tree is drawn to scale, with branch lengths in the same units as those of the evolutionary distances used to infer the phylogenetic tree. The evolutionary distances were computed using the Maximum Composite Likelihood method and are in the units of the number of base substitutions per site. The analysis involved 370 nucleotide sequences. Codon positions included were 1st+2nd+3rd+Noncoding. All positions containing gaps and missing data were eliminated. There were a total of 215 positions in the final dataset. Evolutionary analyses were conducted in MEGA5.
(PDF)

S3 Fig Phylogenetic tree *Hammamelis virginiana ITS2*. The evolutionary history was inferred using the Neighbor-Joining method. The optimal tree with the sum of branch length = 0.71702123 is shown. The percentage of replicate trees in which the associated taxa clustered together in the bootstrap test (500 replicates) are shown next to the branches. The tree is drawn to scale, with branch lengths in the same units as those of the evolutionary distances used to infer the phylogenetic tree. The evolutionary distances were computed using the Maximum Composite Likelihood method and are in the units of the number of base substitutions per site. The analysis involved 207 nucleotide sequences. Codon positions included were 1st+2nd+3rd+Noncoding. All positions containing gaps and missing data were eliminated. There were a total of 13 positions in the final dataset. Evolutionary analyses were conducted in MEGA5.
(PDF)

S4 Fig Phylogenetic tree *Hammamelis virginiana matK + rbcL*. The evolutionary history was inferred using the Neighbor-Joining method. The optimal tree with the sum of branch length = 0.86650001 is shown. The percentage of replicate trees in which the associated taxa clustered together in the bootstrap test (500 replicates) are shown next to the branches. The tree is drawn to scale, with branch lengths in the same units as those of the evolutionary distances used to infer the phylogenetic tree. The evolutionary distances were computed using the Maximum Composite Likelihood method and are in the units of the number of base substitutions per site. The analysis involved 189 nucleotide sequences. Codon positions included were 1st+2nd+3rd+Noncoding. All positions containing gaps and missing data were eliminated. There were a total of 620 positions in the final dataset. Evolutionary analyses were conducted in MEGA5.
(PDF)

S5 Fig Phylogenetic tree *Hammamelis virginiana matK + rbcL + ITS2*. The evolutionary history was inferred using the Neighbor-Joining method. The optimal tree with the sum of branch length = 0.51134035 is shown. The percentage of replicate trees in which the associated taxa clustered together in the bootstrap test (500 replicates) are shown next to the branches. The tree is drawn to scale, with branch lengths in the same units as those of the evolutionary distances used to infer the phylogenetic tree. The evolutionary distances were computed using the Maximum Composite Likelihood method and are in the units of the number of base substitutions per site. The analysis involved 54 nucleotide sequences. Codon positions included were 1st+2nd+3rd+Noncoding. All positions containing gaps and missing data were eliminated. There were a total of 726 positions in the final dataset. Evolutionary analyses were conducted in MEGA5.
(PDF)

S6 Fig Phylogenetic tree *Matricaria recutita matK*. The evolutionary history was inferred using the Neighbor-Joining method. The optimal tree with the sum of branch length = 0.00478244 is shown. The percentage of replicate trees in which the associated taxa clustered together in the bootstrap test (500 replicates) are shown next to the branches. The tree is drawn to scale, with branch lengths in the same units as those of the evolutionary distances used to infer the phylogenetic tree. The evolutionary distances were computed using the Maximum Composite Likelihood method and are in the units of the number of base substitutions per site. The analysis involved 36 nucleotide sequences. Codon positions included were 1st+2nd+3rd+Noncoding. All positions containing gaps and missing data were eliminated. There were a total of 631 positions in the final dataset. Evolutionary analyses were conducted in MEGA5.
(PDF)

S7 Fig Phylogenetic tree *Matricaria recutita rbcL*. The evolutionary history was inferred using the Neighbor-Joining method. The optimal tree with the sum of branch length = 0.00211645 is shown. The percentage of replicate trees in which the associated taxa clustered together in the bootstrap test (500 replicates) are shown next to the branches. The tree is drawn to scale, with branch lengths in the same units as those of the evolutionary distances used to infer the phylogenetic tree. The evolutionary distances were computed using the Maximum Composite Likelihood method and are in the units of the number of base substitutions per site. The analysis involved 36 nucleotide sequences. Codon positions included were 1st+2nd+3rd+Noncoding. All positions containing gaps and missing data were eliminated. There were a total of 473 positions in the final dataset. Evolutionary analyses were conducted in MEGA5.
(PDF)

S8 Fig Phylogenetic tree *Matricaria recutita ITS2*. The evolutionary history was inferred using the Neighbor-Joining method. The optimal tree with the sum of branch length = 0.12287265 is shown. The percentage of replicate trees in which the associated taxa clustered together in the bootstrap test (500 replicates) are shown next to the branches. The tree is drawn to scale, with branch lengths in the same units as those of the evolutionary distances used to infer the phylogenetic tree. The evolutionary distances were computed using the Maximum Composite Likelihood method and are in the units of the number of base substitutions per site. The analysis involved 27 nucleotide sequences. Codon positions included were 1st+2nd+3rd+Noncoding. All positions containing gaps and missing data were eliminated. There were a total of 191 positions in the final dataset. Evolutionary analyses were conducted in MEGA5.
(PDF)

S9 Fig Phylogenetic tree *Matricaria recutita matK + rbcL*. The evolutionary history was inferred using the Neighbor-Joining method. The optimal tree with the sum of branch length = 0.00363776 is shown. The percentage of replicate trees in which the associated taxa clustered together in the bootstrap test (500 replicates) are shown next to the branches. The tree is drawn to scale, with branch lengths in the same units as those of the evolutionary distances used to infer the phylogenetic tree. The evolutionary distances were computed using the Maximum Composite Likelihood method and are in the units of the number of base substitutions per site. The analysis involved 36 nucleotide sequences. Codon positions included were 1st+2nd+3rd+Noncoding. All positions containing gaps and missing data were eliminated. There were a total of 1104 positions in the final dataset. Evolutionary analyses were conducted in MEGA5.
(PDF)

S10 Fig Phylogenetic tree *Matricaria recutita matK + rbcL + ITS2*. The evolutionary history was inferred using the Neighbor-Joining method. The optimal tree with the sum of branch length = 0.00534820 is shown. The percentage of replicate trees in which the associated taxa clustered together in the bootstrap test (500 replicates) are shown next to the branches. The tree is drawn to scale, with branch lengths in the same units as those of the evolutionary distances used to infer the phylogenetic tree. The evolutionary distances were computed using the Maximum Composite Likelihood method and are in the units of the number of base substitutions per site. The analysis involved 24 nucleotide sequences. Codon positions included were 1st+2nd+3rd+Noncoding. All positions containing gaps and missing data were eliminated. There were a total of 1319 positions in the final dataset. Evolutionary analyses were conducted in MEGA5.
(PDF)

S11 Fig Phylogenetic tree *Maytenus ilicifolia matK*. The evolutionary history was inferred using the Neighbor-Joining method. The optimal tree with the sum of branch length = 0.39435507 is shown. The percentage of replicate trees in which the associated taxa clustered together in the bootstrap test (500 replicates) are shown next to the branches. The tree is drawn to scale, with branch lengths in the same units as those of the evolutionary distances used to infer the phylogenetic tree. The evolutionary distances were computed using the Maximum Composite Likelihood method and are in the units of the number of base substitutions per site. The analysis involved 112 nucleotide sequences. Codon positions included were 1st+2nd+3rd+Noncoding. All positions containing gaps and missing data were eliminated. There were a total of 98 positions in the final dataset. Evolutionary analyses were conducted in MEGA5.
(PDF)

S12 Fig Phylogenetic tree *Maytenus ilicifolia rbcL*. The evolutionary history was inferred using the Neighbor-Joining method. The optimal tree with the sum of branch length = 0.16105704 is shown. The percentage of replicate trees in which the associated taxa clustered together in the bootstrap test (500 replicates) are shown next to the branches. The tree is drawn to scale, with branch lengths in the same units as those of the evolutionary distances used to infer the phylogenetic tree. The evolutionary distances were computed using the Maximum Composite Likelihood method and are in the units of the number of base substitutions per site. The analysis involved 84 nucleotide sequences. Codon positions included were 1st+2nd+3rd+Noncoding. All positions containing gaps and missing data were eliminated. There were a total of 370 positions in the final dataset. Evolutionary analyses were conducted in MEGA5.
(PDF)

S13 Fig Phylogenetic tree *Maytenus ilicifolia ITS2*. The evolutionary history was inferred using the Neighbor-Joining method. The optimal tree with the sum of branch length = 1.85107254 is shown. The percentage of replicate trees in which the associated taxa clustered together in the bootstrap test (500 replicates) are shown next to the branches. The tree is drawn to scale, with branch lengths in the same units as those of the evolutionary distances used to infer the phylogenetic tree. The evolutionary distances were computed using the Maximum Composite Likelihood method and are in the units of the number of base substitutions per site. The analysis involved 77 nucleotide sequences. Codon positions included were 1st+2nd+3rd+Noncoding. All positions containing gaps and missing data were eliminated. There were a total of 100 positions in the final dataset. Evolutionary analyses were conducted in MEGA5.
(PDF)

S14 Fig Phylogenetic tree *Maytenus ilicifolia matK + rbcL*. The evolutionary history was inferred using the Neighbor-Joining method. The optimal tree with the sum of branch length = 0.22908763 is shown. The percentage of replicate trees in which the associated taxa clustered together in the bootstrap test (500 replicates) are shown next to the branches. The tree is drawn to scale, with branch lengths in the same units as those of the evolutionary distances used to infer the phylogenetic tree. The evolutionary distances were computed using the Maximum Composite Likelihood method and are in the units of the number of base substitutions per site. The analysis involved 75 nucleotide sequences. Codon positions included were 1st+2nd+3rd+Noncoding. All positions containing gaps and missing data were eliminated. There were a total of 518 positions in the final dataset. Evolutionary analyses were conducted in MEGA5.
(PDF)

S15 Fig Phylogenetic tree *Maytenus ilicifolia matK + rbcL + ITS2*. The evolutionary history was inferred using the Neighbor-Joining method. The optimal tree with the sum of branch length = 0.31908268 is shown. The percentage of replicate trees in which the associated taxa clustered together in the bootstrap test (500 replicates) are shown next to the branches. The tree is drawn to scale, with branch lengths in the same units as those of the evolutionary distances used to infer the phylogenetic tree. The evolutionary distances were computed using the Maximum Composite Likelihood method and are in the units of the number of base substitutions per site. The analysis involved 39 nucleotide sequences. Codon positions included were 1st+2nd+3rd+Noncoding. All positions containing gaps and missing data were eliminated. There were a total of 858 positions in the final dataset. Evolutionary analyses were conducted in MEGA5.
(PDF)

S16 Fig Phylogenetic tree *Mikania glomerata matK*. The evolutionary history was inferred using the Neighbor-Joining method. The optimal tree with the sum of branch length = 0.00738890 is shown. The percentage of replicate trees in which the associated taxa clustered together in the bootstrap test (500 replicates) are shown next to the branches. The tree is drawn to scale, with branch lengths in the same units as those of the evolutionary distances used to infer the phylogenetic tree. The evolutionary distances were computed using the Maximum Composite Likelihood method and are in the units of the number of base substitutions per site. The analysis involved 46 nucleotide sequences. Codon positions included were 1st+2nd+3rd+Noncoding. All positions containing gaps and missing data were eliminated. There were a total of 679 positions in the final dataset. Evolutionary analyses were conducted in MEGA5.
(PDF)

S17 Fig Phylogenetic tree *Mikania glomerata rbcL*. The evolutionary history was inferred using the Neighbor-Joining method. The optimal tree with the sum of branch length = 0.00755703 is shown. The percentage of replicate trees in which the associated taxa clustered together in the bootstrap test (500 replicates) are shown next to the branches. The tree is drawn to scale, with branch lengths in the same units as those of the evolutionary distances used to infer the phylogenetic tree. The evolutionary distances were computed using the Maximum Composite Likelihood method and are in the units of the number of base substitutions per site. The analysis involved 47 nucleotide sequences. Codon positions included were 1st+2nd+3rd+Noncoding. All positions containing gaps and missing data were eliminated. There were a total of 531 positions in the final dataset. Evolutionary analyses were conducted in MEGA5.
(PDF)

S18 Fig Phylogenetic tree *Mikania glomerata ITS2*. The evolutionary history was inferred using the Neighbor-Joining method. The optimal tree with the sum of branch length = 0.21453668 is shown. The percentage of replicate trees in which the associated taxa clustered together in the bootstrap test (500 replicates) are shown next to the branches. The tree is drawn to scale, with branch lengths in the same units as those of the evolutionary distances used to infer the phylogenetic tree. The evolutionary distances were computed using the Maximum Composite Likelihood method and are in the units of the number of base substitutions per site. The analysis involved 49 nucleotide sequences. Codon positions included were 1st+2nd+3rd+Noncoding. All positions containing gaps and missing data were eliminated. There were a total of 220 positions in the final dataset. Evolutionary analyses were conducted in MEGA5.
(PDF)

S19 Fig Phylogenetic tree *Mikania glomerata matK + rbcL*. The evolutionary history was inferred using the Neighbor-Joining method. The optimal tree with the sum of branch length = 0.00165371 is shown. The percentage of replicate trees in which the associated taxa clustered together in the bootstrap test (500 replicates) are shown next to the branches. The tree is drawn to scale, with branch lengths in the same units as those of the evolutionary distances used to infer the phylogenetic tree. The evolutionary distances were computed using the Maximum Composite Likelihood method and are in the units of the number of base substitutions per site. The analysis involved 45 nucleotide sequences. Codon positions included were 1st+2nd+3rd+Noncoding. All positions containing gaps and missing data were eliminated. There were a total of 1210 positions in the final dataset. Evolutionary analyses were conducted in MEGA5.
(PDF)

S20 Fig Phylogenetic tree *Mikania glomerata matK + rbcL + ITS2*. The evolutionary history was inferred using the Neighbor-Joining method. The optimal tree with the sum of branch length = 0.02267872 is shown. The percentage of replicate trees in which the associated taxa clustered together in the bootstrap test (500 replicates) are shown next to the branches. The tree is drawn to scale, with branch lengths in the same units as those of the evolutionary distances used to infer the phylogenetic tree. The evolutionary distances were computed using the Maximum Composite Likelihood method and are in the units of the number of base substitutions per site. The analysis involved 41 nucleotide sequences. Codon positions included were 1st+2nd+3rd+Noncoding. All positions containing gaps and missing data were eliminated. There were a total of 1435 positions in the final dataset. Evolutionary analyses were conducted in MEGA5.
(PDF)

S21 Fig Phylogenetic tree *Panax ginseng matK*. The evolutionary history was inferred using the Neighbor-Joining method. The optimal tree with the sum of branch length = 0.24952737 is shown. The percentage of replicate trees in which the associated taxa clustered together in the bootstrap test (500 replicates) are shown next to the branches. The tree is drawn to scale, with branch lengths in the same units as those of the evolutionary distances used to infer the phylogenetic tree. The evolutionary distances were computed using the Maximum Composite Likelihood method and are in the units of the number of base substitutions per site. The analysis involved 180 nucleotide sequences. Codon positions included were 1st+2nd+3rd+Noncoding. All positions containing gaps and missing data were eliminated. There were a total of 490 positions in the final dataset. Evolutionary analyses were conducted in MEGA5.
(PDF)

S22 Fig Phylogenetic tree *Panax ginseng rbcL*. The evolutionary history was inferred using the Neighbor-Joining method. The optimal tree with the sum of branch length = 0.10182289 is shown. The percentage of replicate trees in which the associated taxa clustered together in the bootstrap test (500 replicates) are shown next to the branches. The tree is drawn to scale, with branch lengths in the same units as those of the evolutionary distances used to infer the phylogenetic tree. The evolutionary distances were computed using the Maximum Composite Likelihood method and are in the units of the number of base substitutions per site. The analysis involved 72 nucleotide sequences. Codon positions included were 1st+2nd+3rd+Noncoding. All positions containing gaps and missing data were eliminated. There were a total of 360 positions in the final dataset. Evolutionary analyses were conducted in MEGA5.
(PDF)

S23 Fig Phylogenetic tree *Panax ginseng ITS2*. The evolutionary history was inferred using the Neighbor-Joining method. The optimal tree with the sum of branch length = 1,22004815 is shown. The percentage of replicate trees in which the associated taxa clustered together in the bootstrap test (500 replicates) are shown next to the branches. The tree is drawn to scale, with branch lengths in the same units as those of the evolutionary distances used to infer the phylogenetic tree. The evolutionary distances were computed using the Maximum Composite Likelihood method and are in the units of the number of base substitutions per site. The analysis involved 234 nucleotide sequences. Codon positions included were 1st+2nd+3rd+Noncoding. All positions containing gaps and missing data were eliminated. There were a total of 78 positions in the final dataset. Evolutionary analyses were conducted in MEGA5.
(PDF)

S24 Fig Phylogenetic tree *Panax ginseng matK + rbcL*. The evolutionary history was inferred using the Neighbor-Joining method. The optimal tree with the sum of branch length = 0.27861795 is shown. The percentage of replicate trees in which the associated taxa clustered together in the bootstrap test (500 replicates) are shown next to the branches. The tree is drawn to scale, with branch lengths in the same units as those of the evolutionary distances used to infer the phylogenetic tree. The evolutionary distances were computed using the Maximum Composite Likelihood method and are in the units of the number of base substitutions per site. The analysis involved 72 nucleotide sequences. Codon positions included were 1st+2nd+3rd+Noncoding. All positions containing gaps and missing data were eliminated. There were a total of 850 positions in the final dataset. Evolutionary analyses were conducted in MEGA5.
(PDF)

S25 Fig Phylogenetic tree *Panax ginseng matK + rbcL + ITS2*. The evolutionary history was inferred using the Neighbor-Joining method. The optimal tree with the sum of branch length = 0.29889235 is shown. The percentage of replicate trees in which the associated taxa clustered together in the bootstrap test (500 replicates) are shown next to the branches. The tree is drawn to scale, with branch lengths in the same units as those of the evolutionary distances used to infer the phylogenetic tree. The evolutionary distances were computed using the Maximum Composite Likelihood method and are in the units of the number of base substitutions per site. The analysis involved 65 nucleotide sequences. Codon positions included were 1st+2nd+3rd+Noncoding. All positions containing gaps and missing data were eliminated. There were a total of 935 positions in the final dataset. Evolutionary analyses were conducted in MEGA5.
(PDF)

S26 Fig Phylogenetic tree *Passiflora incarnata matK*. The evolutionary history was inferred using the Neighbor-Joining method. The optimal tree with the sum of branch length = 0.83753081 is shown. The percentage of replicate trees in which the associated taxa clustered together in the bootstrap test (500 replicates) are shown next to the branches. The tree is drawn to scale, with branch lengths in the same units as those of the evolutionary distances used to infer the phylogenetic tree. The evolutionary distances were computed using the Kimura 2-parameter method and are in the units of the number of base substitutions per site. The analysis involved 191 nucleotide sequences. Codon positions included were 1st+2nd+3rd+Noncoding. All positions containing gaps and missing data were eliminated. There were a total of 530 positions in the final dataset. Evolutionary analyses were conducted in MEGA5.
(PDF)

S27 Fig Phylogenetic tree *Passiflora incarnata rbcL*. The evolutionary history was inferred using the Neighbor-Joining method. The optimal tree with the sum of branch length = 0.64321259 is shown. The percentage of replicate trees in which the associated taxa clustered together in the bootstrap test (500 replicates) are shown next to the branches. The tree is drawn to scale, with branch lengths in the same units as those of the evolutionary distances used to infer the phylogenetic tree. The evolutionary distances were computed using the Kimura 2-parameter method and are in the units of the number of base substitutions per site. The analysis involved 234 nucleotide sequences. Codon positions included were 1st+2nd+3rd+Noncoding. All positions containing gaps and missing data were eliminated. There were a total of 512 positions in the final dataset. Evolutionary analyses were conducted in MEGA5.
(PDF)

S28 Fig Phylogenetic tree *Passiflora incarnata ITS2*. The evolutionary history was inferred using the Neighbor-Joining method. The optimal tree with the sum of branch length = 2.20614488 is shown. The percentage of replicate trees in which the associated taxa clustered together in the bootstrap test (500 replicates) are shown next to the branches. The tree is drawn to scale, with branch lengths in the same units as those of the evolutionary distances used to infer the phylogenetic tree. The evolutionary distances were computed using the Kimura 2-parameter method and are in the units of the number of base substitutions per site. The analysis involved 259 nucleotide sequences. Codon positions included were 1st+2nd+3rd+Noncoding. All positions containing gaps and missing data were eliminated. There were a total of 77 positions in the final dataset. Evolutionary analyses were conducted in MEGA5.
(PDF)

S29 Fig Phylogenetic tree *Passiflora incarnata matK + rbcL*. The evolutionary history was inferred using the Neighbor-Joining method. The optimal tree with the sum of branch length = 0.46000144 is shown. The percentage of replicate trees in which the associated taxa clustered together in the bootstrap test (500 replicates) are shown next to the branches. The tree is drawn to scale, with branch lengths in the same units as those of the evolutionary distances used to infer the phylogenetic tree. The evolutionary distances were computed using the Kimura 2-parameter method and are in the units of the number of base substitutions per site. The analysis involved 64 nucleotide sequences. Codon positions included were 1st+2nd+3rd+Noncoding. All positions containing gaps and missing data were eliminated. There were a total of 1045 positions in the final dataset. Evolutionary analyses were conducted in MEGA5.
(PDF)

S30 Fig Phylogenetic tree *Passiflora incarnata matK + rbcL + ITS2*. The evolutionary history was inferred using the Neighbor-Joining method. The optimal tree with the sum of branch length = 0.49341320 is shown. The percentage of replicate trees in which the associated taxa clustered together in the bootstrap test (500 replicates) are shown next to the branches. The tree is drawn to scale, with branch lengths in the same units as those of the evolutionary distances used to infer the phylogenetic tree. The evolutionary distances were computed using the Kimura 2-parameter method and are in the units of the number of base substitutions per site. The analysis involved 46 nucleotide sequences. Codon positions included were 1st+2nd+3rd+Noncoding. All positions containing gaps and missing data were eliminated. There were a total of 1191 positions in the final dataset. Evolutionary analyses were conducted in MEGA5.
(PDF)

S31 Fig Phylogenetic tree *Peumus boldus matK*. The evolutionary history was inferred using the Neighbor-Joining method. The optimal tree with the sum of branch length = 0.46390557 is shown. The percentage of replicate trees in which the associated taxa clustered together in the bootstrap test (500 replicates) are shown next to the branches. The tree is drawn to scale, with branch lengths in the same units as those of the evolutionary distances used to infer the phylogenetic tree. The evolutionary distances were computed using the Kimura 2-parameter method and are in the units of the number of base substitutions per site. The analysis involved 53 nucleotide sequences. Codon positions included were 1st+2nd+3rd+Noncoding. All positions containing gaps and missing data were eliminated. There were a total of 421 positions in the final dataset. Evolutionary analyses were conducted in MEGA5.
(PDF)

S32 Fig Phylogenetic tree *Peumus boldus rbcL*. The evolutionary history was inferred using the Neighbor-Joining method. The optimal tree with the sum of branch length = 0.13298911 is shown. The percentage of replicate trees in which the associated taxa clustered together in the bootstrap test (500 replicates) are shown next to the branches. The tree is drawn to scale, with branch lengths in the same units as those of the evolutionary distances used to infer the phylogenetic tree. The evolutionary distances were computed using the Kimura 2-parameter method and are in the units of the number of base substitutions per site. The analysis involved 53 nucleotide sequences. Codon positions included were 1st+2nd+3rd+Noncoding. All positions containing gaps and missing data were eliminated. There were a total of 502 positions in the final dataset. Evolutionary analyses were conducted in MEGA5.
(PDF)

S33 Fig Phylogenetic tree *Peumus boldus ITS2*. The evolutionary history was inferred using the Neighbor-Joining method. The optimal tree with the sum of branch length = 0.40028624 is shown. The percentage of replicate trees in which the associated taxa clustered together in the bootstrap test (500 replicates) are shown next to the branches. The tree is drawn to scale, with branch lengths in the same units as those of the evolutionary distances used to infer the phylogenetic tree. The evolutionary distances were computed using the Kimura 2-parameter method and are in the units of the number of base substitutions per site. The analysis involved 92 nucleotide sequences. Codon positions included were 1st+2nd+3rd+Noncoding. All positions containing gaps and missing data were eliminated. There were a total of 65 positions in the final dataset. Evolutionary analyses were conducted in MEGA5.
(PDF)

S34 Fig Phylogenetic tree *Peumus boldus matK + rbcL*. The evolutionary history was inferred using the Neighbor-Joining method. The optimal tree with the sum of branch length = 0.22642831 is shown. The percentage of replicate trees in which the associated taxa clustered together in the bootstrap test (500 replicates) are shown next to the branches. The tree is drawn to scale, with branch lengths in the same units as those of the evolutionary distances used to infer the phylogenetic tree. The evolutionary distances were computed using the Kimura 2-parameter method and are in the units of the number of base substitutions per site. The analysis involved 48 nucleotide sequences. Codon positions included were 1st+2nd+3rd+Noncoding. All positions containing gaps and missing data were eliminated. There were a total of 928 positions in the final dataset. Evolutionary analyses were conducted in MEGA5.
(PDF)

S35 Fig Phylogenetic tree *Peumus boldus matK + rbcL + ITS2*. The evolutionary history was inferred using the Neighbor-Joining method. The optimal tree with the sum of branch length = 0.17636441 is shown. The percentage of replicate trees in which the associated taxa clustered together in the bootstrap test (500 replicates) are shown next to the branches. The tree is drawn to scale, with branch lengths in the same units as those of the evolutionary distances used to infer the phylogenetic tree. The evolutionary distances were computed using the Kimura 2-parameter method and are in the units of the number of base substitutions per site. The analysis involved 31 nucleotide sequences. Codon positions included were 1st+2nd+3rd+Noncoding. All positions containing gaps and missing data were eliminated. There were a total of 998 positions in the final dataset. Evolutionary analyses were conducted in MEGA5.
(PDF)

S36 Fig Phylogenetic tree *Valeriana officinalis matK*. The evolutionary history was inferred using the Neighbor-Joining method. The optimal tree with the sum of branch length = 1.06223373 is shown. The percentage of replicate trees in which the associated taxa clustered together in the bootstrap test (500 replicates) are shown next to the branches. The tree is drawn to scale, with branch lengths in the same units as those of the evolutionary distances used to infer the phylogenetic tree. The evolutionary distances were computed using the Kimura 2-parameter method and are in the units of the number of base substitutions per site. The analysis involved 125 nucleotide sequences. Codon positions included were 1st+2nd+3rd+Noncoding. All positions containing gaps and missing data were eliminated. There were a total of 525 positions in the final dataset. Evolutionary analyses were conducted in MEGA5.
(PDF)

S37 Fig Phylogenetic tree *Valeriana officinalis rbcL*. The evolutionary history was inferred using the Neighbor-Joining method. The optimal tree with the sum of branch length = 0.45263449 is shown. The percentage of replicate trees in which the associated taxa clustered together in the bootstrap test (500 replicates) are shown next to the branches. The tree is drawn to scale, with branch lengths in the same units as those of the evolutionary distances used to infer the phylogenetic tree. The evolutionary distances were computed using the Kimura 2-parameter method and are in the units of the number of base substitutions per site. The analysis involved 155 nucleotide sequences. Codon positions included were 1st+2nd+3rd+Noncoding. All positions containing gaps and missing data were eliminated. There were a total of 474 positions in the final dataset. Evolutionary analyses were conducted in MEGA5.
(PDF)

S38 Fig Phylogenetic tree *Valeriana officinalis ITS2*. The evolutionary history was inferred using the Neighbor-Joining method. The optimal tree with the sum of branch length = 1.70998580 is shown. The percentage of replicate trees in which the associated taxa clustered together in the bootstrap test (500 replicates) are shown next to the branches. The tree is drawn to scale, with branch lengths in the same units as those of the evolutionary distances used to infer the phylogenetic tree. The evolutionary distances were computed using the Kimura 2-parameter method and are in the units of the number of base substitutions per site. The analysis involved 31 nucleotide sequences. Codon positions included were 1st+2nd+3rd+Noncoding. All positions containing gaps and missing data were eliminated. There were a total of 50 positions in the final dataset. Evolutionary analyses were conducted in MEGA5.
(PDF)

S39 Fig Phylogenetic tree *Valeriana officinalis matK + rbcL*. The evolutionary history was inferred using the Neighbor-Joining method. The optimal tree with the sum of branch length = 0.74255654 is shown. The percentage of replicate trees in which the associated taxa clustered together in the bootstrap test (500 replicates) are shown next to the branches. The tree is drawn to scale, with branch lengths in the same units as those of the evolutionary distances used to infer the phylogenetic tree. The evolutionary distances were computed using the Kimura 2-parameter method and are in the units of the number of base substitutions per site. The analysis involved 113 nucleotide sequences. Codon positions included were 1st+2nd+3rd+Noncoding. All positions containing gaps and missing data were eliminated. There were a total of 1001 positions in the final dataset. Evolutionary analyses were conducted in MEGA5.
(PDF)

S40 Fig Phylogenetic tree *Valeriana officinalis matK + rbcL + ITS2*. The evolutionary history was inferred using the Neighbor-Joining method. The optimal tree with the sum of branch length = 0.48881377 is shown. The percentage of replicate trees in which the associated taxa clustered together in the bootstrap test (500 replicates) are shown next to the branches. The tree is drawn to scale, with branch lengths in the same units as those of the evolutionary distances used to infer the phylogenetic tree. The evolutionary distances were computed using the Kimura 2-parameter method and are in the units of the number of base substitutions per site. The analysis involved 18 nucleotide sequences. Codon positions included were 1st+2nd+3rd+Noncoding. All positions containing gaps and missing data were eliminated. There were a total of 1121 positions in the final dataset. Evolutionary analyses were conducted in MEGA5.
(PDF)

S1 Table DNA Barcode identification, acession number and percentual of similiraty between the samples and the identified species on the Barcode of life Database or GenBank. (XLSX)

S1 File ITS2 sequences. The sequences present in this file had fewer than 200 base pairs and, therefore, could not be deposited in GenBank.
(TXT)
