# Supplementary figures and images for: Medicinal Plants Recommended by the World Health Organization: DNA Barcode Identification Associated with Chemical Analyses Guarantees Their Quality
Source: PLoS One. 2015 May 15;10(5):e0127866. doi: 10.1371/journal.pone.0127866 (PMC4433216; doi:10.1371/journal.pone.0127866)

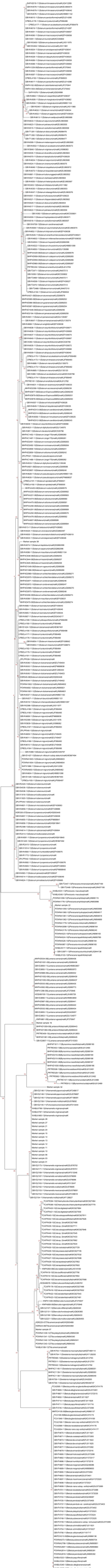

Supplement: S1 Fig — The evolutionary history was inferred using the Neighbor-Joining method. The optimal tree with the sum of branch length = 1.47656508 is shown. The percentage of replicate trees in which the associated taxa clustered together in the bootstrap test (500 replicates) are shown next to the branches. The tree is drawn to scale, with branch lengths in the same units as those of the evolutionary distances used to infer the phylogenetic tree. The evolutionary distances were computed using the Maximum Composite Likelihood method and are in the units of the number of base substitutions per site. The analysis involved 442 nucleotide sequences. Codon positions included were 1st+2nd+3rd+Noncoding. All positions containing gaps and missing data were eliminated. There were a total of 205 positions in the final dataset. Evolutionary analyses were conducted in MEGA5. (PDF) [file pone.0127866.s001.pdf]

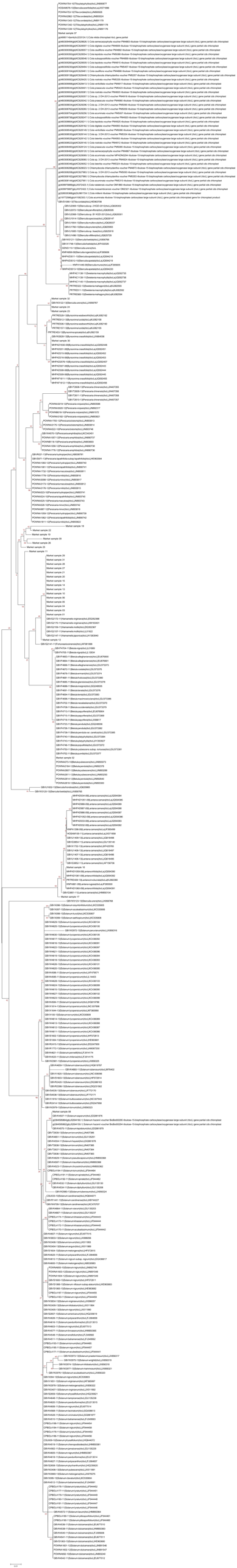

Supplement: S2 Fig — The evolutionary history was inferred using the Neighbor-Joining method. The optimal tree with the sum of branch length = 0.67568534 is shown. The percentage of replicate trees in which the associated taxa clustered together in the bootstrap test (500 replicates) are shown next to the branches. The tree is drawn to scale, with branch lengths in the same units as those of the evolutionary distances used to infer the phylogenetic tree. The evolutionary distances were computed using the Maximum Composite Likelihood method and are in the units of the number of base substitutions per site. The analysis involved 370 nucleotide sequences. Codon positions included were 1st+2nd+3rd+Noncoding. All positions containing gaps and missing data were eliminated. There were a total of 215 positions in the final dataset. Evolutionary analyses were conducted in MEGA5. (PDF) [file pone.0127866.s002.pdf]

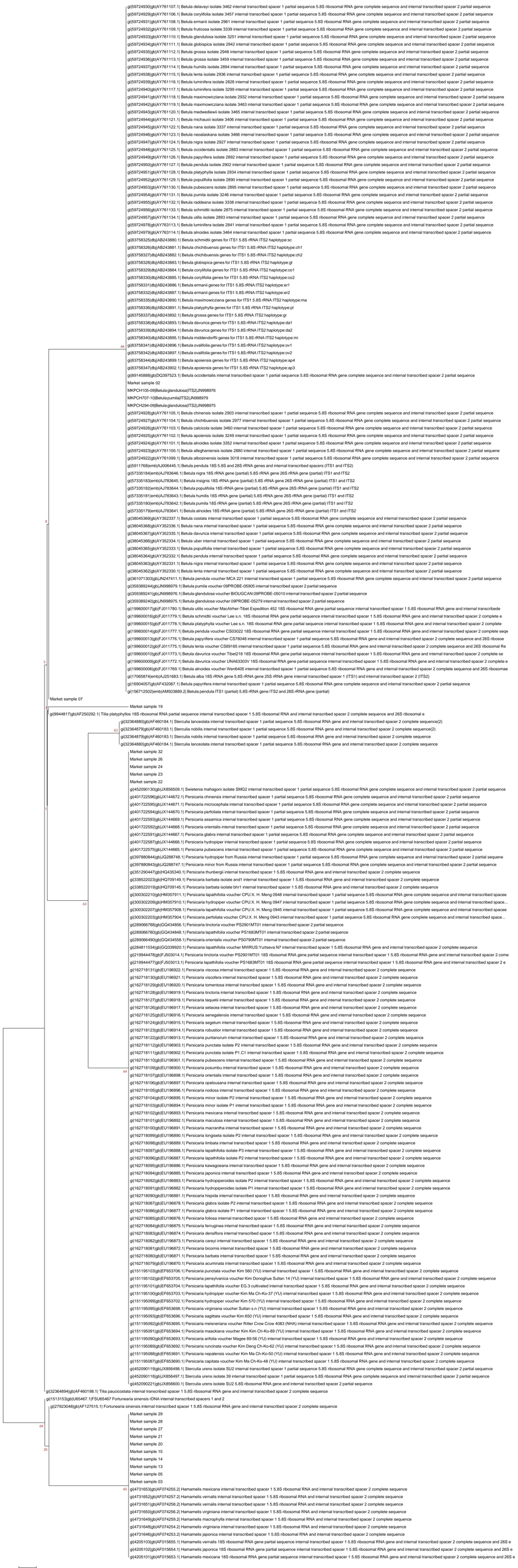

Supplement: S3 Fig — The evolutionary history was inferred using the Neighbor-Joining method. The optimal tree with the sum of branch length = 0.71702123 is shown. The percentage of replicate trees in which the associated taxa clustered together in the bootstrap test (500 replicates) are shown next to the branches. The tree is drawn to scale, with branch lengths in the same units as those of the evolutionary distances used to infer the phylogenetic tree. The evolutionary distances were computed using the Maximum Composite Likelihood method and are in the units of the number of base substitutions per site. The analysis involved 207 nucleotide sequences. Codon positions included were 1st+2nd+3rd+Noncoding. All positions containing gaps and missing data were eliminated. There were a total of 13 positions in the final dataset. Evolutionary analyses were conducted in MEGA5. (PDF) [file pone.0127866.s003.pdf]

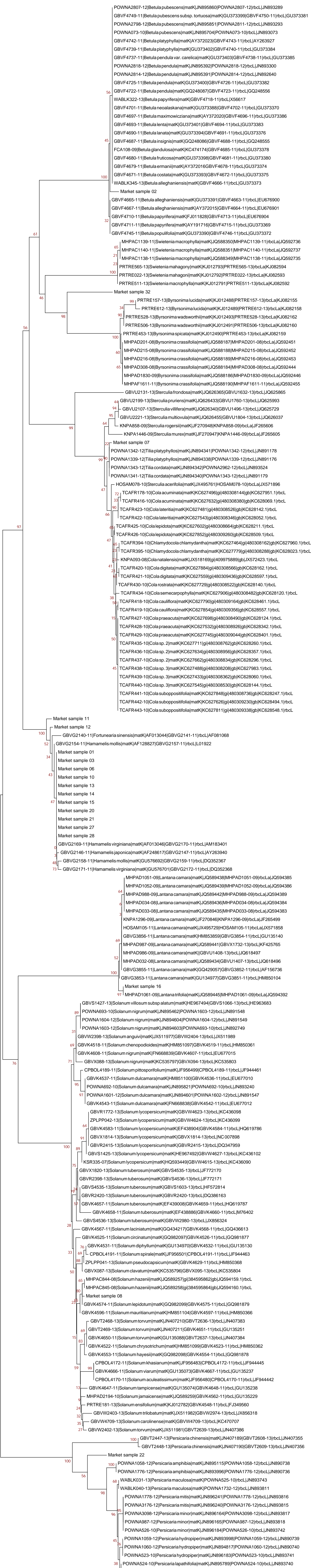

0.02

Supplement: S4 Fig — The evolutionary history was inferred using the Neighbor-Joining method. The optimal tree with the sum of branch length = 0.86650001 is shown. The percentage of replicate trees in which the associated taxa clustered together in the bootstrap test (500 replicates) are shown next to the branches. The tree is drawn to scale, with branch lengths in the same units as those of the evolutionary distances used to infer the phylogenetic tree. The evolutionary distances were computed using the Maximum Composite Likelihood method and are in the units of the number of base substitutions per site. The analysis involved 189 nucleotide sequences. Codon positions included were 1st+2nd+3rd+Noncoding. All positions containing gaps and missing data were eliminated. There were a total of 620 positions in the final dataset. Evolutionary analyses were conducted in MEGA5. (PDF) [file pone.0127866.s004.pdf]

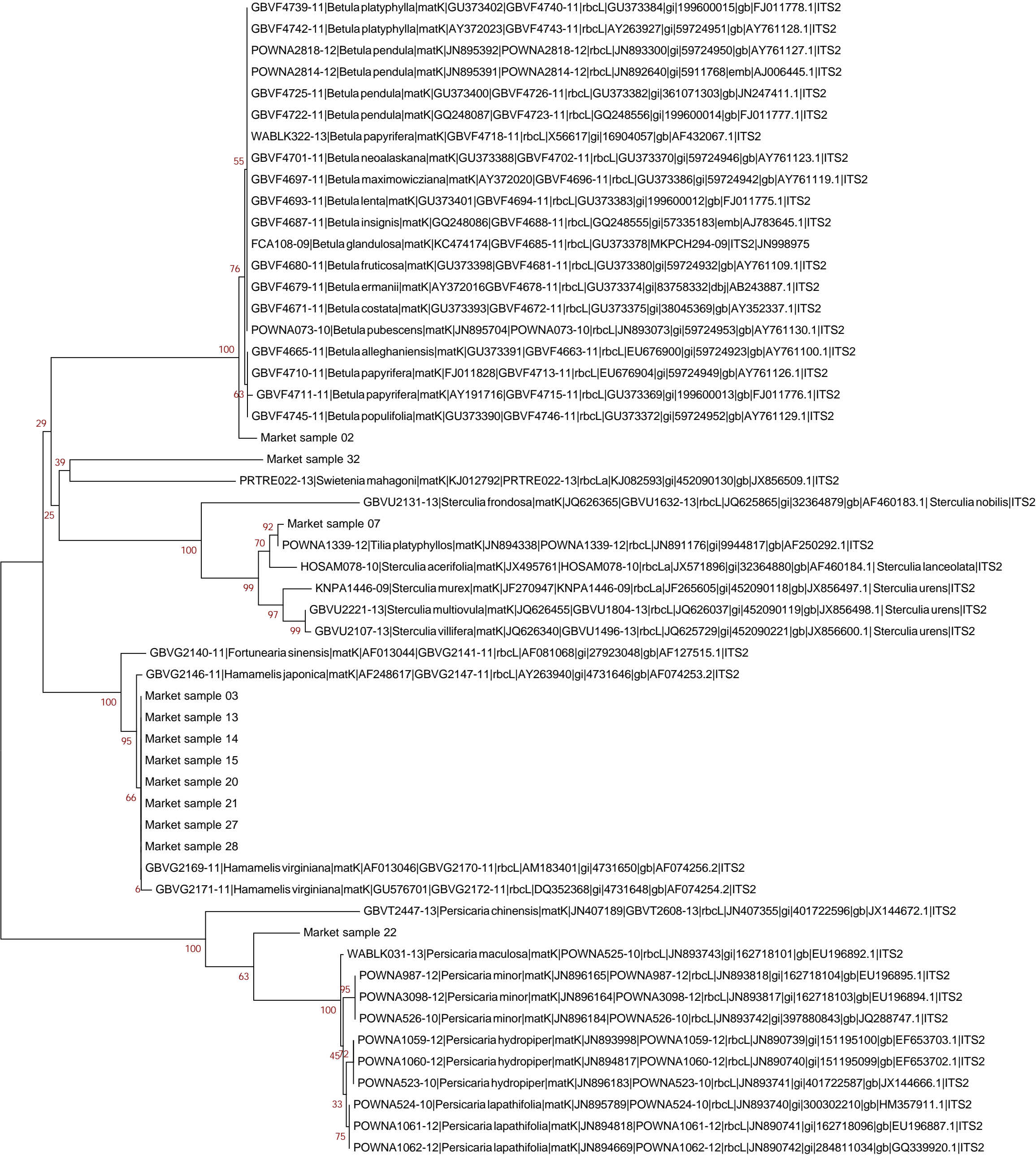

0.02

Supplement: S5 Fig — The evolutionary history was inferred using the Neighbor-Joining method. The optimal tree with the sum of branch length = 0.51134035 is shown. The percentage of replicate trees in which the associated taxa clustered together in the bootstrap test (500 replicates) are shown next to the branches. The tree is drawn to scale, with branch lengths in the same units as those of the evolutionary distances used to infer the phylogenetic tree. The evolutionary distances were computed using the Maximum Composite Likelihood method and are in the units of the number of base substitutions per site. The analysis involved 54 nucleotide sequences. Codon positions included were 1st+2nd+3rd+Noncoding. All positions containing gaps and missing data were eliminated. There were a total of 726 positions in the final dataset. Evolutionary analyses were conducted in MEGA5. (PDF) [file pone.0127866.s005.pdf]

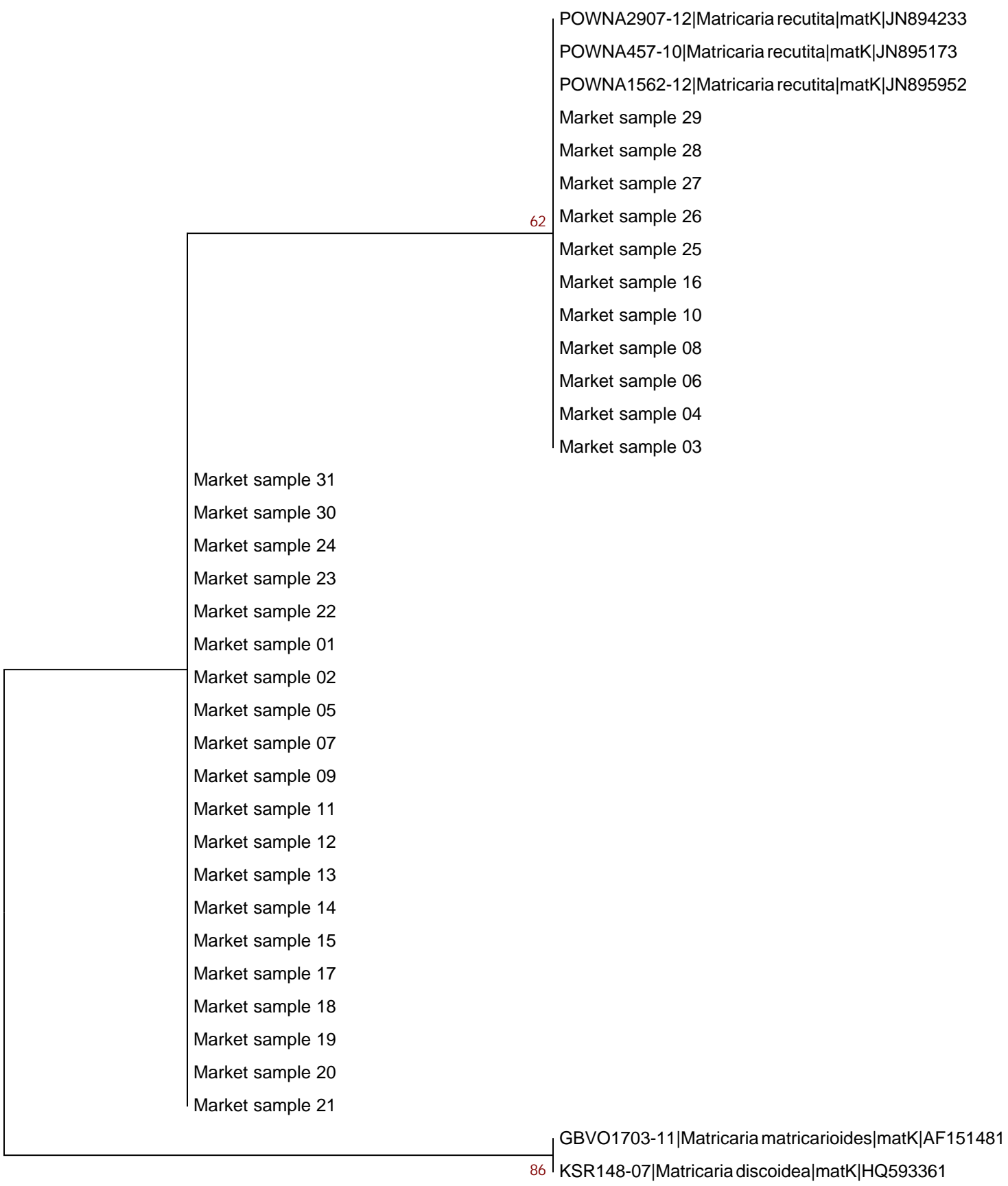

0.0005

Supplement: S6 Fig — The evolutionary history was inferred using the Neighbor-Joining method. The optimal tree with the sum of branch length = 0.00478244 is shown. The percentage of replicate trees in which the associated taxa clustered together in the bootstrap test (500 replicates) are shown next to the branches. The tree is drawn to scale, with branch lengths in the same units as those of the evolutionary distances used to infer the phylogenetic tree. The evolutionary distances were computed using the Maximum Composite Likelihood method and are in the units of the number of base substitutions per site. The analysis involved 36 nucleotide sequences. Codon positions included were 1st+2nd+3rd+Noncoding. All positions containing gaps and missing data were eliminated. There were a total of 631 positions in the final dataset. Evolutionary analyses were conducted in MEGA5. (PDF) [file pone.0127866.s006.pdf]

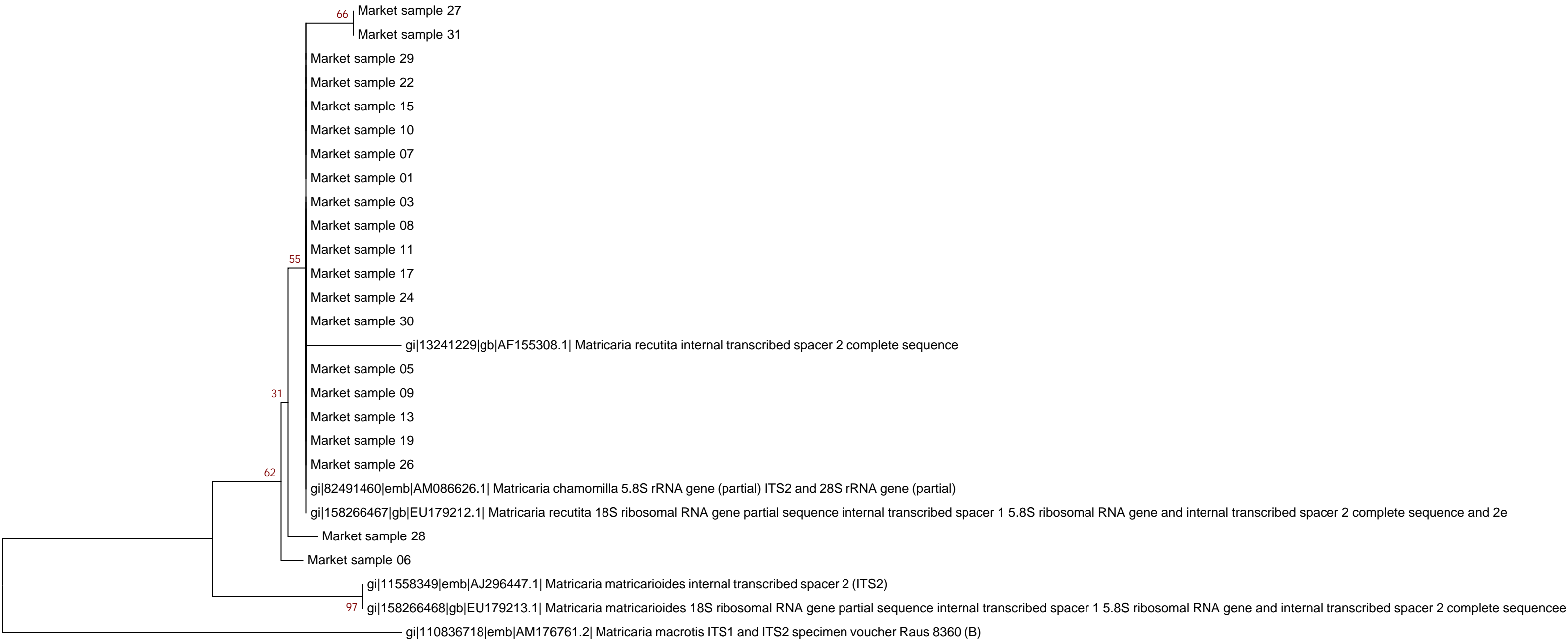

0.01

Supplement: S8 Fig — The evolutionary history was inferred using the Neighbor-Joining method. The optimal tree with the sum of branch length = 0.12287265 is shown. The percentage of replicate trees in which the associated taxa clustered together in the bootstrap test (500 replicates) are shown next to the branches. The tree is drawn to scale, with branch lengths in the same units as those of the evolutionary distances used to infer the phylogenetic tree. The evolutionary distances were computed using the Maximum Composite Likelihood method and are in the units of the number of base substitutions per site. The analysis involved 27 nucleotide sequences. Codon positions included were 1st+2nd+3rd+Noncoding. All positions containing gaps and missing data were eliminated. There were a total of 191 positions in the final dataset. Evolutionary analyses were conducted in MEGA5. (PDF) [file pone.0127866.s008.pdf]

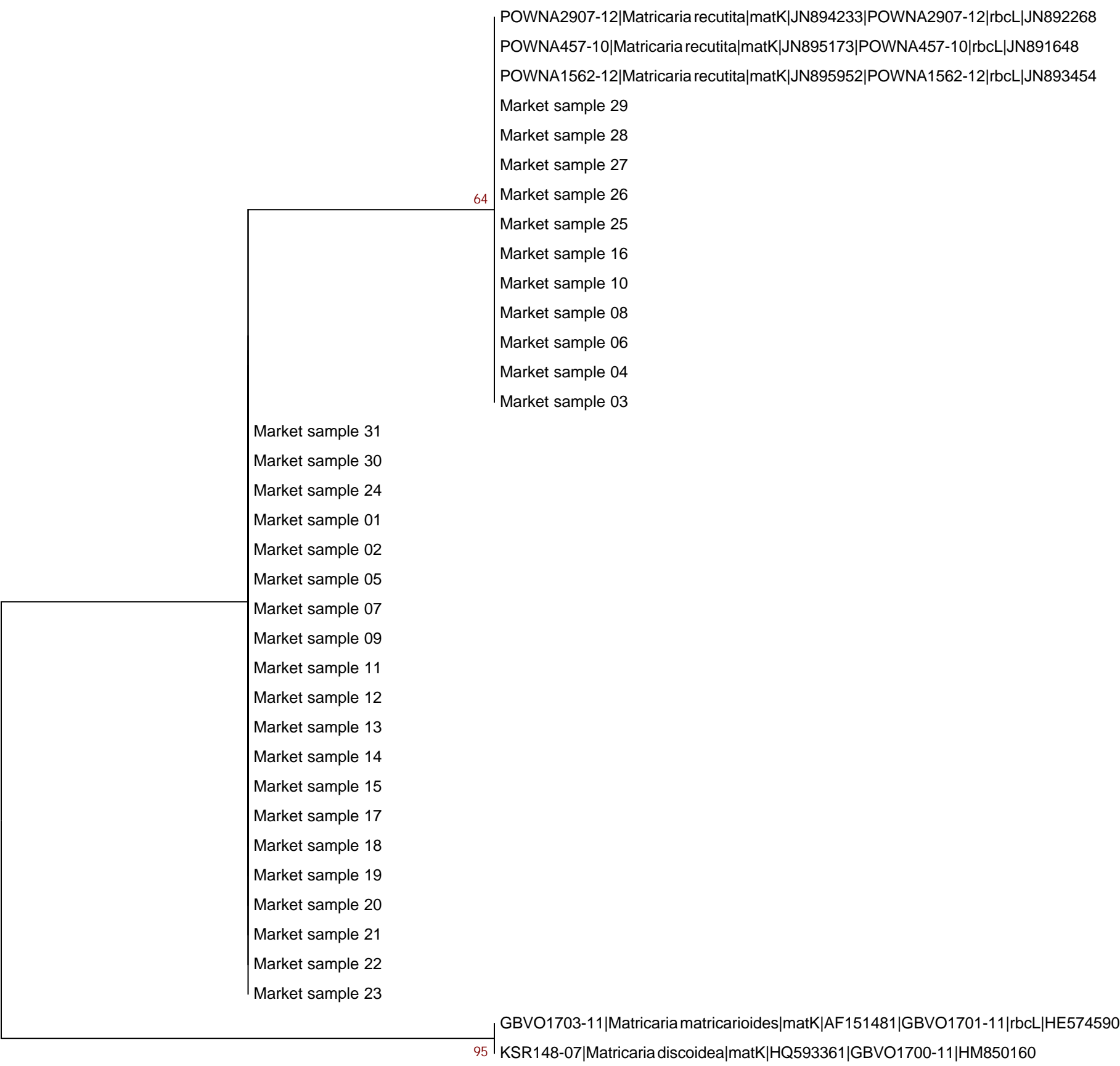

0.0005

Supplement: S9 Fig — The evolutionary history was inferred using the Neighbor-Joining method. The optimal tree with the sum of branch length = 0.00363776 is shown. The percentage of replicate trees in which the associated taxa clustered together in the bootstrap test (500 replicates) are shown next to the branches. The tree is drawn to scale, with branch lengths in the same units as those of the evolutionary distances used to infer the phylogenetic tree. The evolutionary distances were computed using the Maximum Composite Likelihood method and are in the units of the number of base substitutions per site. The analysis involved 36 nucleotide sequences. Codon positions included were 1st+2nd+3rd+Noncoding. All positions containing gaps and missing data were eliminated. There were a total of 1104 positions in the final dataset. Evolutionary analyses were conducted in MEGA5. (PDF) [file pone.0127866.s009.pdf]

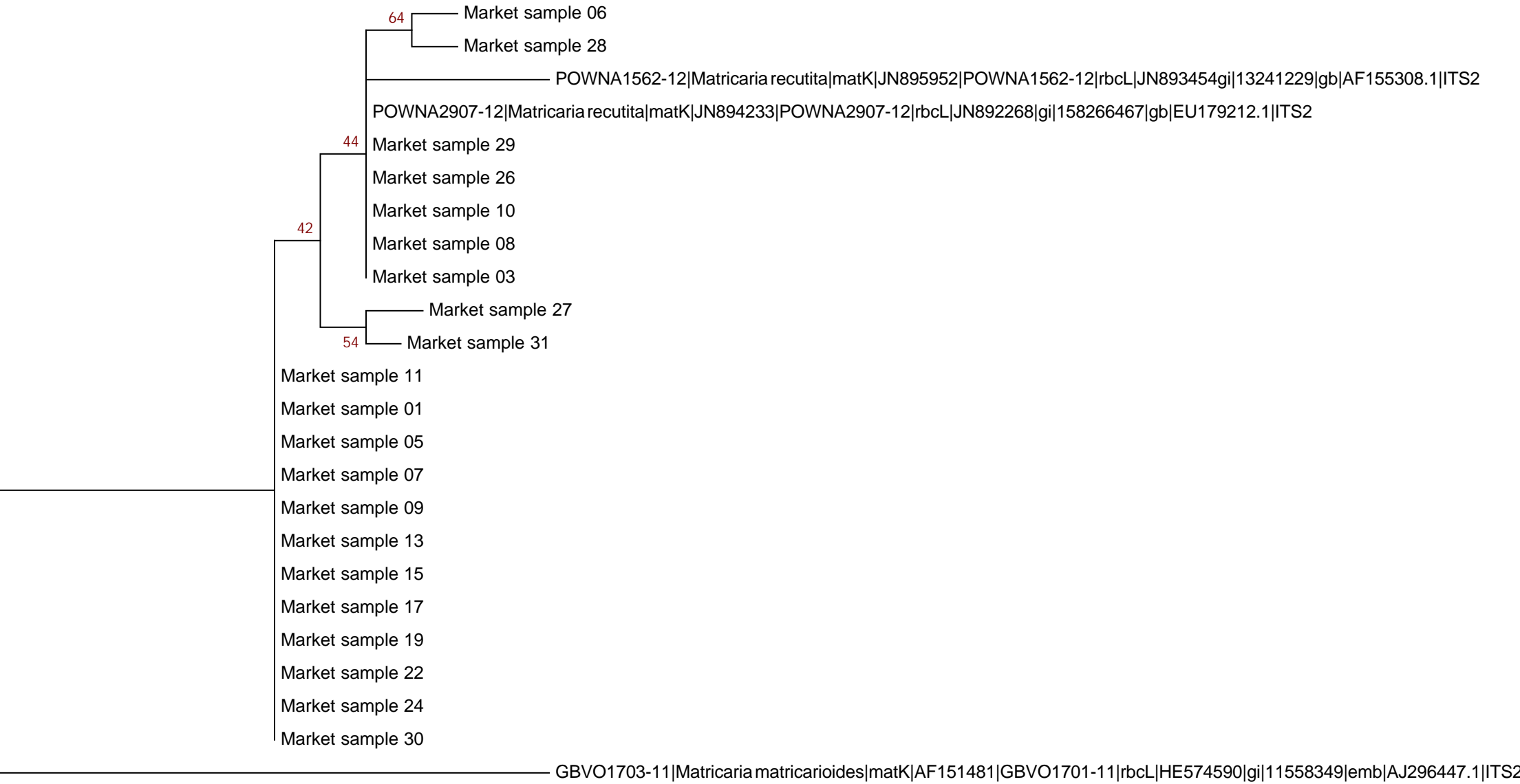

0.0005

Supplement: S10 Fig — The evolutionary history was inferred using the Neighbor-Joining method. The optimal tree with the sum of branch length = 0.00534820 is shown. The percentage of replicate trees in which the associated taxa clustered together in the bootstrap test (500 replicates) are shown next to the branches. The tree is drawn to scale, with branch lengths in the same units as those of the evolutionary distances used to infer the phylogenetic tree. The evolutionary distances were computed using the Maximum Composite Likelihood method and are in the units of the number of base substitutions per site. The analysis involved 24 nucleotide sequences. Codon positions included were 1st+2nd+3rd+Noncoding. All positions containing gaps and missing data were eliminated. There were a total of 1319 positions in the final dataset. Evolutionary analyses were conducted in MEGA5. (PDF) [file pone.0127866.s010.pdf]

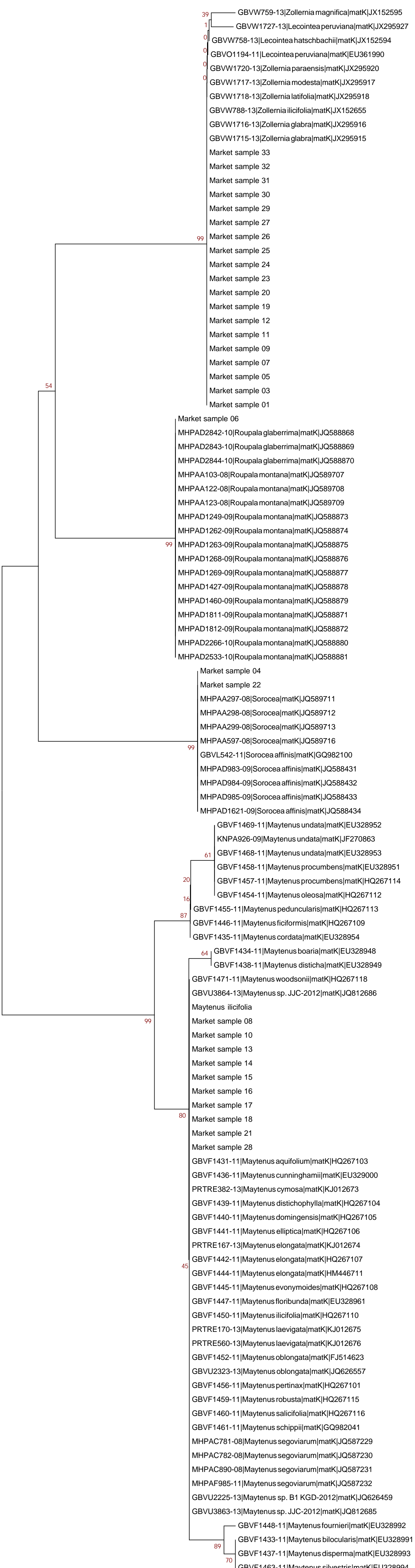

Supplement: S11 Fig — The evolutionary history was inferred using the Neighbor-Joining method. The optimal tree with the sum of branch length = 0.39435507 is shown. The percentage of replicate trees in which the associated taxa clustered together in the bootstrap test (500 replicates) are shown next to the branches. The tree is drawn to scale, with branch lengths in the same units as those of the evolutionary distances used to infer the phylogenetic tree. The evolutionary distances were computed using the Maximum Composite Likelihood method and are in the units of the number of base substitutions per site. The analysis involved 112 nucleotide sequences. Codon positions included were 1st+2nd+3rd+Noncoding. All positions containing gaps and missing data were eliminated. There were a total of 98 positions in the final dataset. Evolutionary analyses were conducted in MEGA5. (PDF) [file pone.0127866.s011.pdf]

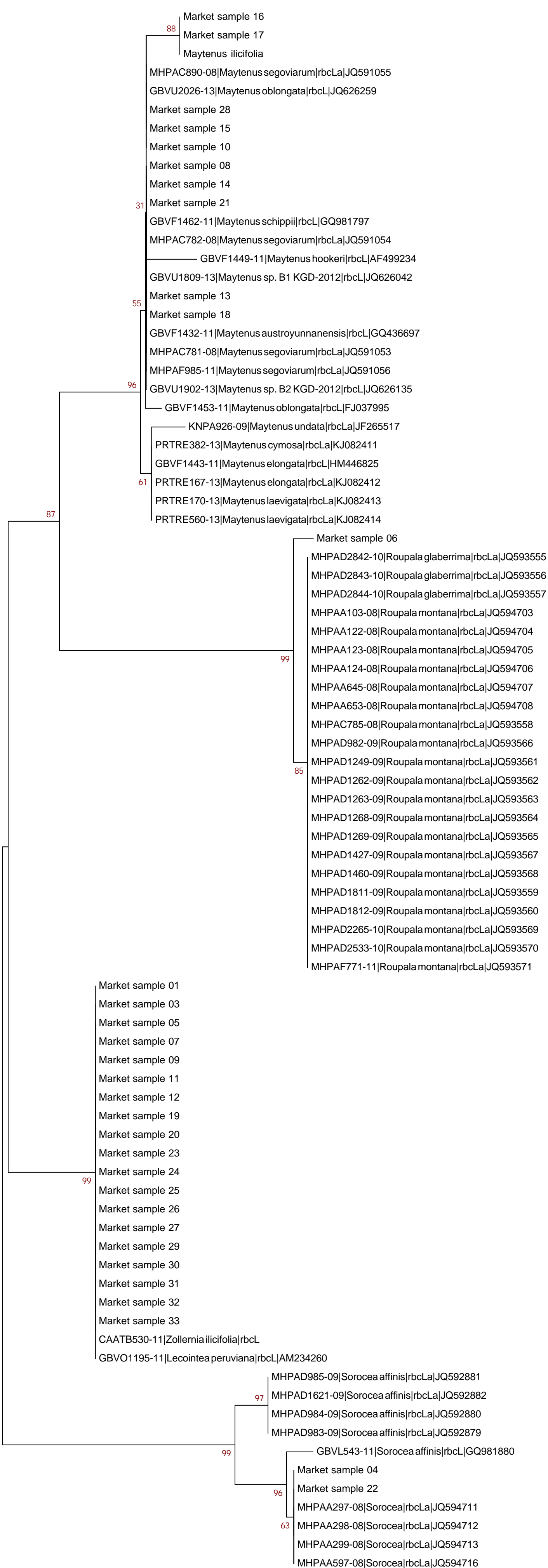

0.01

Supplement: S12 Fig — The evolutionary history was inferred using the Neighbor-Joining method. The optimal tree with the sum of branch length = 0.16105704 is shown. The percentage of replicate trees in which the associated taxa clustered together in the bootstrap test (500 replicates) are shown next to the branches. The tree is drawn to scale, with branch lengths in the same units as those of the evolutionary distances used to infer the phylogenetic tree. The evolutionary distances were computed using the Maximum Composite Likelihood method and are in the units of the number of base substitutions per site. The analysis involved 84 nucleotide sequences. Codon positions included were 1st+2nd+3rd+Noncoding. All positions containing gaps and missing data were eliminated. There were a total of 370 positions in the final dataset. Evolutionary analyses were conducted in MEGA5. (PDF) [file pone.0127866.s012.pdf]

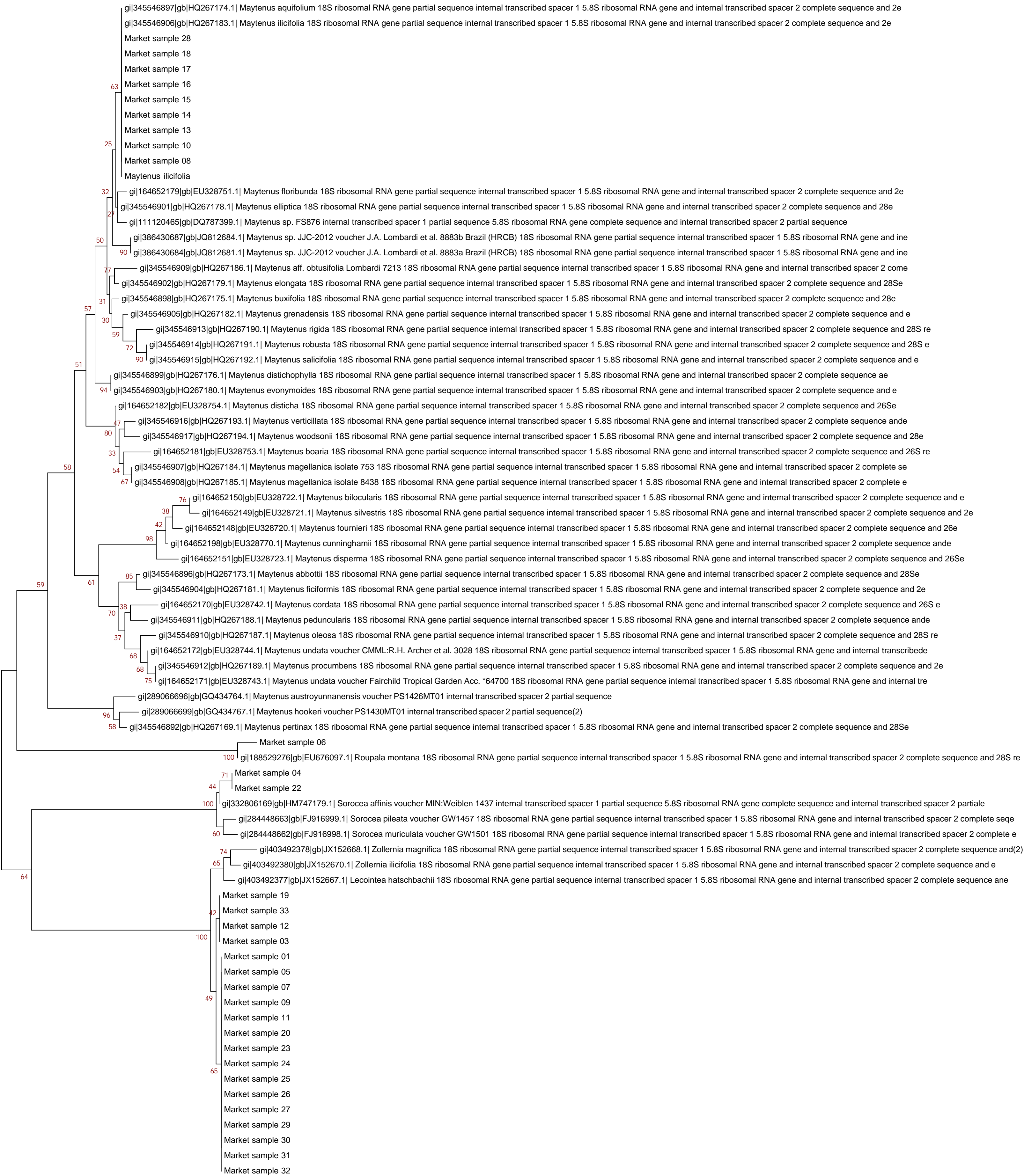

Supplement: S13 Fig — The evolutionary history was inferred using the Neighbor-Joining method. The optimal tree with the sum of branch length = 1.85107254 is shown. The percentage of replicate trees in which the associated taxa clustered together in the bootstrap test (500 replicates) are shown next to the branches. The tree is drawn to scale, with branch lengths in the same units as those of the evolutionary distances used to infer the phylogenetic tree. The evolutionary distances were computed using the Maximum Composite Likelihood method and are in the units of the number of base substitutions per site. The analysis involved 77 nucleotide sequences. Codon positions included were 1st+2nd+3rd+Noncoding. All positions containing gaps and missing data were eliminated. There were a total of 100 positions in the final dataset. Evolutionary analyses were conducted in MEGA5. (PDF) [file pone.0127866.s013.pdf]

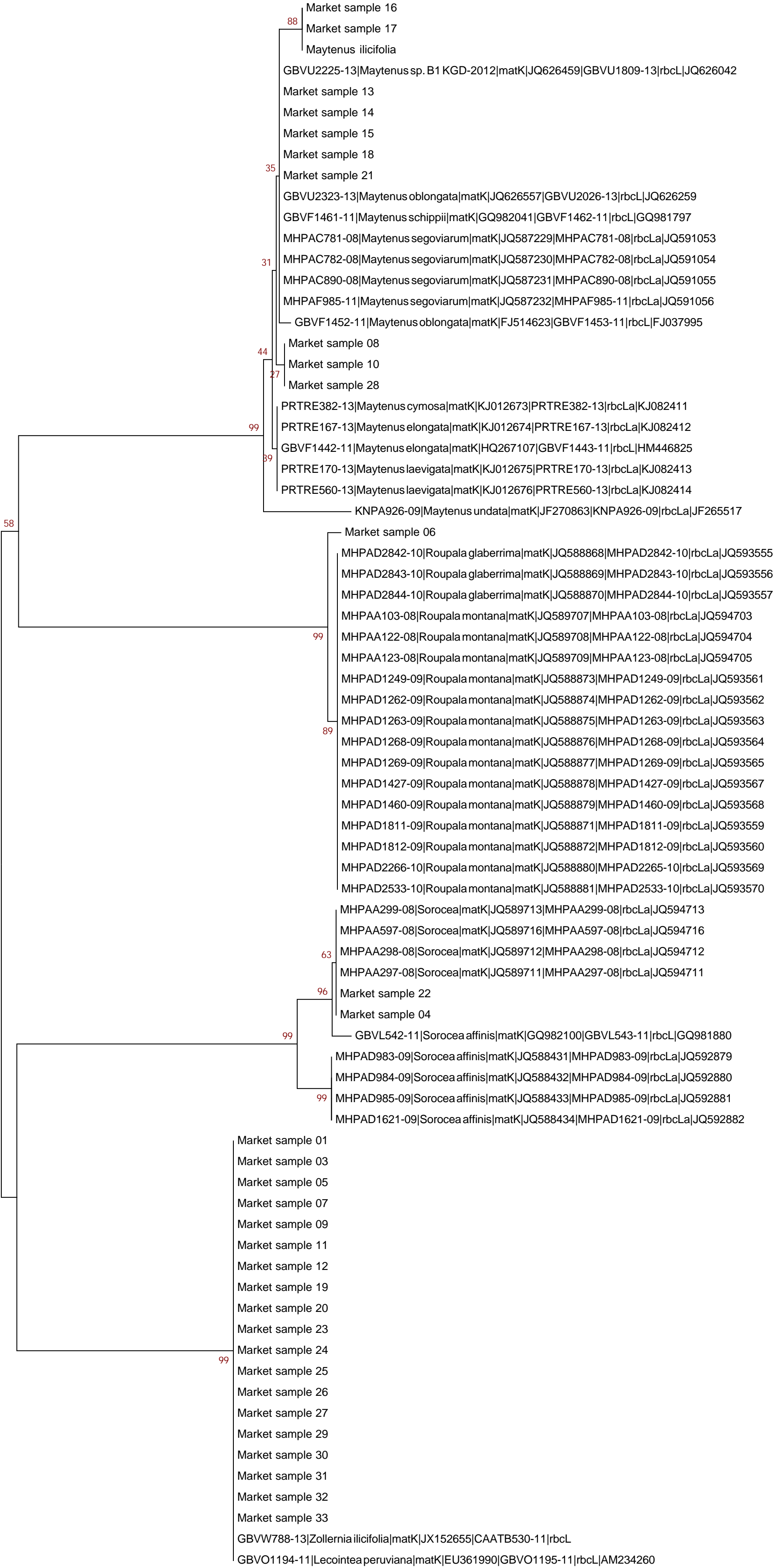

0.01

Supplement: S14 Fig — The evolutionary history was inferred using the Neighbor-Joining method. The optimal tree with the sum of branch length = 0.22908763 is shown. The percentage of replicate trees in which the associated taxa clustered together in the bootstrap test (500 replicates) are shown next to the branches. The tree is drawn to scale, with branch lengths in the same units as those of the evolutionary distances used to infer the phylogenetic tree. The evolutionary distances were computed using the Maximum Composite Likelihood method and are in the units of the number of base substitutions per site. The analysis involved 75 nucleotide sequences. Codon positions included were 1st+2nd+3rd+Noncoding. All positions containing gaps and missing data were eliminated. There were a total of 518 positions in the final dataset. Evolutionary analyses were conducted in MEGA5. (PDF) [file pone.0127866.s014.pdf]

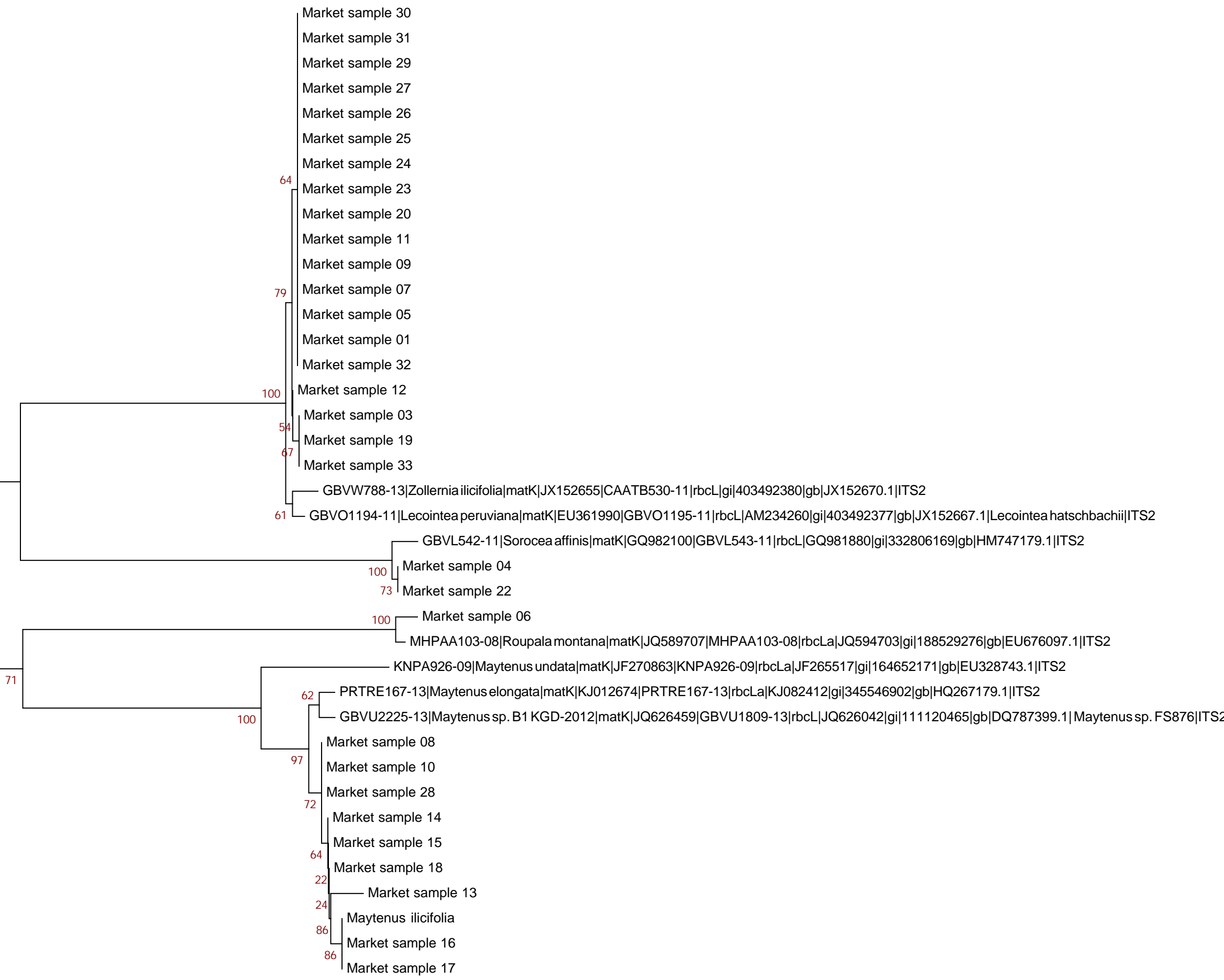

0.01

Supplement: S15 Fig — The evolutionary history was inferred using the Neighbor-Joining method. The optimal tree with the sum of branch length = 0.31908268 is shown. The percentage of replicate trees in which the associated taxa clustered together in the bootstrap test (500 replicates) are shown next to the branches. The tree is drawn to scale, with branch lengths in the same units as those of the evolutionary distances used to infer the phylogenetic tree. The evolutionary distances were computed using the Maximum Composite Likelihood method and are in the units of the number of base substitutions per site. The analysis involved 39 nucleotide sequences. Codon positions included were 1st+2nd+3rd+Noncoding. All positions containing gaps and missing data were eliminated. There were a total of 858 positions in the final dataset. Evolutionary analyses were conducted in MEGA5. (PDF) [file pone.0127866.s015.pdf]

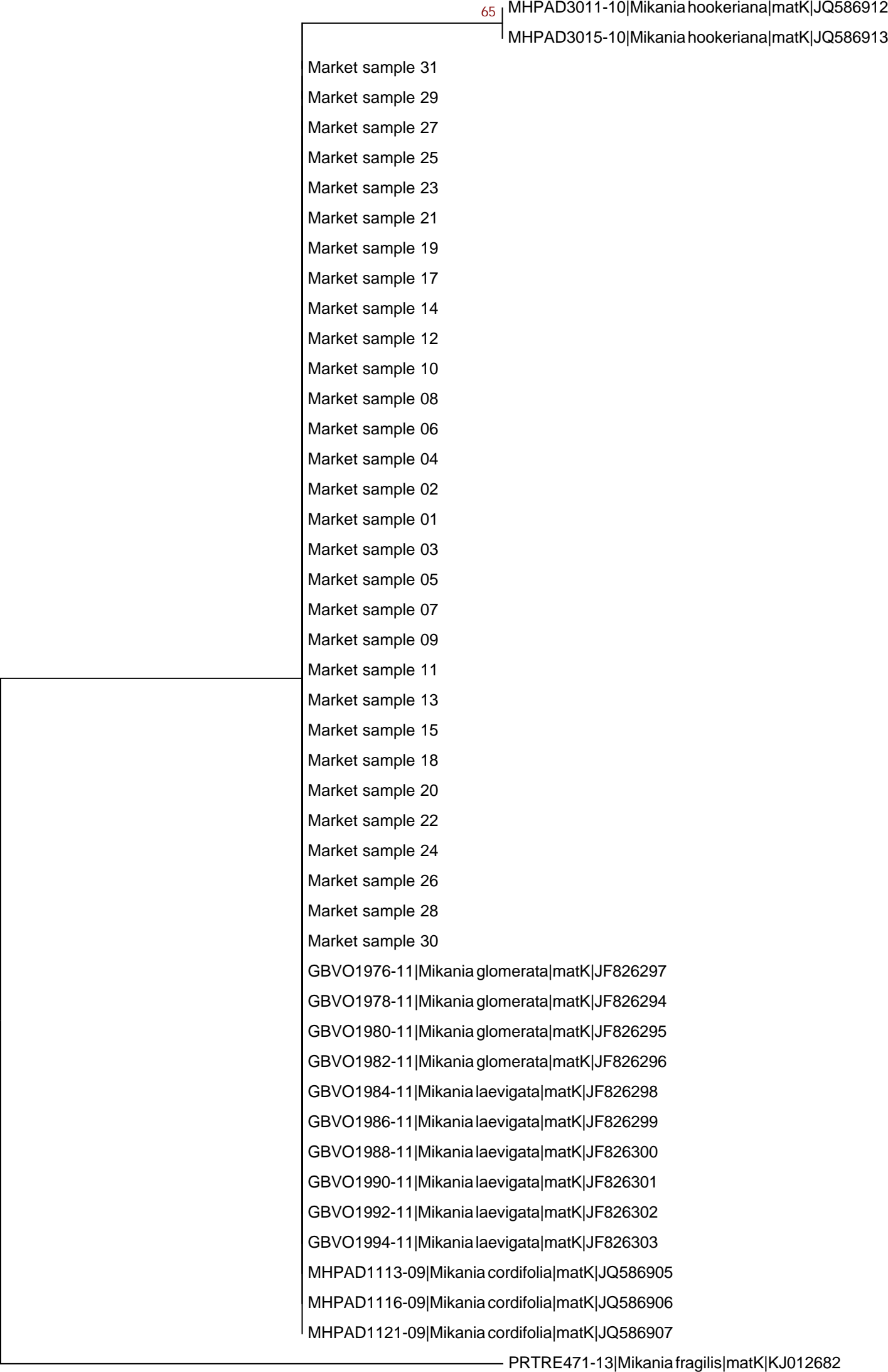

0.0005

Supplement: S16 Fig — The evolutionary history was inferred using the Neighbor-Joining method. The optimal tree with the sum of branch length = 0.00738890 is shown. The percentage of replicate trees in which the associated taxa clustered together in the bootstrap test (500 replicates) are shown next to the branches. The tree is drawn to scale, with branch lengths in the same units as those of the evolutionary distances used to infer the phylogenetic tree. The evolutionary distances were computed using the Maximum Composite Likelihood method and are in the units of the number of base substitutions per site. The analysis involved 46 nucleotide sequences. Codon positions included were 1st+2nd+3rd+Noncoding. All positions containing gaps and missing data were eliminated. There were a total of 679 positions in the final dataset. Evolutionary analyses were conducted in MEGA5. (PDF) [file pone.0127866.s016.pdf]

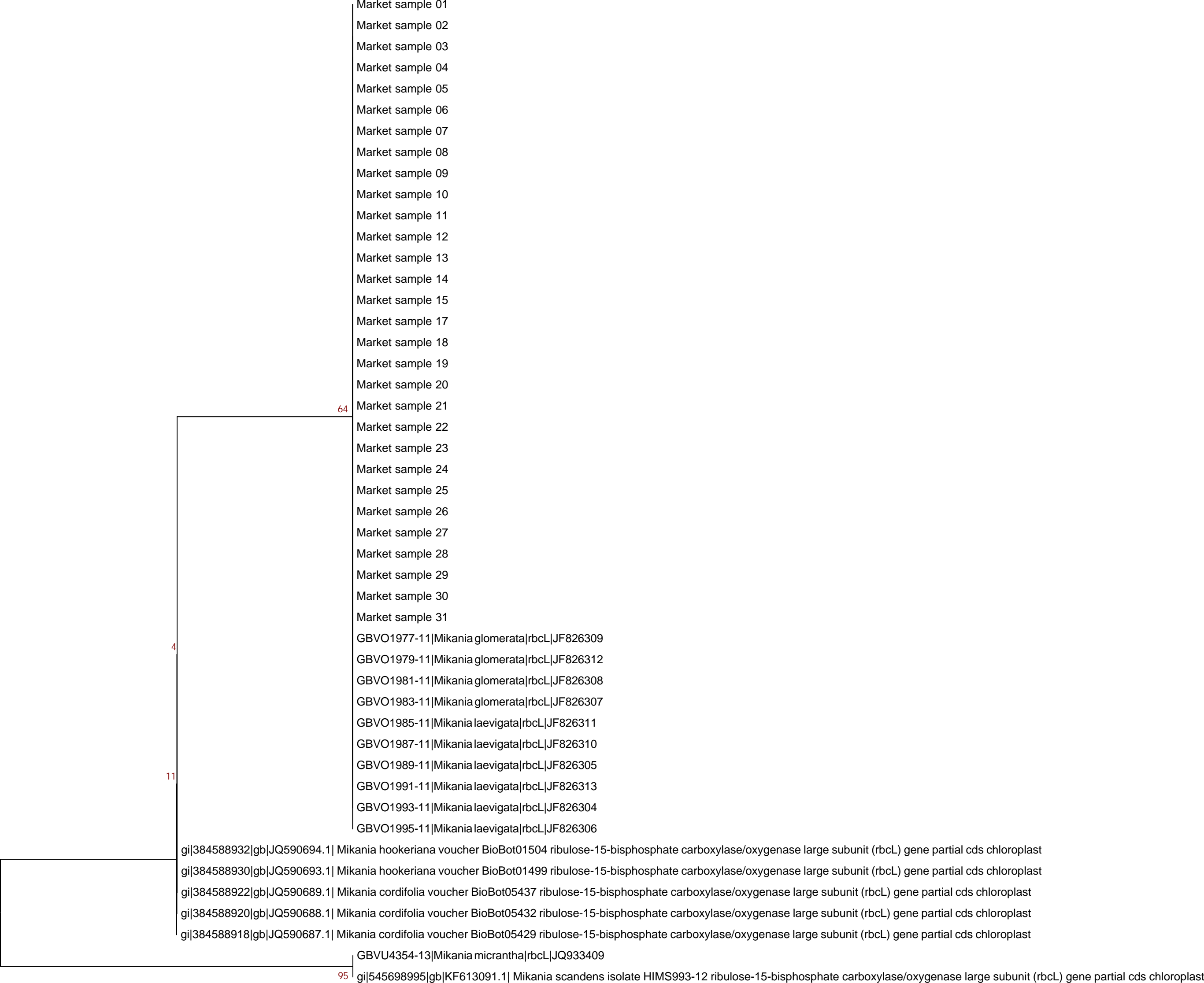

0.0005

Supplement: S17 Fig — The evolutionary history was inferred using the Neighbor-Joining method. The optimal tree with the sum of branch length = 0.00755703 is shown. The percentage of replicate trees in which the associated taxa clustered together in the bootstrap test (500 replicates) are shown next to the branches. The tree is drawn to scale, with branch lengths in the same units as those of the evolutionary distances used to infer the phylogenetic tree. The evolutionary distances were computed using the Maximum Composite Likelihood method and are in the units of the number of base substitutions per site. The analysis involved 47 nucleotide sequences. Codon positions included were 1st+2nd+3rd+Noncoding. All positions containing gaps and missing data were eliminated. There were a total of 531 positions in the final dataset. Evolutionary analyses were conducted in MEGA5. (PDF) [file pone.0127866.s017.pdf]

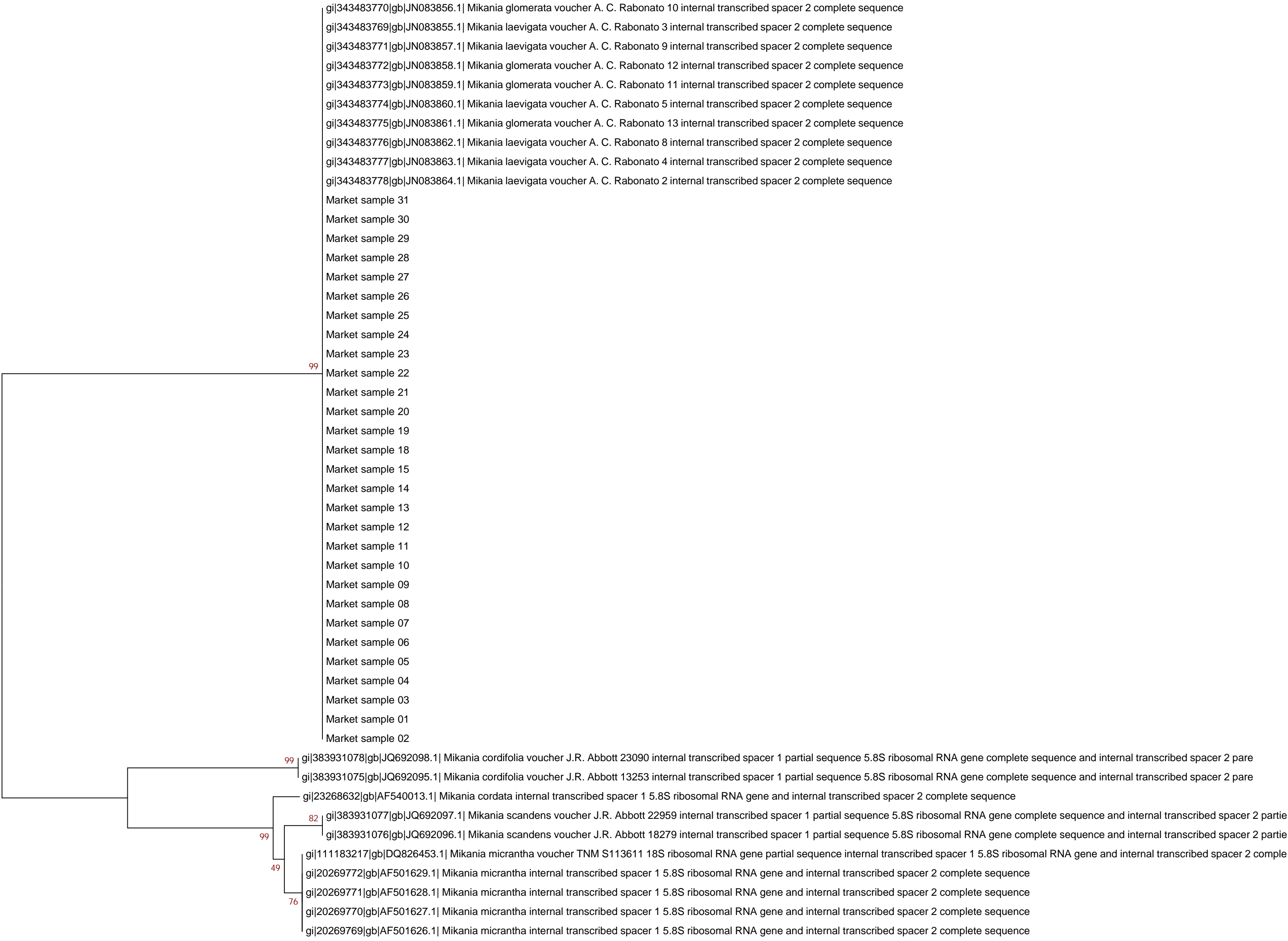

0.02

Supplement: S18 Fig — The evolutionary history was inferred using the Neighbor-Joining method. The optimal tree with the sum of branch length = 0.21453668 is shown. The percentage of replicate trees in which the associated taxa clustered together in the bootstrap test (500 replicates) are shown next to the branches. The tree is drawn to scale, with branch lengths in the same units as those of the evolutionary distances used to infer the phylogenetic tree. The evolutionary distances were computed using the Maximum Composite Likelihood method and are in the units of the number of base substitutions per site. The analysis involved 49 nucleotide sequences. Codon positions included were 1st+2nd+3rd+Noncoding. All positions containing gaps and missing data were eliminated. There were a total of 220 positions in the final dataset. Evolutionary analyses were conducted in MEGA5. (PDF) [file pone.0127866.s018.pdf]

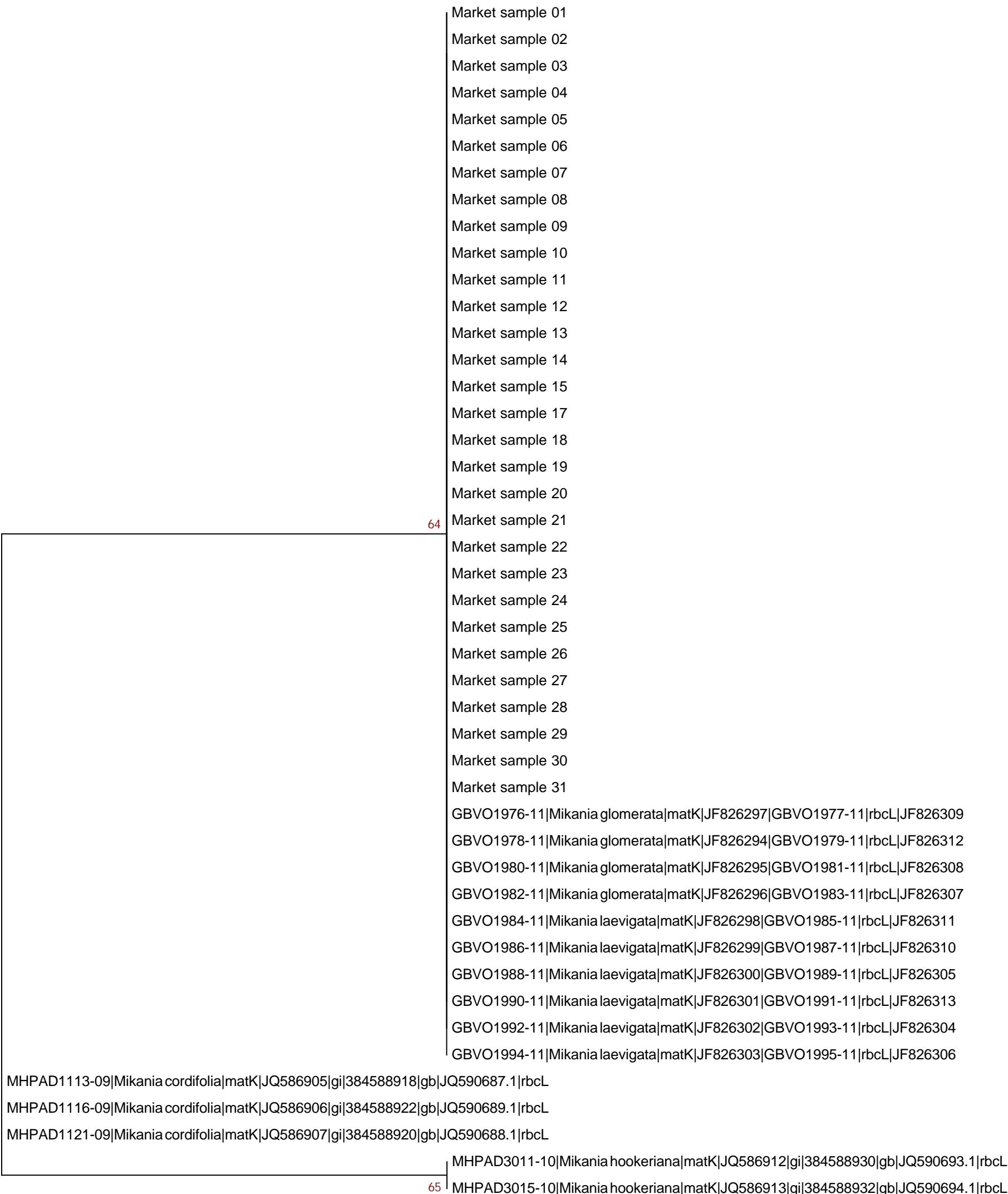

64

65

0.0001

Supplement: S19 Fig — The evolutionary history was inferred using the Neighbor-Joining method. The optimal tree with the sum of branch length = 0.00165371 is shown. The percentage of replicate trees in which the associated taxa clustered together in the bootstrap test (500 replicates) are shown next to the branches. The tree is drawn to scale, with branch lengths in the same units as those of the evolutionary distances used to infer the phylogenetic tree. The evolutionary distances were computed using the Maximum Composite Likelihood method and are in the units of the number of base substitutions per site. The analysis involved 45 nucleotide sequences. Codon positions included were 1st+2nd+3rd+Noncoding. All positions containing gaps and missing data were eliminated. There were a total of 1210 positions in the final dataset. Evolutionary analyses were conducted in MEGA5. (PDF) [file pone.0127866.s019.pdf]

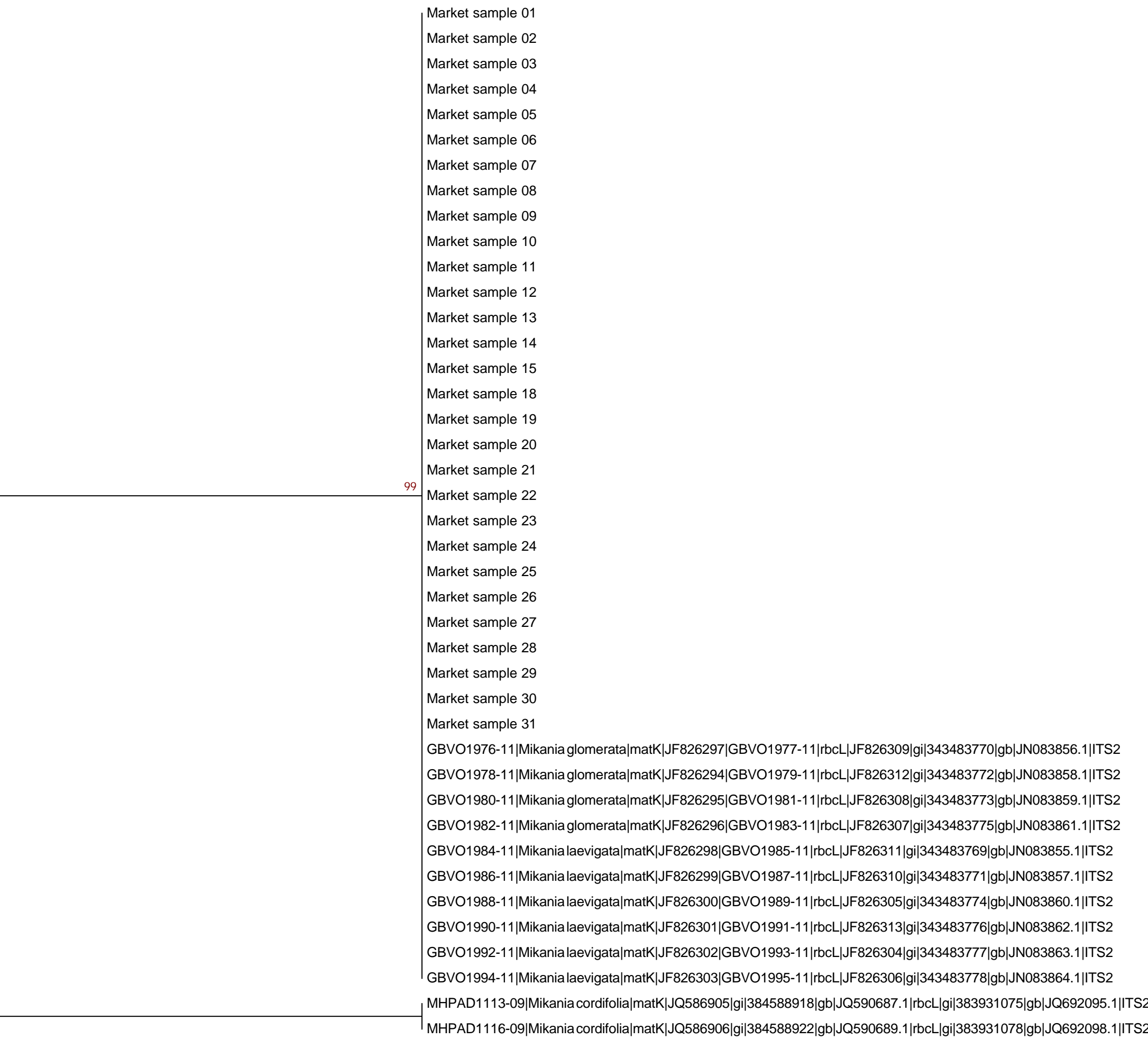

0.002

Supplement: S20 Fig — The evolutionary history was inferred using the Neighbor-Joining method. The optimal tree with the sum of branch length = 0.02267872 is shown. The percentage of replicate trees in which the associated taxa clustered together in the bootstrap test (500 replicates) are shown next to the branches. The tree is drawn to scale, with branch lengths in the same units as those of the evolutionary distances used to infer the phylogenetic tree. The evolutionary distances were computed using the Maximum Composite Likelihood method and are in the units of the number of base substitutions per site. The analysis involved 41 nucleotide sequences. Codon positions included were 1st+2nd+3rd+Noncoding. All positions containing gaps and missing data were eliminated. There were a total of 1435 positions in the final dataset. Evolutionary analyses were conducted in MEGA5. (PDF) [file pone.0127866.s020.pdf]

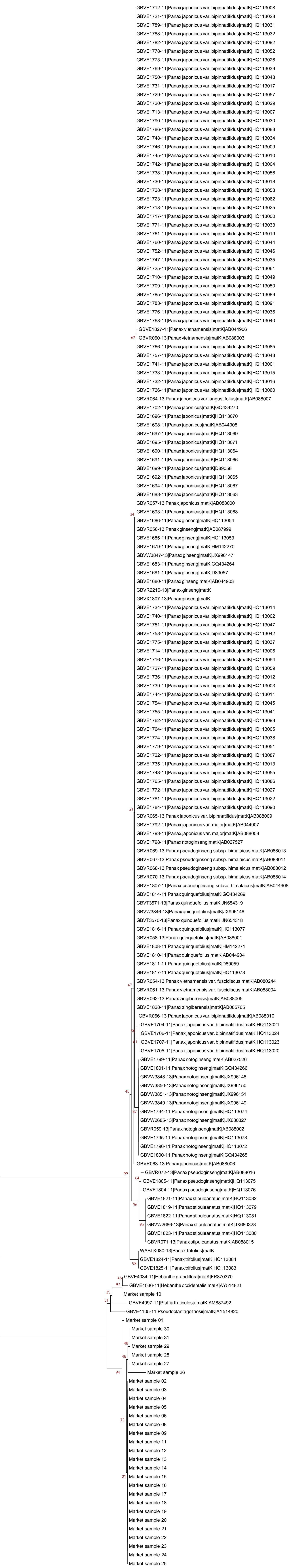

Supplement: S21 Fig — The evolutionary history was inferred using the Neighbor-Joining method. The optimal tree with the sum of branch length = 0.24952737 is shown. The percentage of replicate trees in which the associated taxa clustered together in the bootstrap test (500 replicates) are shown next to the branches. The tree is drawn to scale, with branch lengths in the same units as those of the evolutionary distances used to infer the phylogenetic tree. The evolutionary distances were computed using the Maximum Composite Likelihood method and are in the units of the number of base substitutions per site. The analysis involved 180 nucleotide sequences. Codon positions included were 1st+2nd+3rd+Noncoding. All positions containing gaps and missing data were eliminated. There were a total of 490 positions in the final dataset. Evolutionary analyses were conducted in MEGA5. (PDF) [file pone.0127866.s021.pdf]

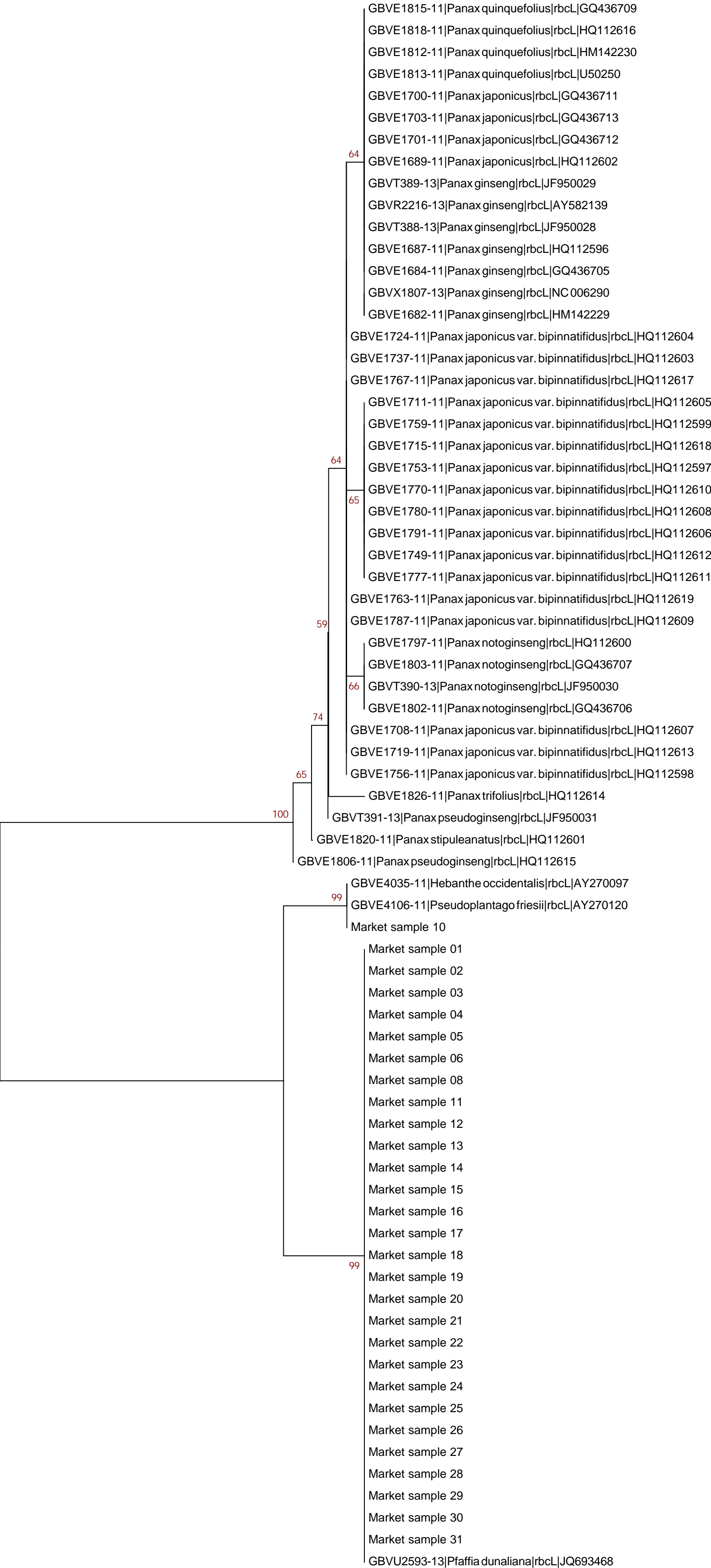

0.01

Supplement: S22 Fig — The evolutionary history was inferred using the Neighbor-Joining method. The optimal tree with the sum of branch length = 0.10182289 is shown. The percentage of replicate trees in which the associated taxa clustered together in the bootstrap test (500 replicates) are shown next to the branches. The tree is drawn to scale, with branch lengths in the same units as those of the evolutionary distances used to infer the phylogenetic tree. The evolutionary distances were computed using the Maximum Composite Likelihood method and are in the units of the number of base substitutions per site. The analysis involved 72 nucleotide sequences. Codon positions included were 1st+2nd+3rd+Noncoding. All positions containing gaps and missing data were eliminated. There were a total of 360 positions in the final dataset. Evolutionary analyses were conducted in MEGA5. (PDF) [file pone.0127866.s022.pdf]

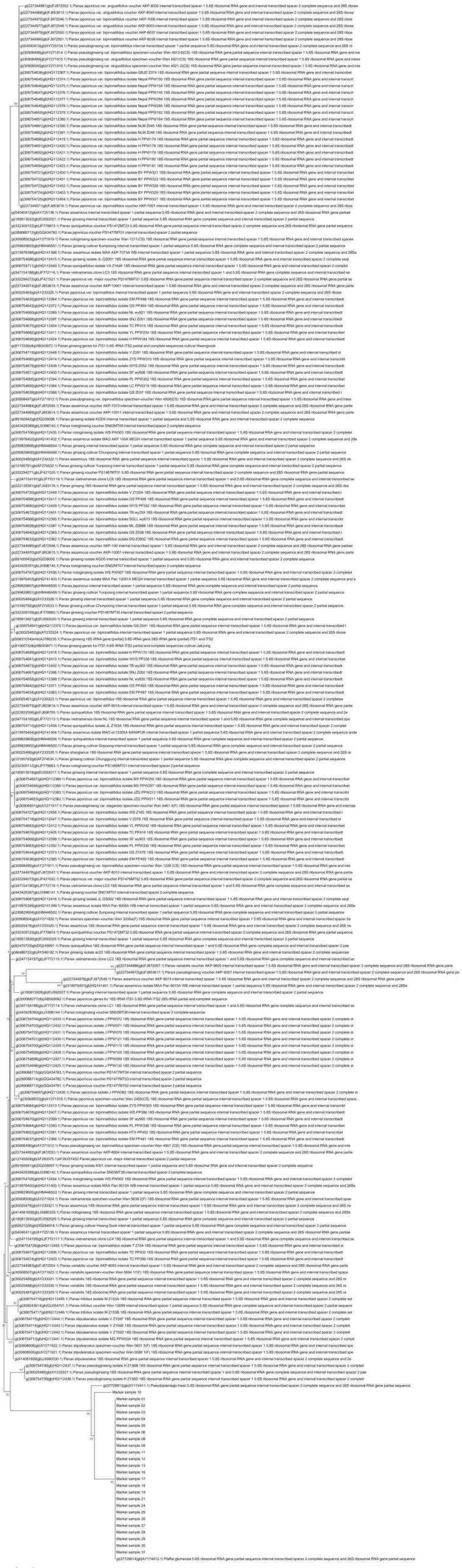

Supplement: S23 Fig — The evolutionary history was inferred using the Neighbor-Joining method. The optimal tree with the sum of branch length = 1,22004815 is shown. The percentage of replicate trees in which the associated taxa clustered together in the bootstrap test (500 replicates) are shown next to the branches. The tree is drawn to scale, with branch lengths in the same units as those of the evolutionary distances used to infer the phylogenetic tree. The evolutionary distances were computed using the Maximum Composite Likelihood method and are in the units of the number of base substitutions per site. The analysis involved 234 nucleotide sequences. Codon positions included were 1st+2nd+3rd+Noncoding. All positions containing gaps and missing data were eliminated. There were a total of 78 positions in the final dataset. Evolutionary analyses were conducted in MEGA5. (PDF) [file pone.0127866.s023.pdf]

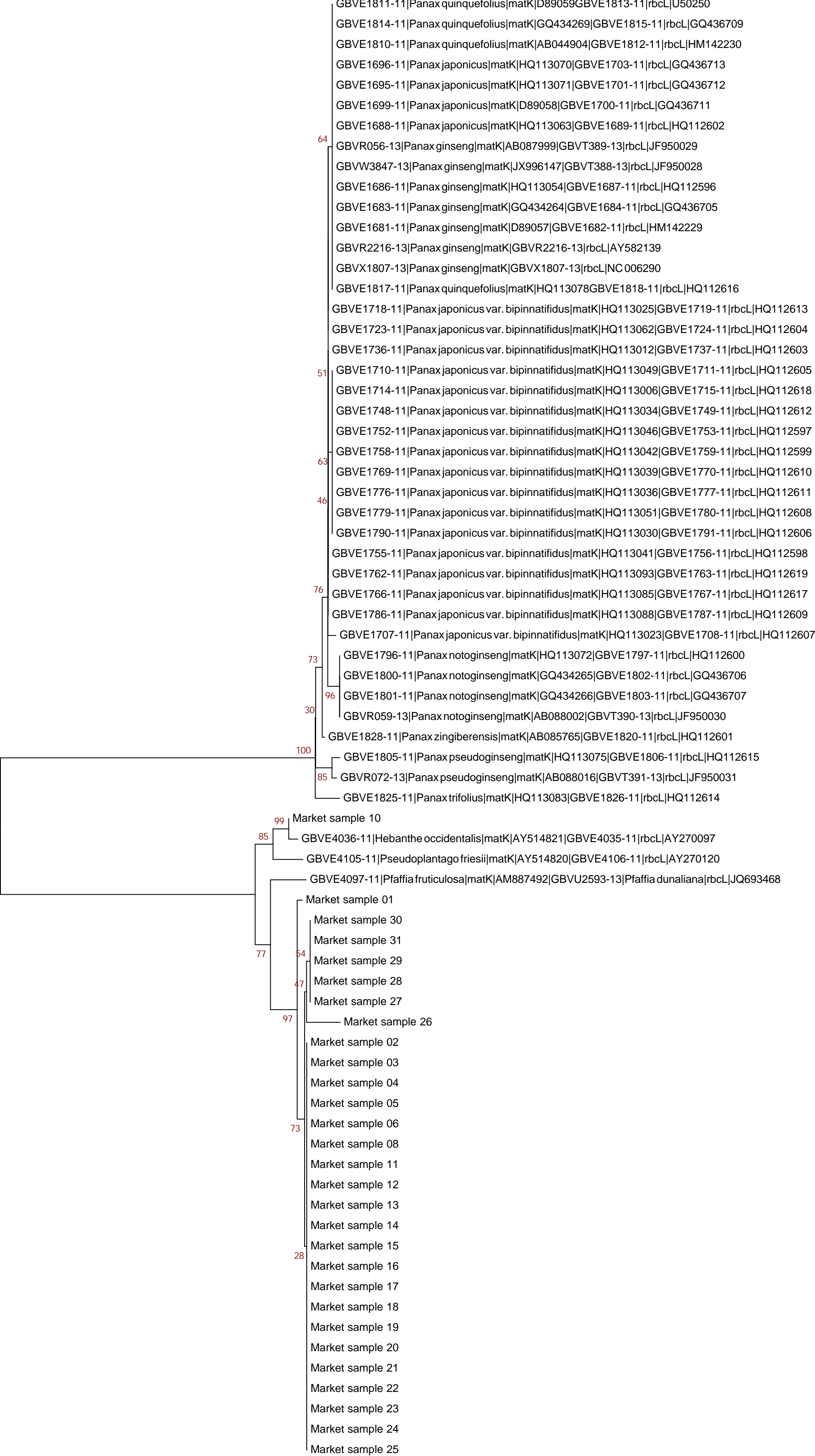

0.02

Supplement: S24 Fig — The evolutionary history was inferred using the Neighbor-Joining method. The optimal tree with the sum of branch length = 0.27861795 is shown. The percentage of replicate trees in which the associated taxa clustered together in the bootstrap test (500 replicates) are shown next to the branches. The tree is drawn to scale, with branch lengths in the same units as those of the evolutionary distances used to infer the phylogenetic tree. The evolutionary distances were computed using the Maximum Composite Likelihood method and are in the units of the number of base substitutions per site. The analysis involved 72 nucleotide sequences. Codon positions included were 1st+2nd+3rd+Noncoding. All positions containing gaps and missing data were eliminated. There were a total of 850 positions in the final dataset. Evolutionary analyses were conducted in MEGA5. (PDF) [file pone.0127866.s024.pdf]

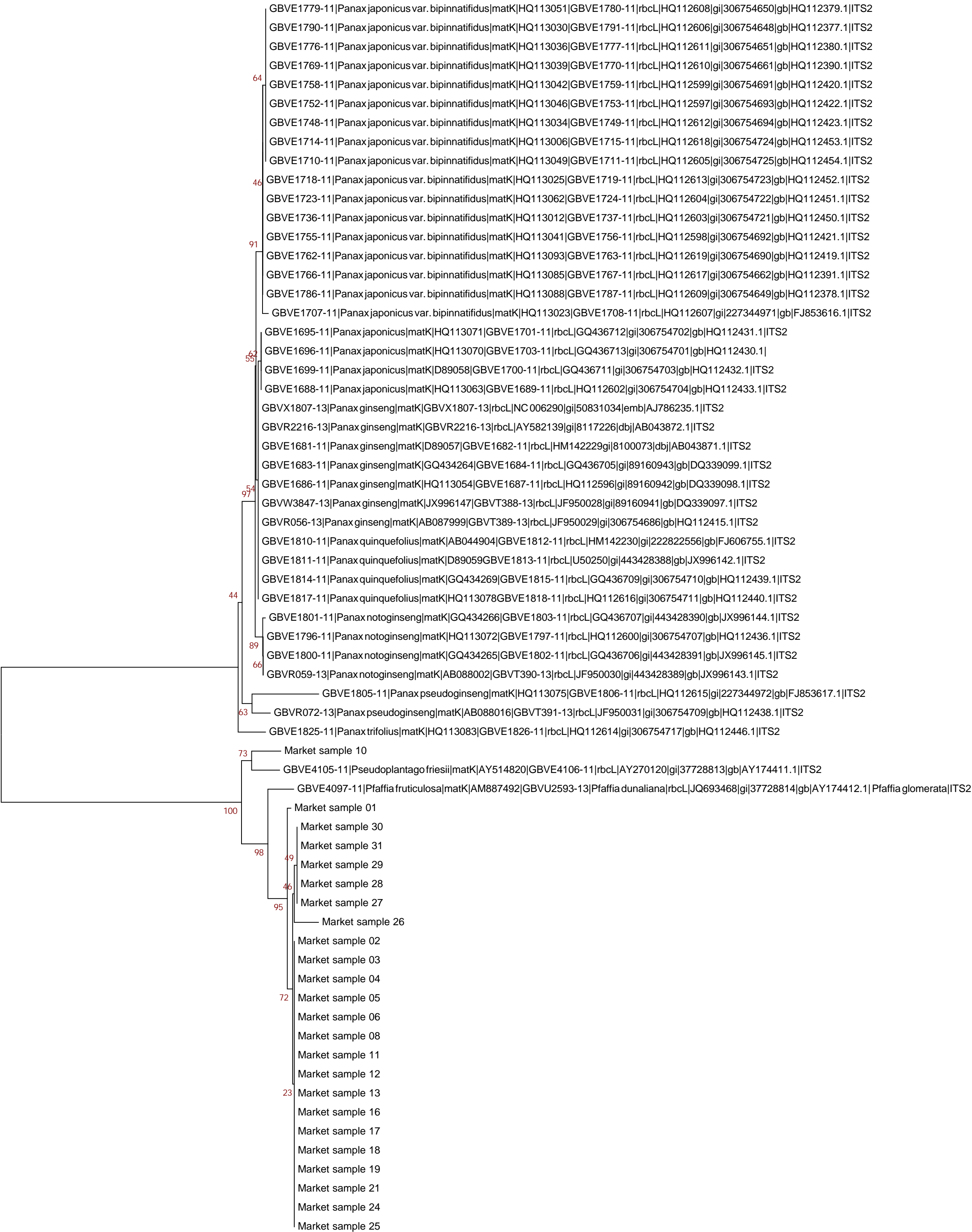

Supplement: S25 Fig — The evolutionary history was inferred using the Neighbor-Joining method. The optimal tree with the sum of branch length = 0.29889235 is shown. The percentage of replicate trees in which the associated taxa clustered together in the bootstrap test (500 replicates) are shown next to the branches. The tree is drawn to scale, with branch lengths in the same units as those of the evolutionary distances used to infer the phylogenetic tree. The evolutionary distances were computed using the Maximum Composite Likelihood method and are in the units of the number of base substitutions per site. The analysis involved 65 nucleotide sequences. Codon positions included were 1st+2nd+3rd+Noncoding. All positions containing gaps and missing data were eliminated. There were a total of 935 positions in the final dataset. Evolutionary analyses were conducted in MEGA5. (PDF) [file pone.0127866.s025.pdf]

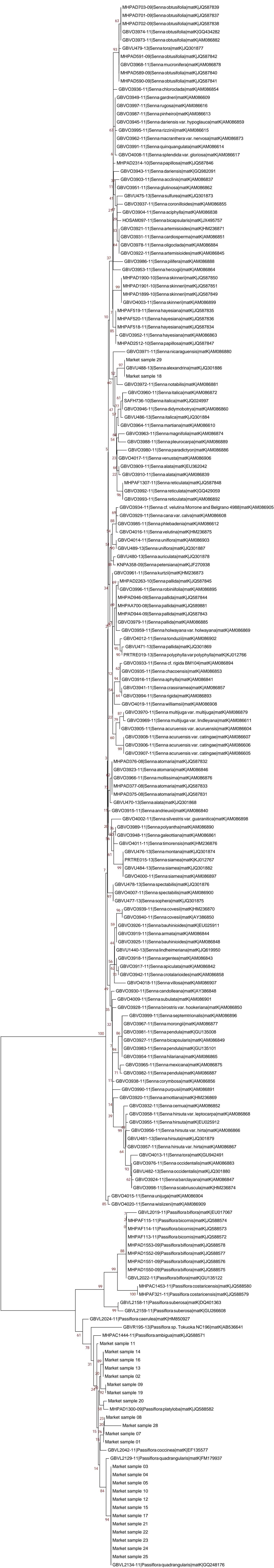

0.02

Supplement: S26 Fig — The evolutionary history was inferred using the Neighbor-Joining method. The optimal tree with the sum of branch length = 0.83753081 is shown. The percentage of replicate trees in which the associated taxa clustered together in the bootstrap test (500 replicates) are shown next to the branches. The tree is drawn to scale, with branch lengths in the same units as those of the evolutionary distances used to infer the phylogenetic tree. The evolutionary distances were computed using the Kimura 2-parameter method and are in the units of the number of base substitutions per site. The analysis involved 191 nucleotide sequences. Codon positions included were 1st+2nd+3rd+Noncoding. All positions containing gaps and missing data were eliminated. There were a total of 530 positions in the final dataset. Evolutionary analyses were conducted in MEGA5. (PDF) [file pone.0127866.s026.pdf]

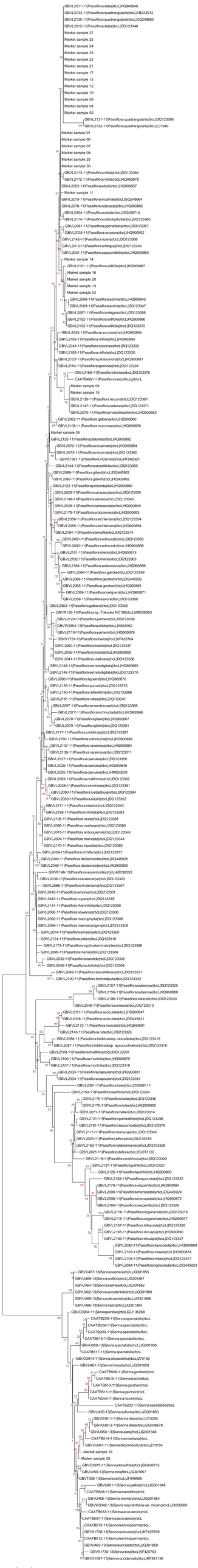

Supplement: S27 Fig — The evolutionary history was inferred using the Neighbor-Joining method. The optimal tree with the sum of branch length = 0.64321259 is shown. The percentage of replicate trees in which the associated taxa clustered together in the bootstrap test (500 replicates) are shown next to the branches. The tree is drawn to scale, with branch lengths in the same units as those of the evolutionary distances used to infer the phylogenetic tree. The evolutionary distances were computed using the Kimura 2-parameter method and are in the units of the number of base substitutions per site. The analysis involved 234 nucleotide sequences. Codon positions included were 1st+2nd+3rd+Noncoding. All positions containing gaps and missing data were eliminated. There were a total of 512 positions in the final dataset. Evolutionary analyses were conducted in MEGA5. (PDF) [file pone.0127866.s027.pdf]

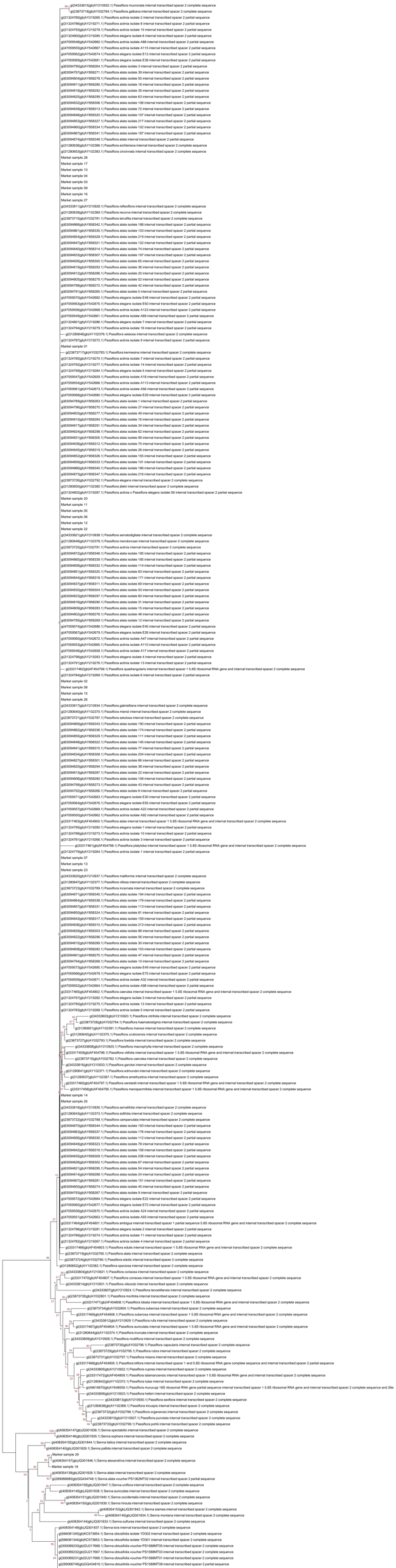

Supplement: S28 Fig — The evolutionary history was inferred using the Neighbor-Joining method. The optimal tree with the sum of branch length = 2.20614488 is shown. The percentage of replicate trees in which the associated taxa clustered together in the bootstrap test (500 replicates) are shown next to the branches. The tree is drawn to scale, with branch lengths in the same units as those of the evolutionary distances used to infer the phylogenetic tree. The evolutionary distances were computed using the Kimura 2-parameter method and are in the units of the number of base substitutions per site. The analysis involved 259 nucleotide sequences. Codon positions included were 1st+2nd+3rd+Noncoding. All positions containing gaps and missing data were eliminated. There were a total of 77 positions in the final dataset. Evolutionary analyses were conducted in MEGA5. (PDF) [file pone.0127866.s028.pdf]

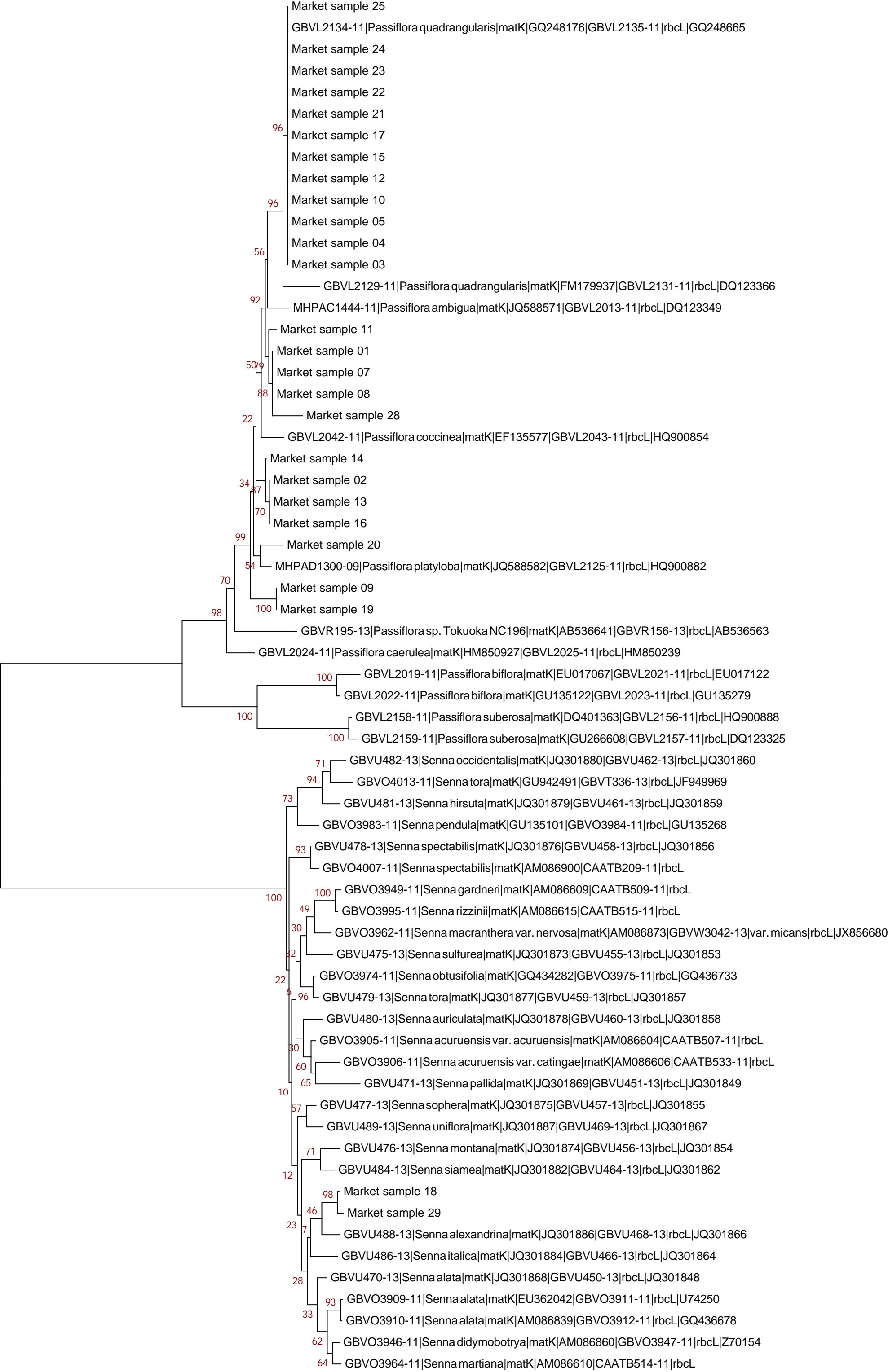

0.02

Supplement: S29 Fig — The evolutionary history was inferred using the Neighbor-Joining method. The optimal tree with the sum of branch length = 0.46000144 is shown. The percentage of replicate trees in which the associated taxa clustered together in the bootstrap test (500 replicates) are shown next to the branches. The tree is drawn to scale, with branch lengths in the same units as those of the evolutionary distances used to infer the phylogenetic tree. The evolutionary distances were computed using the Kimura 2-parameter method and are in the units of the number of base substitutions per site. The analysis involved 64 nucleotide sequences. Codon positions included were 1st+2nd+3rd+Noncoding. All positions containing gaps and missing data were eliminated. There were a total of 1045 positions in the final dataset. Evolutionary analyses were conducted in MEGA5. (PDF) [file pone.0127866.s029.pdf]

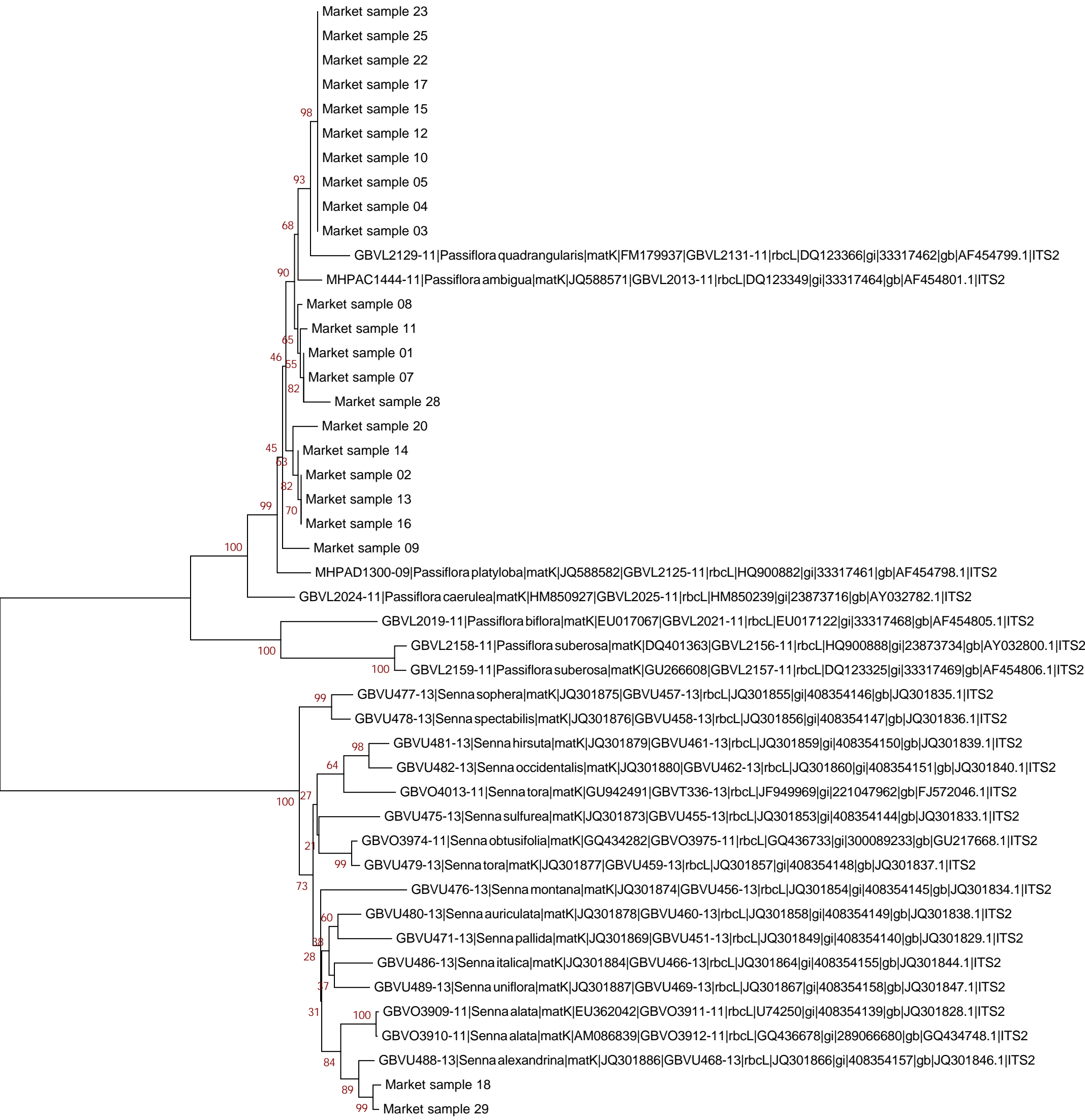

0.02

Supplement: S30 Fig — The evolutionary history was inferred using the Neighbor-Joining method. The optimal tree with the sum of branch length = 0.49341320 is shown. The percentage of replicate trees in which the associated taxa clustered together in the bootstrap test (500 replicates) are shown next to the branches. The tree is drawn to scale, with branch lengths in the same units as those of the evolutionary distances used to infer the phylogenetic tree. The evolutionary distances were computed using the Kimura 2-parameter method and are in the units of the number of base substitutions per site. The analysis involved 46 nucleotide sequences. Codon positions included were 1st+2nd+3rd+Noncoding. All positions containing gaps and missing data were eliminated. There were a total of 1191 positions in the final dataset. Evolutionary analyses were conducted in MEGA5. (PDF) [file pone.0127866.s030.pdf]

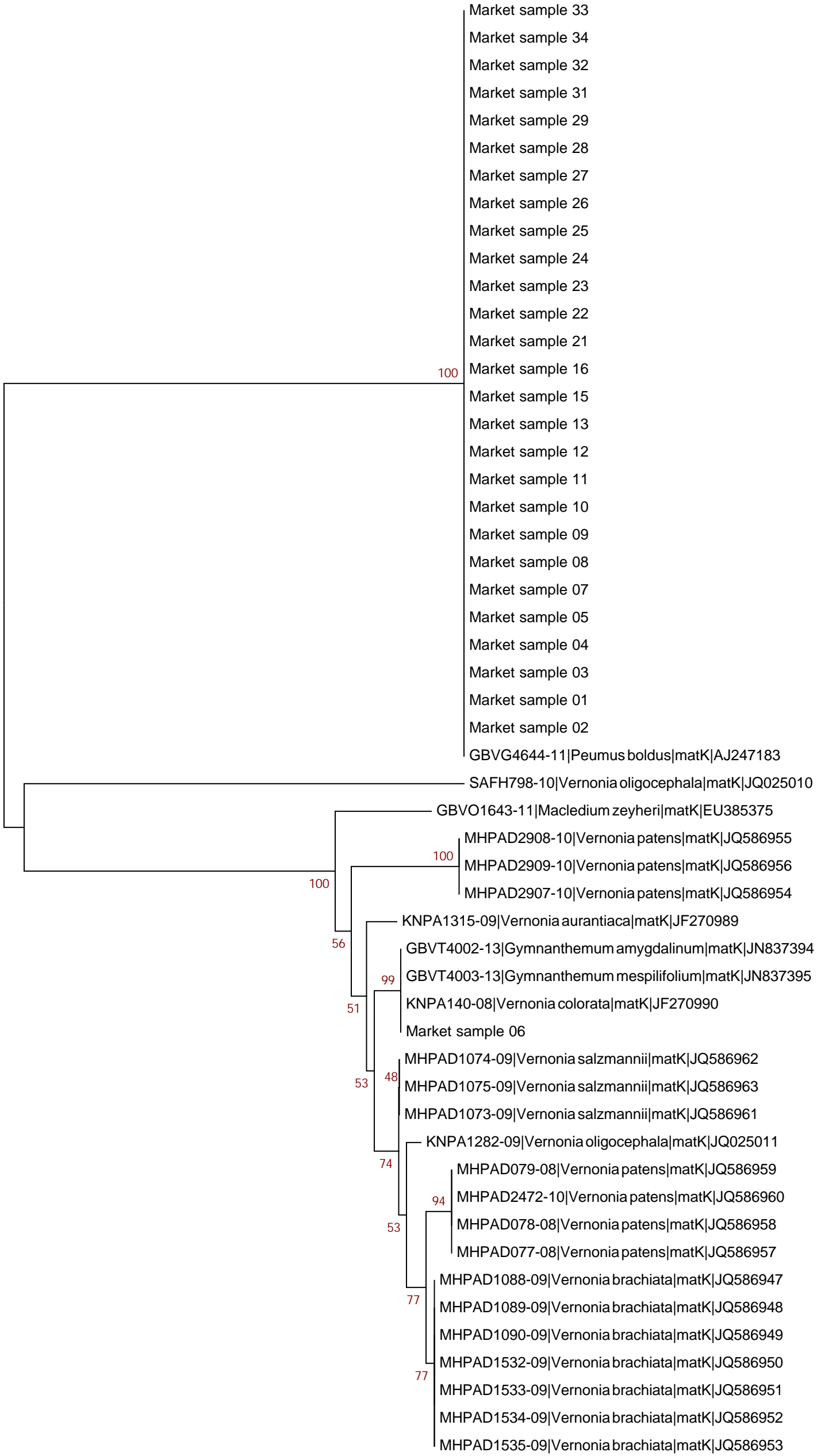

0.02

Supplement: S31 Fig — The evolutionary history was inferred using the Neighbor-Joining method. The optimal tree with the sum of branch length = 0.46390557 is shown. The percentage of replicate trees in which the associated taxa clustered together in the bootstrap test (500 replicates) are shown next to the branches. The tree is drawn to scale, with branch lengths in the same units as those of the evolutionary distances used to infer the phylogenetic tree. The evolutionary distances were computed using the Kimura 2-parameter method and are in the units of the number of base substitutions per site. The analysis involved 53 nucleotide sequences. Codon positions included were 1st+2nd+3rd+Noncoding. All positions containing gaps and missing data were eliminated. There were a total of 421 positions in the final dataset. Evolutionary analyses were conducted in MEGA5. (PDF) [file pone.0127866.s031.pdf]

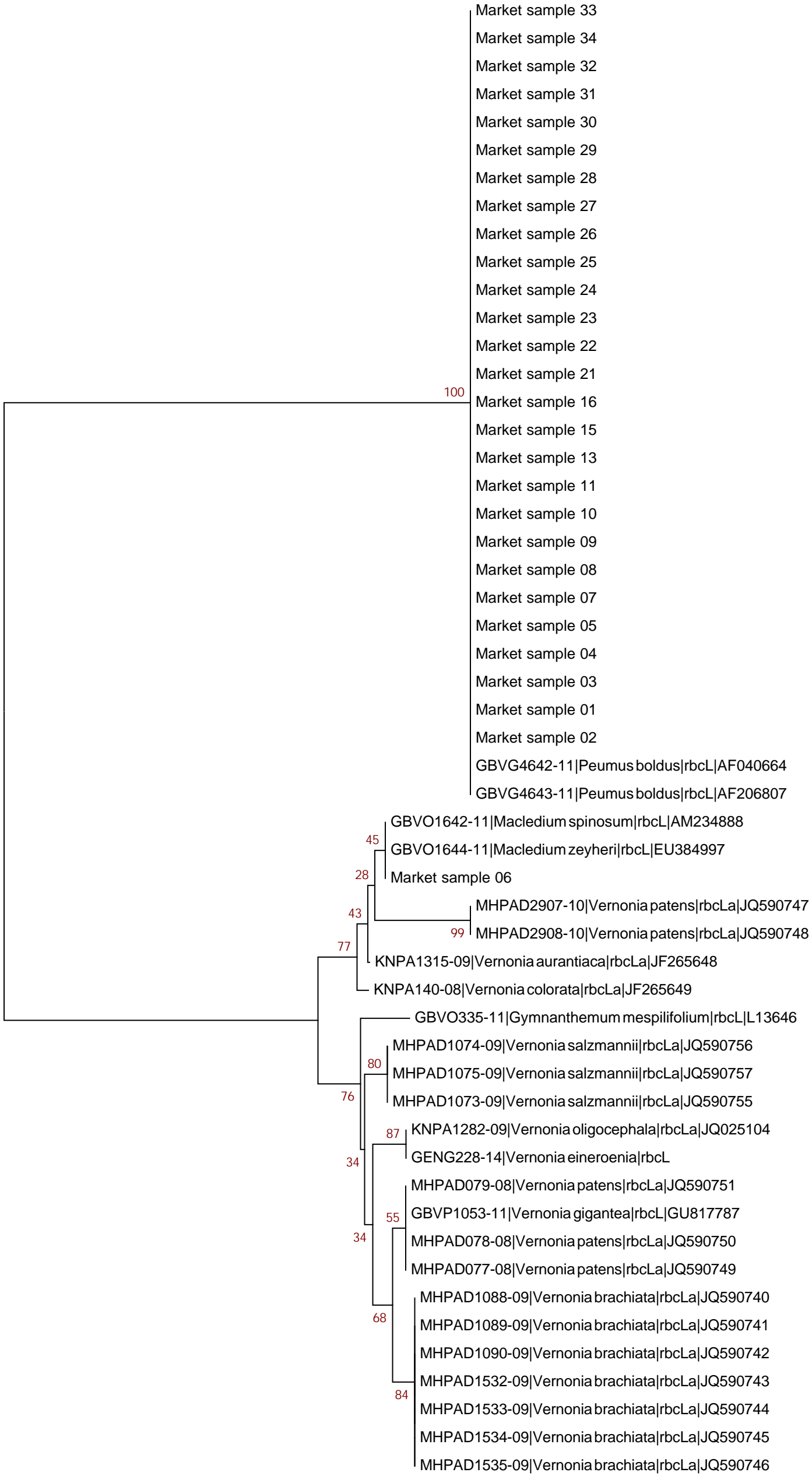

0.01

Supplement: S32 Fig — The evolutionary history was inferred using the Neighbor-Joining method. The optimal tree with the sum of branch length = 0.13298911 is shown. The percentage of replicate trees in which the associated taxa clustered together in the bootstrap test (500 replicates) are shown next to the branches. The tree is drawn to scale, with branch lengths in the same units as those of the evolutionary distances used to infer the phylogenetic tree. The evolutionary distances were computed using the Kimura 2-parameter method and are in the units of the number of base substitutions per site. The analysis involved 53 nucleotide sequences. Codon positions included were 1st+2nd+3rd+Noncoding. All positions containing gaps and missing data were eliminated. There were a total of 502 positions in the final dataset. Evolutionary analyses were conducted in MEGA5. (PDF) [file pone.0127866.s032.pdf]

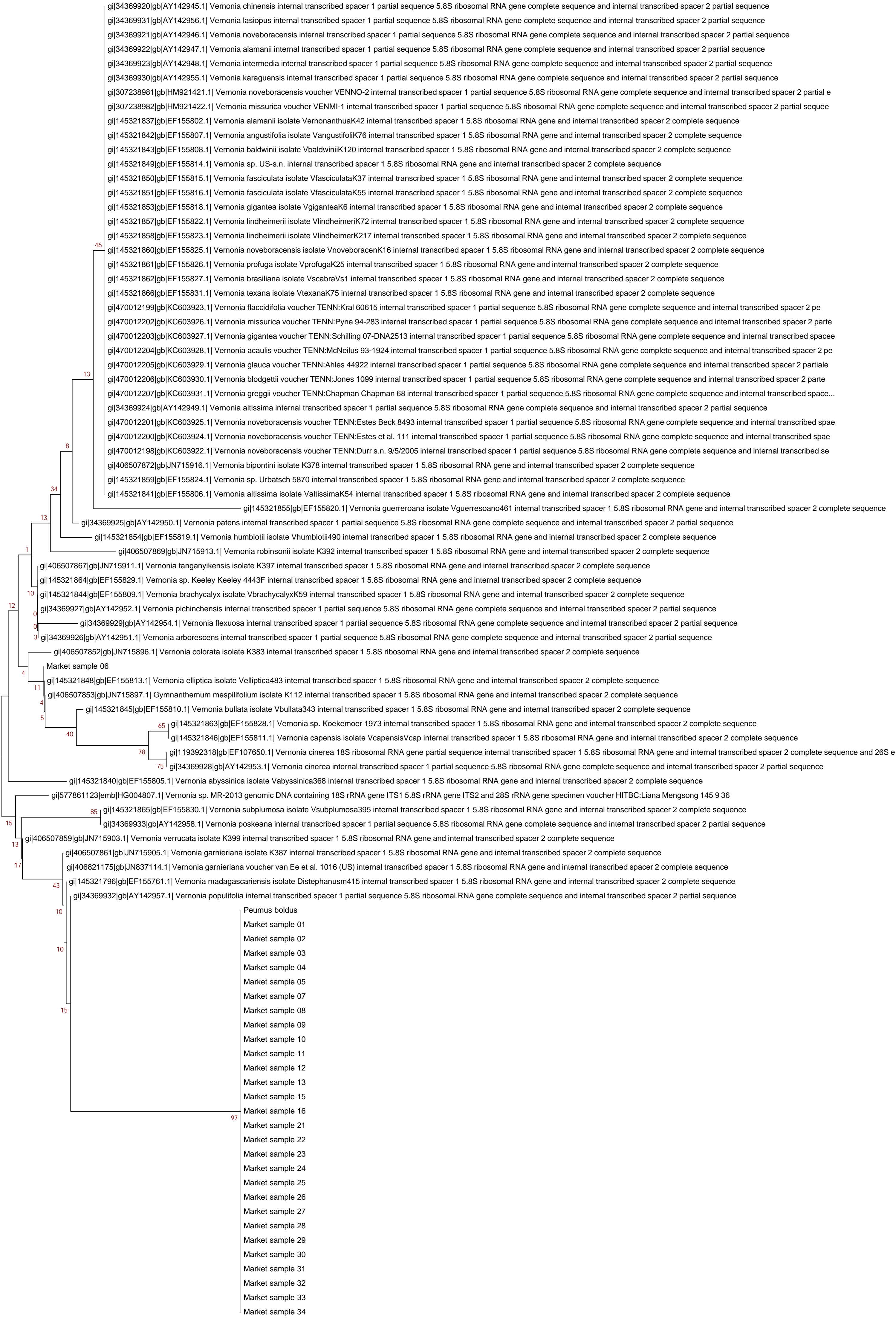

0.01

Supplement: S33 Fig — The evolutionary history was inferred using the Neighbor-Joining method. The optimal tree with the sum of branch length = 0.40028624 is shown. The percentage of replicate trees in which the associated taxa clustered together in the bootstrap test (500 replicates) are shown next to the branches. The tree is drawn to scale, with branch lengths in the same units as those of the evolutionary distances used to infer the phylogenetic tree. The evolutionary distances were computed using the Kimura 2-parameter method and are in the units of the number of base substitutions per site. The analysis involved 92 nucleotide sequences. Codon positions included were 1st+2nd+3rd+Noncoding. All positions containing gaps and missing data were eliminated. There were a total of 65 positions in the final dataset. Evolutionary analyses were conducted in MEGA5. (PDF) [file pone.0127866.s033.pdf]

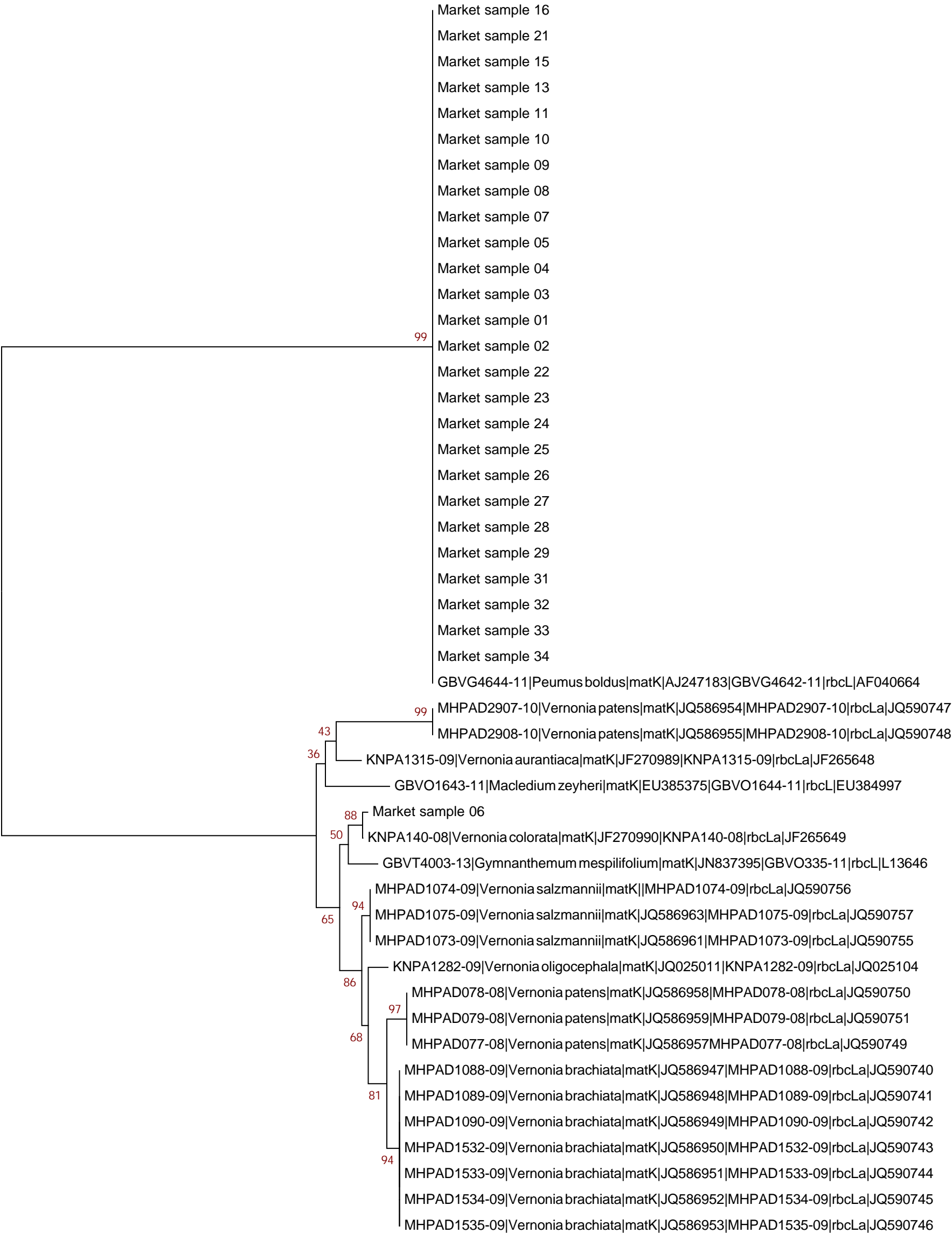

0.02

Supplement: S34 Fig — The evolutionary history was inferred using the Neighbor-Joining method. The optimal tree with the sum of branch length = 0.22642831 is shown. The percentage of replicate trees in which the associated taxa clustered together in the bootstrap test (500 replicates) are shown next to the branches. The tree is drawn to scale, with branch lengths in the same units as those of the evolutionary distances used to infer the phylogenetic tree. The evolutionary distances were computed using the Kimura 2-parameter method and are in the units of the number of base substitutions per site. The analysis involved 48 nucleotide sequences. Codon positions included were 1st+2nd+3rd+Noncoding. All positions containing gaps and missing data were eliminated. There were a total of 928 positions in the final dataset. Evolutionary analyses were conducted in MEGA5. (PDF) [file pone.0127866.s034.pdf]

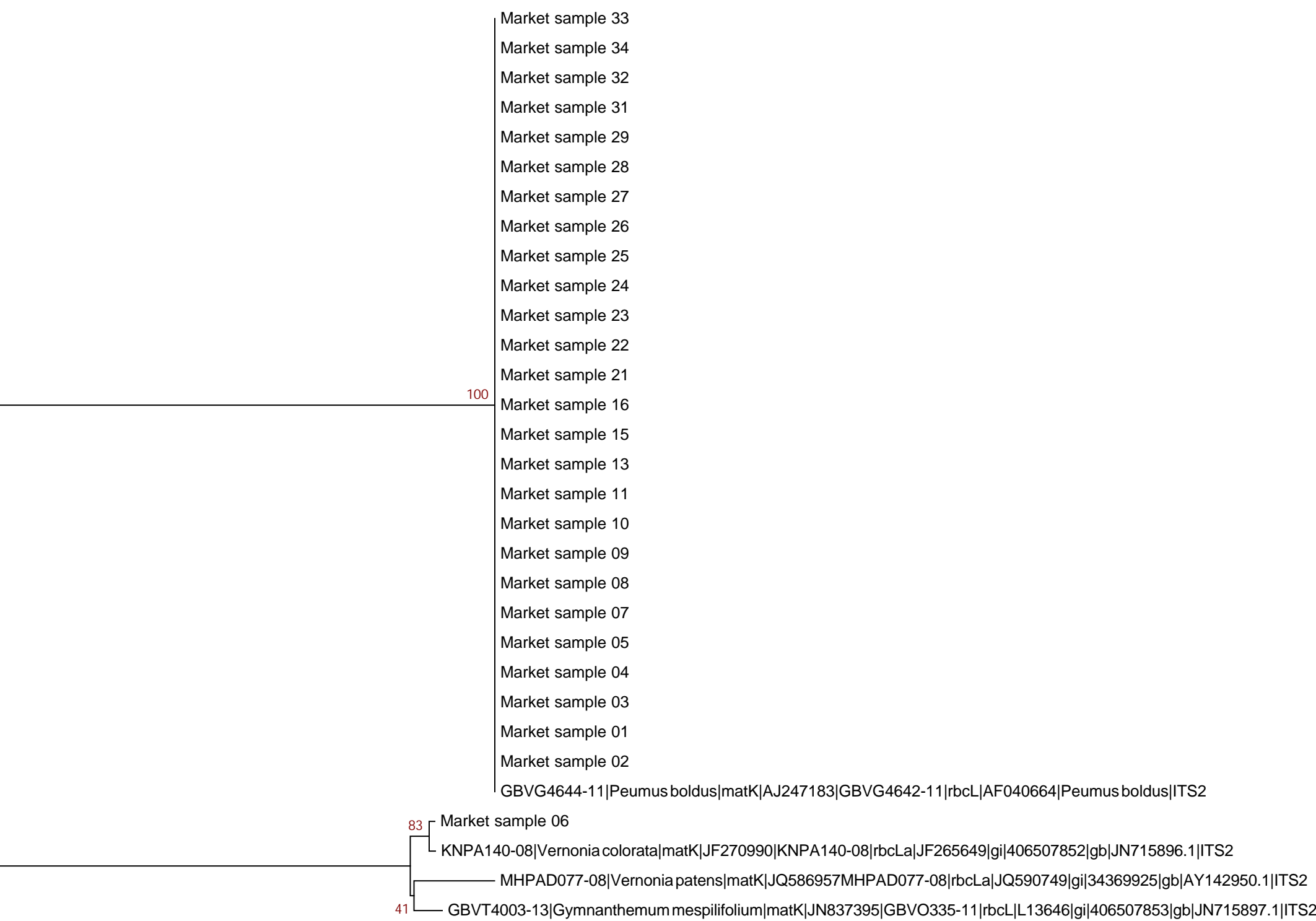

0.02

Supplement: S35 Fig — The evolutionary history was inferred using the Neighbor-Joining method. The optimal tree with the sum of branch length = 0.17636441 is shown. The percentage of replicate trees in which the associated taxa clustered together in the bootstrap test (500 replicates) are shown next to the branches. The tree is drawn to scale, with branch lengths in the same units as those of the evolutionary distances used to infer the phylogenetic tree. The evolutionary distances were computed using the Kimura 2-parameter method and are in the units of the number of base substitutions per site. The analysis involved 31 nucleotide sequences. Codon positions included were 1st+2nd+3rd+Noncoding. All positions containing gaps and missing data were eliminated. There were a total of 998 positions in the final dataset. Evolutionary analyses were conducted in MEGA5. (PDF) [file pone.0127866.s035.pdf]

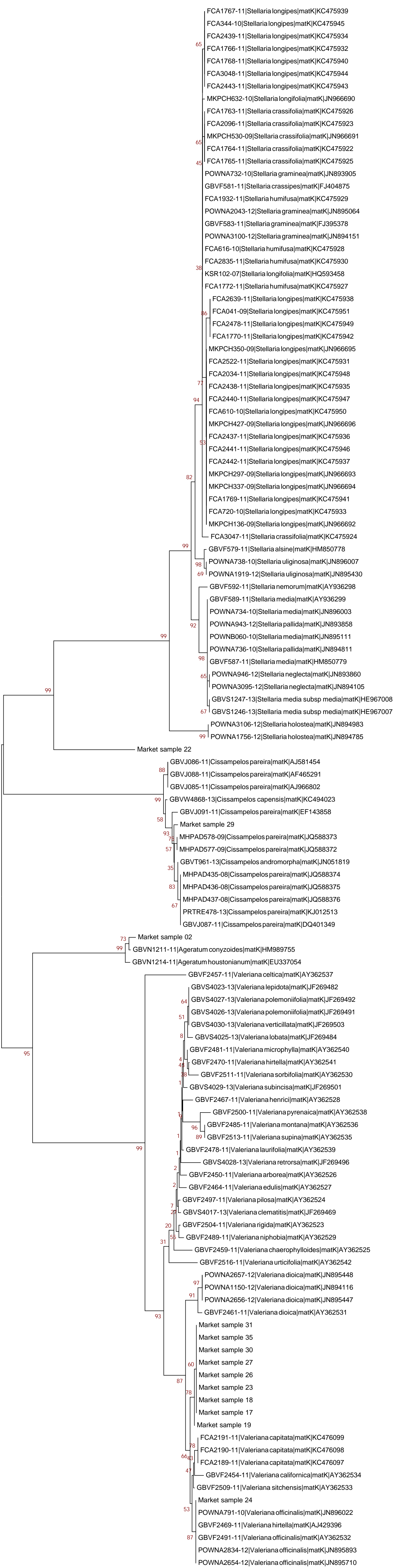

Supplement: S36 Fig — The evolutionary history was inferred using the Neighbor-Joining method. The optimal tree with the sum of branch length = 1.06223373 is shown. The percentage of replicate trees in which the associated taxa clustered together in the bootstrap test (500 replicates) are shown next to the branches. The tree is drawn to scale, with branch lengths in the same units as those of the evolutionary distances used to infer the phylogenetic tree. The evolutionary distances were computed using the Kimura 2-parameter method and are in the units of the number of base substitutions per site. The analysis involved 125 nucleotide sequences. Codon positions included were 1st+2nd+3rd+Noncoding. All positions containing gaps and missing data were eliminated. There were a total of 525 positions in the final dataset. Evolutionary analyses were conducted in MEGA5. (PDF) [file pone.0127866.s036.pdf]

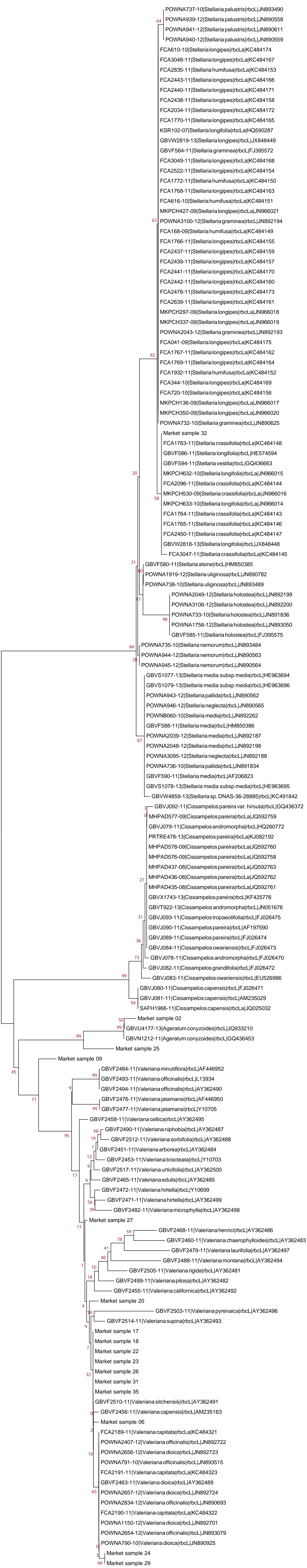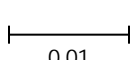

Supplement: S37 Fig — The evolutionary history was inferred using the Neighbor-Joining method. The optimal tree with the sum of branch length = 0.45263449 is shown. The percentage of replicate trees in which the associated taxa clustered together in the bootstrap test (500 replicates) are shown next to the branches. The tree is drawn to scale, with branch lengths in the same units as those of the evolutionary distances used to infer the phylogenetic tree. The evolutionary distances were computed using the Kimura 2-parameter method and are in the units of the number of base substitutions per site. The analysis involved 155 nucleotide sequences. Codon positions included were 1st+2nd+3rd+Noncoding. All positions containing gaps and missing data were eliminated. There were a total of 474 positions in the final dataset. Evolutionary analyses were conducted in MEGA5. (PDF) [file pone.0127866.s037.pdf]

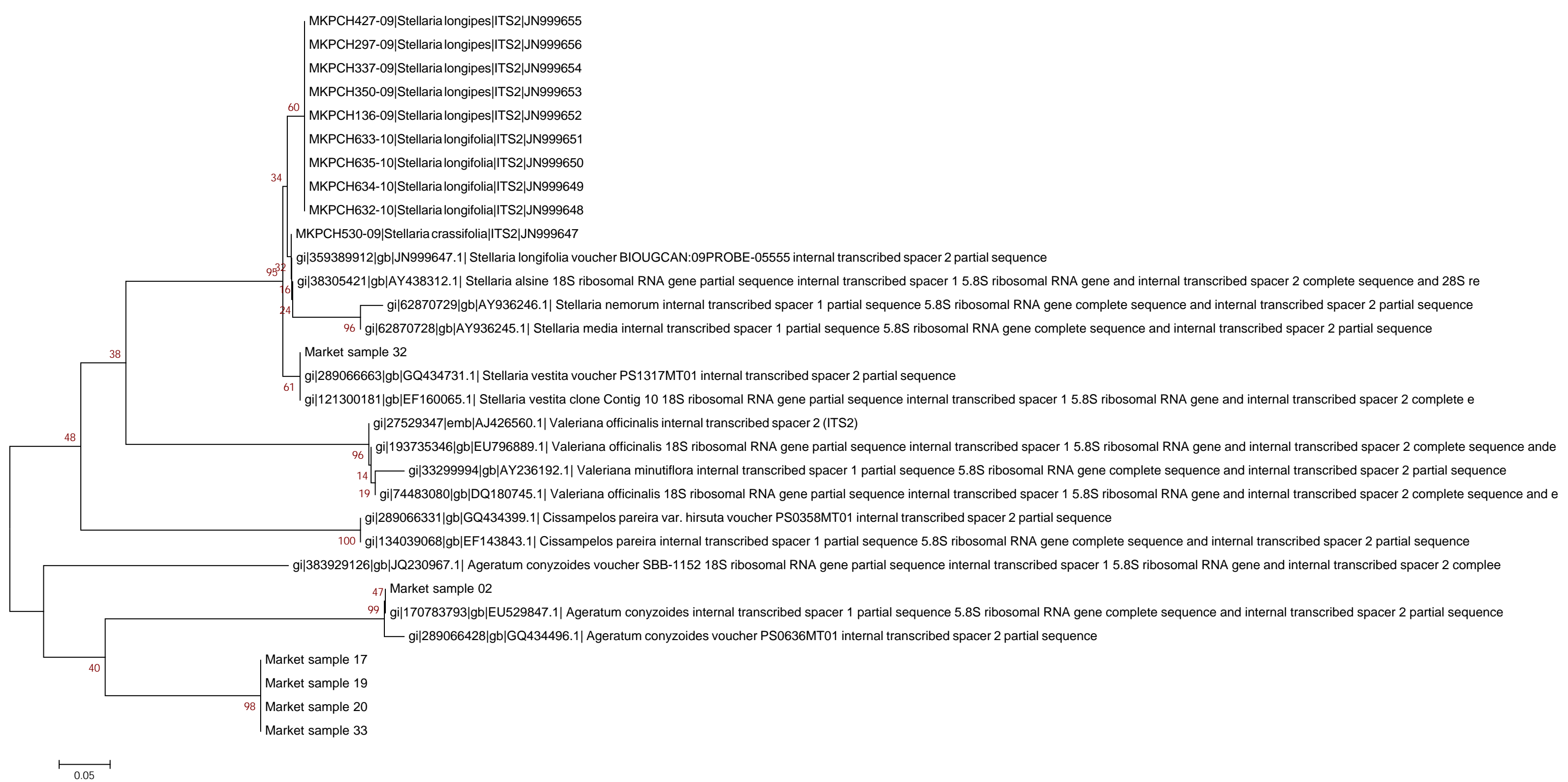

Supplement: S38 Fig — The evolutionary history was inferred using the Neighbor-Joining method. The optimal tree with the sum of branch length = 1.70998580 is shown. The percentage of replicate trees in which the associated taxa clustered together in the bootstrap test (500 replicates) are shown next to the branches. The tree is drawn to scale, with branch lengths in the same units as those of the evolutionary distances used to infer the phylogenetic tree. The evolutionary distances were computed using the Kimura 2-parameter method and are in the units of the number of base substitutions per site. The analysis involved 31 nucleotide sequences. Codon positions included were 1st+2nd+3rd+Noncoding. All positions containing gaps and missing data were eliminated. There were a total of 50 positions in the final dataset. Evolutionary analyses were conducted in MEGA5. (PDF) [file pone.0127866.s038.pdf]

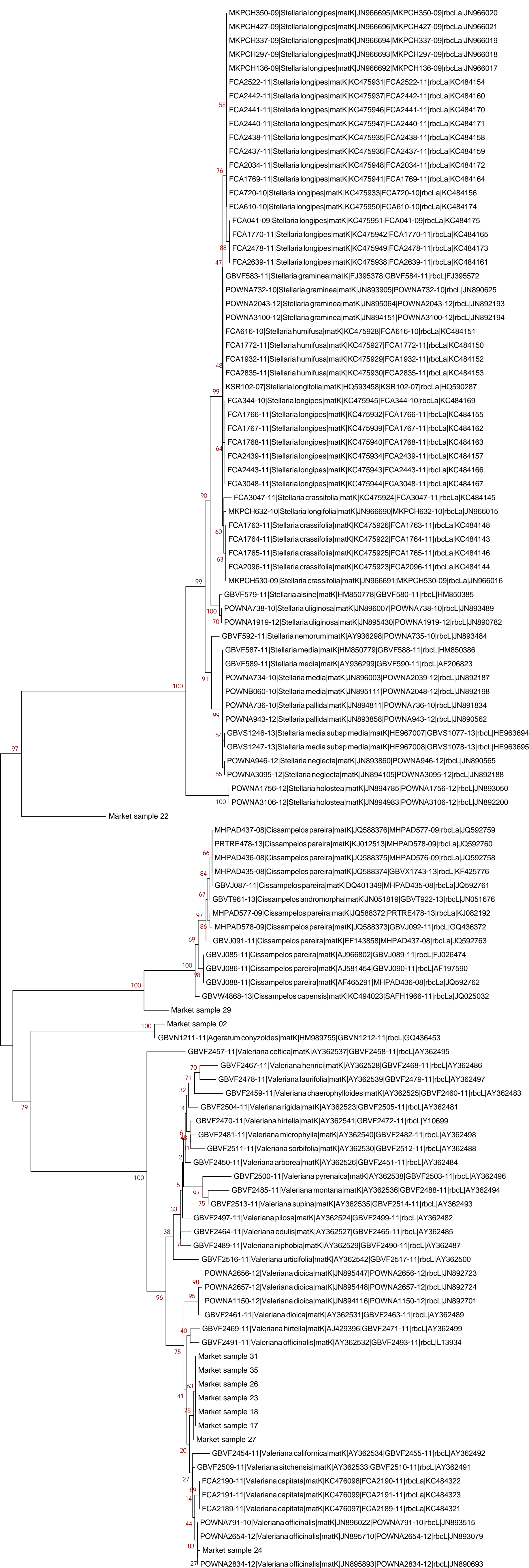

Supplement: S39 Fig — The evolutionary history was inferred using the Neighbor-Joining method. The optimal tree with the sum of branch length = 0.74255654 is shown. The percentage of replicate trees in which the associated taxa clustered together in the bootstrap test (500 replicates) are shown next to the branches. The tree is drawn to scale, with branch lengths in the same units as those of the evolutionary distances used to infer the phylogenetic tree. The evolutionary distances were computed using the Kimura 2-parameter method and are in the units of the number of base substitutions per site. The analysis involved 113 nucleotide sequences. Codon positions included were 1st+2nd+3rd+Noncoding. All positions containing gaps and missing data were eliminated. There were a total of 1001 positions in the final dataset. Evolutionary analyses were conducted in MEGA5. (PDF) [file pone.0127866.s039.pdf]

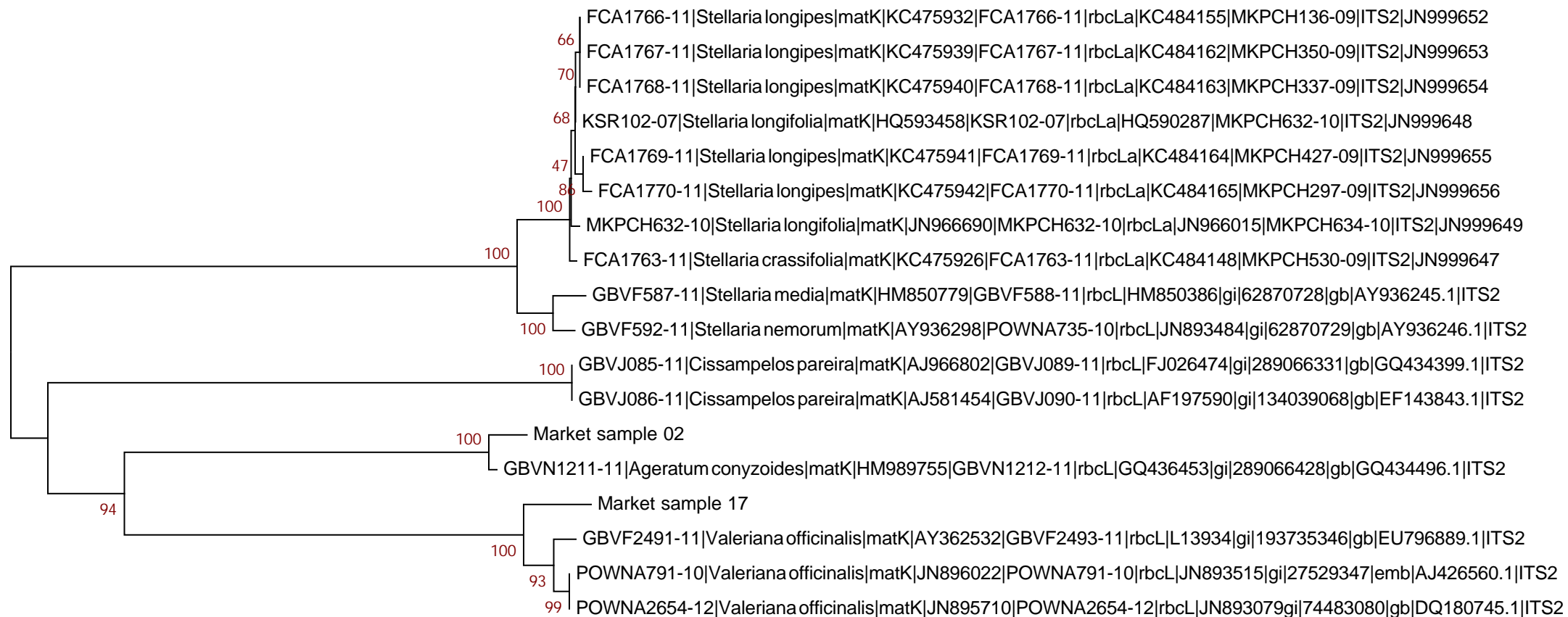

0.02

Supplement: S40 Fig — The evolutionary history was inferred using the Neighbor-Joining method. The optimal tree with the sum of branch length = 0.48881377 is shown. The percentage of replicate trees in which the associated taxa clustered together in the bootstrap test (500 replicates) are shown next to the branches. The tree is drawn to scale, with branch lengths in the same units as those of the evolutionary distances used to infer the phylogenetic tree. The evolutionary distances were computed using the Kimura 2-parameter method and are in the units of the number of base substitutions per site. The analysis involved 18 nucleotide sequences. Codon positions included were 1st+2nd+3rd+Noncoding. All positions containing gaps and missing data were eliminated. There were a total of 1121 positions in the final dataset. Evolutionary analyses were conducted in MEGA5. (PDF) [file pone.0127866.s040.pdf]
