# Supplementary material for: Medicinal Plants Recommended by the World Health Organization: DNA Barcode Identification Associated with Chemical Analyses Guarantees Their Quality
Source: PLoS One. 2015 May 15;10(5):e0127866. doi: 10.1371/journal.pone.0127866 (PMC4433216; doi:10.1371/journal.pone.0127866)
Supplement: S7 Fig — The evolutionary history was inferred using the Neighbor-Joining method. The optimal tree with the sum of branch length = 0.00211645 is shown. The percentage of replicate trees in which the associated taxa clustered together in the bootstrap test (500 replicates) are shown next to the branches. The tree is drawn to scale, with branch lengths in the same units as those of the evolutionary distances used to infer the phylogenetic tree. The evolutionary distances were computed using the Maximum Composite Likelihood method and are in the units of the number of base substitutions per site. The analysis involved 36 nucleotide sequences. Codon positions included were 1st+2nd+3rd+Noncoding. All positions containing gaps and missing data were eliminated. There were a total of 473 positions in the final dataset. Evolutionary analyses were conducted in MEGA5. (PDF) [file pone.0127866.s007.pdf]

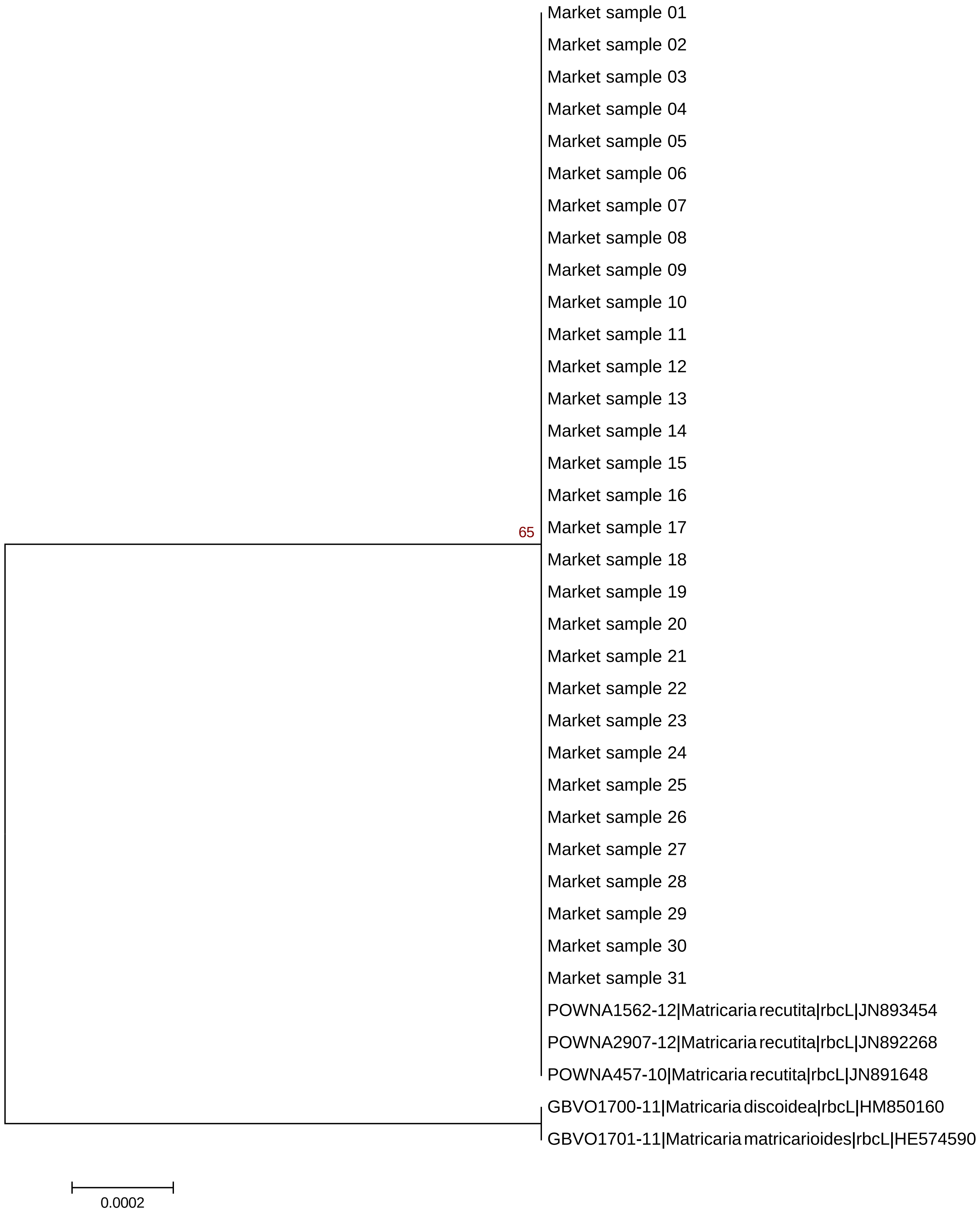

Market sample 01
Market sample 02
Market sample 03
Market sample 04
Market sample 05
Market sample 06
Market sample 07
Market sample 08
Market sample 09
Market sample 10
Market sample 11
Market sample 12
Market sample 13
Market sample 14
Market sample 15
Market sample 16
65
Market sample 17
Market sample 18
Market sample 19
Market sample 20
Market sample 21
Market sample 22
Market sample 23
Market sample 24
Market sample 25
Market sample 26
Market sample 27
Market sample 28
Market sample 29
Market sample 30
Market sample 31
POWNA1562-12|Matricaria recutita|rbcL|JN893454
POWNA2907-12|Matricaria recutita|rbcL|JN892268
POWNA457-10|Matricaria recutita|rbcL|JN891648
GBVO1700-11|Matricaria discoidea|rbcL|HM850160
GBVO1701-11|Matricaria matricarioides|rbcL|HE574590
0.0002
